# A general and mild synthetic method for fused-ring electronic acceptors

**DOI:** 10.1126/sciadv.adp8150

**Published:** 2024-08-21

**Authors:** Xiaowei Zhong, Shubin Liu, Wei You

**Affiliations:** ^1^ Department of Applied Physical Sciences, University of North Carolina at Chapel Hill, Chapel Hill, NC 27599, USA.; ^2^ Department of Chemistry, University of North Carolina at Chapel Hill, Chapel Hill, NC 27599, USA.; ^3^ Research Computing Center, University of North Carolina at Chapel Hill, Chapel Hill, NC 27599, USA.

## Abstract

Fused-ring electronic acceptors (FREAs) have transformed the field of organic solar cells. However, the prevailing syntheses of FREAs suffer from low yield, difficulty in separation, and high cost. Here, we report new and streamlined syntheses with three distinctive key steps. First, a universal approach to fuse neighboring aromatic units via a single carbon atom is demonstrated with ytterbium triflate and boron trifluoride as the catalysts. This approach allows the incorporation of diverse side-chain combinations. Second, nitrogen atom fusing neighboring aromatics is realized by using oxo-molybdenum catalyst, featuring lower reaction temperatures and enhanced yields. Third, an organic catalyst, proline, is identified to catalyze the aldol condensation with high yield to afford the most typical FREAs having acceptor-donor-acceptor (ADA) configurations. Our new chemistries enable easy syntheses of a wide range of FREAs, substantially expanding the scope and availability of these coveted materials at reduced synthetic cost, particularly for organic electronics.

## Teaser

Streamlined synthesis of fused-ring electronic acceptor enables exploration of diverse structures for optoelectronics.

## INTRODUCTION

**Fig. 1.**
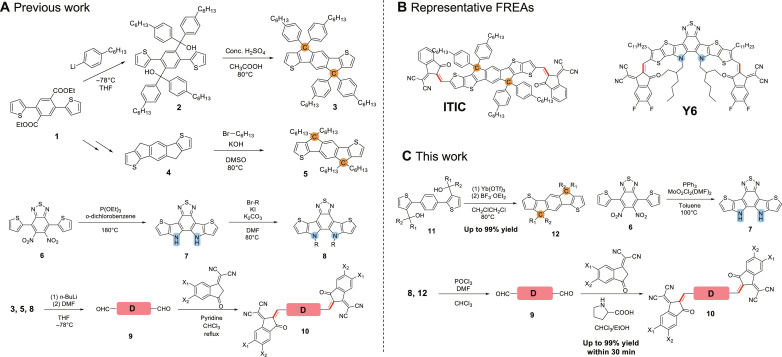
Synthetic routes to FREAs. (**A**) Previous works. (**B**) Two representative FREAs, ITIC and Y6. (**C**) Our approaches feature new catalysts, shorter reaction time, mild conditions, and easy separation (quantitative yields). DMSO, dimethyl sulfoxide; THF, tetrahydrofuran; EtOH, ethanol.

Fused-ring electronic acceptors (FREAs) such as ITIC (*1*) and Y6 (*2*) (structures in Fig. 1B) have been extensively used in bulk heterojunction (BHJ) solar cells to achieve high efficiencies (*3*–*5*). FREAs typically contain one electron-rich (D) core where several aromatic units are fused together, which is connected with two electron-deficient (A) end units via double bonds in an acceptor-donor-acceptor (ADA) configuration. Over the years, many structural changes to the core and the end units of FREAs have been explored to fine tune their optoelectronic properties. For example, cyclopentadiene units are used in the core of ITIC (fusing the neighboring aromatic units via a carbon atom), and pyrrole units are recruited for Y6 (fusing the neighbors via a nitrogen atom). A convergent approach is generally used to the synthesis of FREAs. For example, the synthesis of IDTIC series (Fig. 1A) (*6*, *7*) starts from a diester (**1**) which is nucleophilically attacked twice by the aryl lithium reagent to form the tertiary alcohol (**2**); treating this tertiary alcohol (**2**) with concentrated sulfuric acid can incur the cyclization/fusion with the flanking thiophenes to offer the core (**3**) (*8*). Since bulky aromatic side chains often lead to increased distance between FREAs in the solid state (not desirable for charge transport), alkyl chains have been used instead (*6*, *9*, *10*). However, the synthesis of such alkylated backbone is rather challenging since treating alkyl tertiary alcohols with strong acids could lead to the formation of alkene (via E1 elimination) rather than the desired fused-ring product. Therefore, in the reported synthesis of the dialkylated backbone, the diester (**1**) is converted to acid chloride first to use the Friedel-Crafts acylation for the cyclization. The carbonyls in the fused core are then reduced to methylene to afford (**4**), which is subsequently deprotonated by potassium hydroxide to create the nucleophile to attack alkyl bromide (twice) to acquire the fully alkylated core (**5**) (*11*). Unfortunately, the intermediates (e.g., **4**) are usually sparingly soluble, which essentially restricts this approach to small size cores. Furthermore, the final step of dialkylation to the unsubstituted core (e.g., **4**) often suffers from monoalkylation due to steric effects, which would cause notable structural defects in the final FREA (*12*). In cases where asymmetric side chains are desired, the synthesis would be even more complicated via a combination of both synthetic methods mentioned above (*9*). As for nitrogen-bridged cores (e.g., Y6), Cadogan-Sundberg indole synthesis is usually used with triethyl phosphite as the reductant, which requires high temperature and high–boiling point solvents (e.g., *o*-dichlorobenzene, 180°C) (*2*, *13*, *14*). Furthermore, in converting (**6**) to (**7**), the by-product—triethyl phosphate, an electrophilic, might give rise to N-substituted heterocycles, resulting in difficult separation (*15*–*17*). Subsequent alkylation of (**7**) would afford the alkylated core (**8**). In all cases, the core (**3**, **5**, or **8**) then goes through the usual deprotonation of these two alpha-Hs at terminal thiophenes, followed by quenching with *N*,*N*′-dimethylformamide (DMF) to form the target aldehyde. In the last step, the pyridine-catalyzed Knoevenagel condensation is typically used to connect the end groups (A units) to the backbone (D unit) to yield the target FREA; however, the often observed low yields of this step cause great difficulty in obtaining the pure FREA (*1*).

It is evident that the reported methods of FREAs have high synthetic complexities (*18*), posing a grand challenge to further develop this important class of materials; furthermore, the associated low yield and high cost thereof set a substantial barrier for commercializing BHJ solar cells. Thus, we set our goal to develop a generally applicable yet simple synthetic approach of FREAs that would not only allow for various structural modifications but also afford higher yield with minimal purification (and low cost).

## RESULTS

**Fig. 2.**
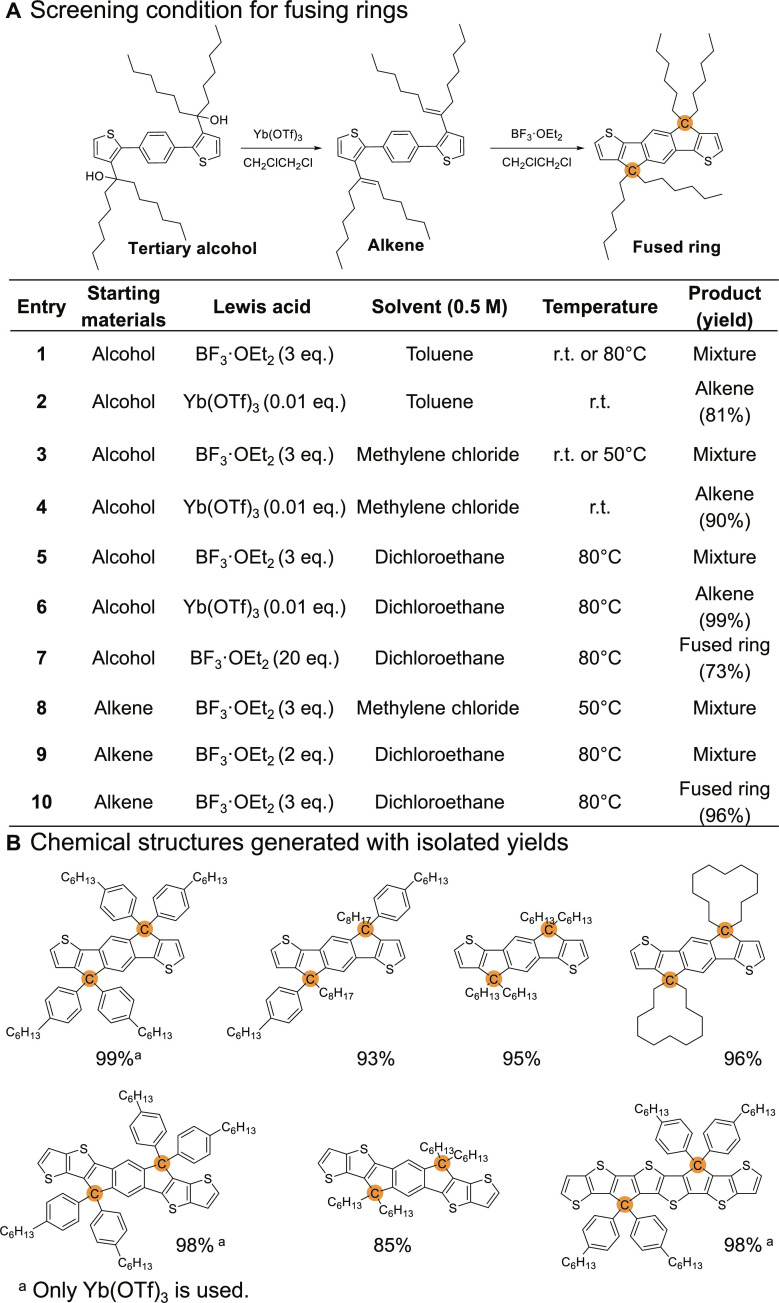
Fusing neighboring rings via carbon atom. (**A**) Screening the condition. (**B**) Chemical structures with yields using this methodology. r.t., room temperature.

The most challenging step in the syntheses of FREAs is to fuse neighboring aromatic units via a single atom (e.g., carbon or nitrogen), which thereby became our initial focus. To achieve C-bridged cyclization, most literature reports used concentrated sulfuric acid as the catalyst and acetic acid as the solvent; however, we discovered that this method could lead to incomplete cyclization, accompanied by a substantial amount of by-products (e.g., alkenes in the case of doubly alkylated tertiary alcohol). After screening several Lewis acids (table S1), ytterbium triflate [Yb(OTf)_3_], a mild Lewis acid, and BF_3_·OEt_2_ were identified for further evaluation. When side chains are aromatics (e.g., phenyl), 1 mol % Yb(OTf)_3_ can efficiently catalyze the cyclization in dichloroethane at 80°C. However, in the case of alkyl chains, alkenes (only one configuration) were obtained quantitatively with 1 mol % Yb(OTf)_3_ (solvent independent) (entries 2, 4, and 6 in Fig. 2A). Unexpectedly, this alkene could be converted to cyclized products quantitatively with 3 eq. BF_3_·OEt_2_ in dichloroethane (entry 10). Changing the solvent to methylene chloride (entry 8) or reducing the use of BF_3_·OEt_2_ to 2 eq. (entry 9) only produced a mixture of products. On the other hand, directly treating tertiary alcohols with 3 eq. BF_3_·OEt_2_ only gave mixed products regardless of the choice of solvents (toluene, methylene chloride, or dichloroethane) (entries 1, 3, and 5). Nevertheless, largely excess (20 eq.) amount of BF_3_·OEt_2_ could transform tertiary alcohols to the fused-ring product in dichloroethane at 80°C (entry 7). Considering these experimental results, we designed a two-step approach to obtain the cyclized product in the case of alkylated tertiary alcohol. Specifically, Yb(OTf)_3_ in dichloroethane at 80°C is first used to catalyze the quantitative formation of alkene; filtering off Yb(OTf)_3_ (reusable) affords the alkene in the filtrate. This filtrate (with dichloroethane as the solvent) is then added with 3 eq. of BF_3_·OEt_2_ at 80°C to finish the cyclization. Ethanol is used to quench the reaction, and the pure product (fused-ring) is obtained by filtering through a short path of silica gel to remove the boron compounds. We emphasize that this two-step method is mild and very effective to synthesize many common alkylated fused-ring moieties (some are shown in Fig. 2B).

Mechanistically, we propose that the formation of alkenes requires less energy than that of the cyclization. When Yb(OTf)_3_ is attached to the hydroxyl groups, β elimination (from the adjacent C─H) can occur to form the double bond (alkene). In the second step, BF_3_·OE_2_ is attached to the newly formed double bonds to generate a carbocation, which is then captured by the adjacent aromatic ring to accomplish the cyclization (fig. S2). Since carbocations need to be captured by the neighboring electron-rich aromatic units, electron-rich solvents, such as toluene, should be avoided to prevent unnecessary side reactions. In our case, dichloroethane at 80°C appears to be the best condition. Density functional theory calculation (fig. S2) supports this proposed mechanism, showing a much lower energy barrier in forming alkene from the tertiary alcohol than directly converting the tertiary alcohol to the ring-fused product. We note that large amount of BF_3_·OE_2_ (3 eq.) is used for each ring-fusing/cyclization rather than the catalytic amount of Yb(OTf)_3_; this substantial difference could be caused by the formation of BF_3_·H_2_O after the cyclization, which would deactivate the “catalyst” (BF_3_).

**Fig. 3.**
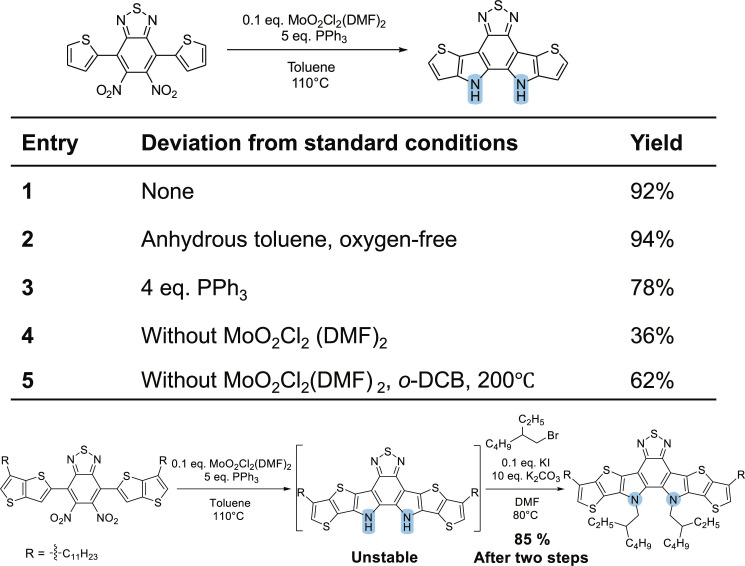
Screening reaction conditions for Cadogan-Sundberg indole synthesis to form the N-bridged core of FREAs. *o*-DCB, ortho-dichlorobenzene.

To achieve the N-bridged cyclization for making Y6, we selected an oxo-molybdenum catalyst after an extensive literature review (*16*, *17*). Specifically, MoO_2_Cl_2_(DMF)_2_ was prepared following established procedures without any additional purification steps (*19*). The use of this catalyst allows for lower reaction temperature (e.g., 110°C) and employment of triphenylphosphine (PPh_3_) as the reductant rather than triethyl phosphite; the use of PPh_3_ largely simplifies the reaction workup (*20*). Mechanistically, we propose that PPh_3_ first reduces Mo(VI) to Mo(IV), which subsequently reduces the nitro group to the nitroso group (fig. S3). Another PPh_3_ molecule further reduces the nitroso into a nitrene which subsequently undergoes a C─H insertion reaction with the neighboring aromatic ring, offering the cyclized product (*21*). With dinitro-benzothiadiazole flanked with two thiophenes as the substrate, we screened a variety of conditions and identified the optimal condition (Fig. 3). Briefly, when 5 eq. PPh_3_ are used, choosing anhydrous solvent and oxygen free condition only improved the yield slightly (entry 2); however, reducing the amount of PPh_3_ to 4 eq. led to a lower yield (entry 3). On the other hand, the absence of Mo catalyst would markedly lower the yield of the cyclization (entry 4), unless higher temperature and high boiling point solvent were applied (entry 5). With the optimized condition, the substrate containing flanking thiophene units offered excellent isolated yields (Fig. 3) after crystallization from ethanol; however, the one containing alkylated thienothiophenes could not be easily isolated. This product appeared to be highly sensitive to acidic conditions and air (likely oxygen). Nevertheless, if the subsequent N-alkylation step was performed immediately (in the same reaction flask), then the N-alkylated product could be achieved with a total yield of 85% after two steps. Notably, this new method to achieve N-cyclization should facilitate the exploration of FREAs based on indoles and carbazoles and other similarly structured organic moieties.

After completing the synthesis of the core, Vilsmeier-Haack formylation was adopted to anchor the two terminal aldehyde functional groups (Fig. 1C, from **8** to **9**) rather than the prevailing approach of deprotonation/quenching by DMF. The advantages of using V-H reaction in here are that (i) only the α-position of the aromatic unit was involved (*22*), and (ii) the reaction was almost quantitative. In our hands, the isolated yield of the formylation step of typically used cores (in FREAs) is always higher than 80%, independent of chemical nature of the core. Pure products can be obtained by filtration through a short path of silica gel or celite to remove the by-products.

**Fig. 4.**
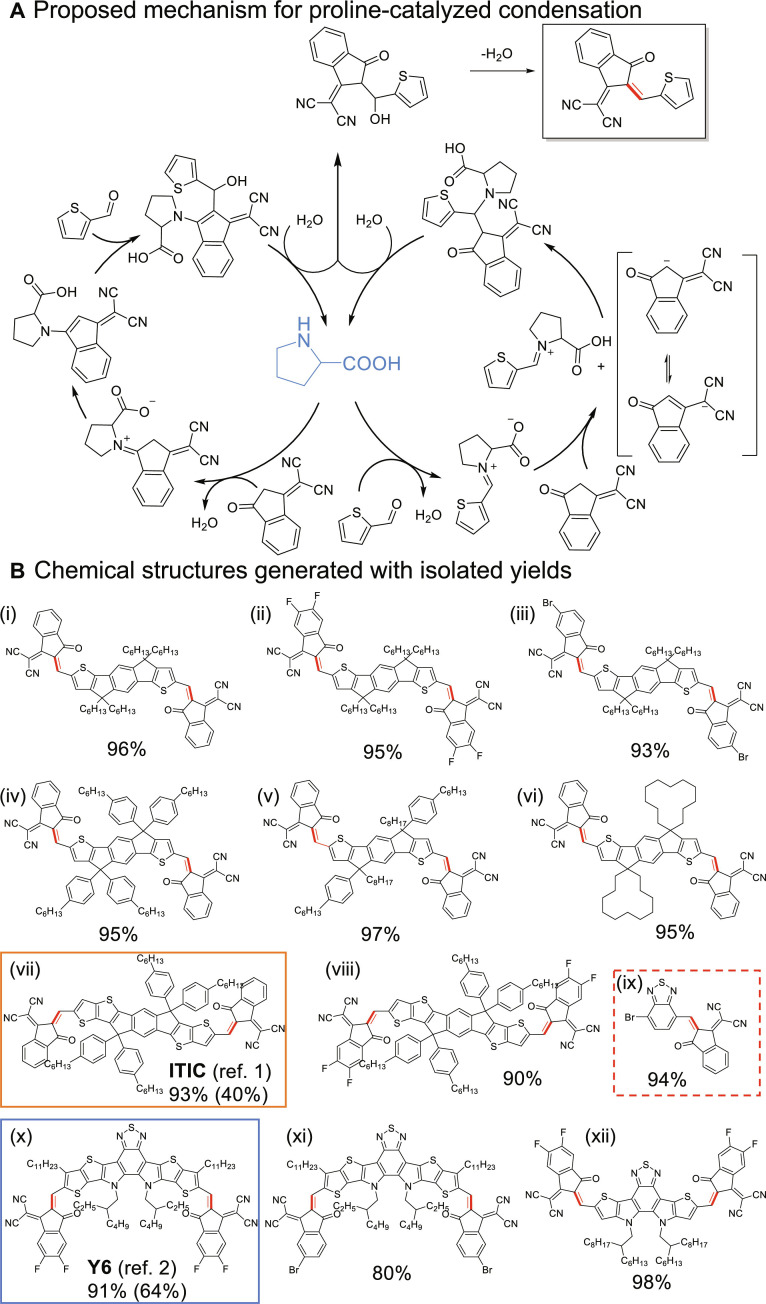
Proline catalyzed condensation between donor and acceptor moieties to form FREAs. (**A**) Proposed mechanism. (**B**) Selected chemical structures with yields using this methodology.

The final step to complete a typical FREA is a (modified) aldol condensation which links the core (D) with the end groups (A) via double bonds to offer the ADA configuration. Typically, pyridine is used as the base for this condensation; however, we have encountered low yield and difficulty in separation. We then focused on the intrinsic features of aldol condensation and chose proline—the first reported organocatalyst for asymmetric aldol addition (*23*)—as our catalyst. We identified a cosolvent system where chloroform was used to dissolve the aldehydes (the core) and ethanol was used to help activate the proline. With this optimized condition, the transformation from aldehydes to the condensed product usually completes in 30 min at room temperature under sonication. For aldehydes having β side chains (e.g., the core of Y6), longer reaction time (~1 hour) was needed, presumably due to steric hindrance. The final ADA FREA can be easily obtained through precipitation in ethanol, followed by centrifugation. A proposed reaction mechanism is outlined in Fig. 4A, where proline was hypothesized to activate both the nucleophile and electrophile, resulting in the observed rapid condensation (*24*). This proline-catalyzed condensation reaction is versatile and robust, applicable to different aromatic aldehydes and end groups commonly seen in FREAs (Fig. 4B). It is worth noting that even in cases involving electron-deficient aldehydes (dashed box in Fig. 4B), the yield of the proline-catalyzed condensation remains excellent. Last, it is worth mentioning that Zhang and co-workers (*25*) recently reported a reaction condition based on BF_3_·OEt_2_/Ac_2_O/toluene for the final step of double bond formation in FREA syntheses, which could notably shorten the reaction time and offer high yield of FREAs as well.

## DISCUSSION

In summary, we have discovered new catalysts and reaction conditions to the syntheses of commonly used FREAs, featuring mild reaction conditions, high yields, and general applicability (a wide range of FREAs). With our new methods, asymmetric or cyclic side chains can now be incorporated into FREAs, broadening the design space of FREAs. With these optimizations where key steps are high yields, the cost for these FREAs can be largely reduced (see the Supplementary Materials for cost analysis); for example, if only considering the cost of reagents, then the total cost for the synthesis of Y6 can be reduced by up to 80%, from $214/mmol (with the reported scheme) to $46/mmol (with our optimized synthetic scheme). Last, given that fuse ring structures have been used broadly in organic semiconductors, our method will have substantialimpact to organic electronics where new materials are the primary driving force.

## MATERIALS AND METHODS

### General consideration

All chemicals were purchased from commercial sources (Sigma-Aldrich, Thermo Fisher Scientific, etc.) and were used as received except when specified. For reactions under argon, the reaction flask was evacuated and refilled with argon three times. Thin-layer chromatography (TLC) was used to monitor reactions. Flash column chromatography (FCC) was performed with specific eluent indicated for each reaction when FCC was used. Eluent used in FCC was half polar to the one in TLC (for example, 10:1 hexane:ethyl acetate in TLC and then 20:1 hexane:ethyl acetate in FCC).

Nuclear magnetic resonance (NMR) measurements were recorded with the Bruker Avance NEO 400 MHz spectrometer, Bruker Avance NEO, 600 MHz spectrometer, and Bruker Avance III 600 MHz spectrometer. Mass spectroscopy samples were analyzed with a Q Exactive HF-X (Thermo Fisher Scientific, Bremen, Germany) mass spectrometer with Atmospheric Pressure Photoionization (APPI)/Atmospheric Pressure Chemical Ionization (APCI) as the ionization source.

### General method for fusing ring via carbon atom

The tertiary alcohol was dissolved (1 mmol) in 10 ml of dichloroethane in a round-bottom flask. Ytterbium triflate (1 mol %) was then added, and the reaction mixture was kept stirring at 80°C. TLC with hexane as the eluent was used to monitor the reaction. When the reaction was completed (usually within 1 hour), the reaction mixture was filtered to recover the catalyst for future use. To obtain the target fused-ring product:

1) When both side chains are hexylphenyl chains:

Passing the filtrate through a short path of silica gel and then removing solvent of the filtrate to obtain the product. No column is necessary (typically).

2) When side chains contain alkyl chains:

After filtration, 3 eq. boron trifluoride etherate was added into the filtrate. The mixture was kept stirring at 80°q. boron trifluoride etherate was added into the filtrate. The mixture was kept stirring at 80alleted, 1 ml ethanol was added to quench the reaction. The mixture was then filtered through a short path of silica gel to remove the boron compounds. The pure product was obtained after solvent removal.

### General method for fusing ring via nitrogen atom

The dinitro compounds (1 mmol), MoO_2_Cl_2_(DMF)_2_ (5 mmol %), and PPh_3_ (5 mmol) were dissolved in toluene (2 ml) in a round-bottom flask. The reaction was heated at 160°C and held at that temperature for 20 min in a CEM Discover 2.0 microwave reactor. For conventional oil bath heating, the reaction was kept stirring at 100°C overnight. The crude product was obtained by precipitating in ethanol and/or was used for the next step without further purification.

### General method for proline-catalyzed aldol condensation between donor and acceptor moieties

The dialdehydes (0.1 mmol), end-group units (0.3 mmol), and proline (0.03 mmol) were mixed in chloroform (5 ml) and 0.5 ml of ethanol. The reaction was typically completed within 1 hour with sonication. Upon solvent removal, a large amount of ethanol was used to wash out the unreacted end-group units and proline to obtain the condensed products as the precipitate. Centrifuge is used to isolate the solid. Silica gel column chromatography with hexane/toluene as eluent could be used to further purify the products.

## Supplementary Materials

### This PDF file includes:

Figs. S1 to S7 Tables S1 and S2 Supplementary Text NMR spectra References
