# Supplementary material for: A general and mild synthetic method for fused-ring electronic acceptors
Source: Sci Adv. 2024 Aug 21;10(34):eadp8150. doi: 10.1126/sciadv.adp8150 (PMC11338226; doi:10.1126/sciadv.adp8150)
Supplement: Supplementary file 1 — Figs. S1 to S7 Tables S1 and S2 Supplementary Text NMR spectra References [file sciadv.adp8150_sm.pdf]

Science Advances
AAAS

Supplementary Materials for

A general and mild synthetic method for fused-ring electronic acceptors

Xiaowei Zhong *et al.*

Corresponding author: Wei You, wyou@unc.edu

Sci. Adv. **10**, eadp8150 (2024)
DOI: 10.1126/sciadv.adp8150

This PDF file includes:

Figs. S1 to S7
Tables S1 and S2
Supplementary Text
NMR spectra
References

A: common 4-hexylphenyl side chains (7)*:*

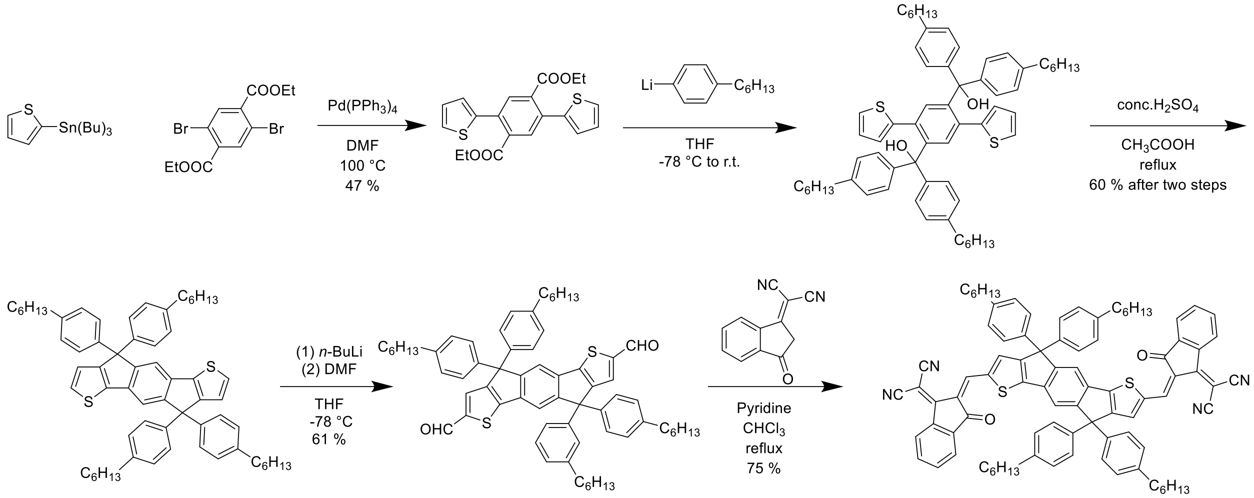

B: two identical alkyl side chains (22)*:*

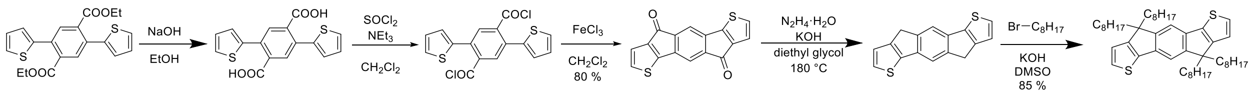

C: side chains of different chemical nature (9)*:*

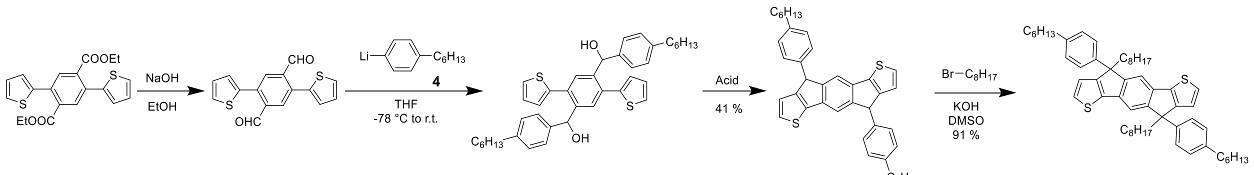

Figure S1. Reported synthetic scheme for general small molecule acceptors with different side chains.

Table S1. Screening conditions for fusing rings

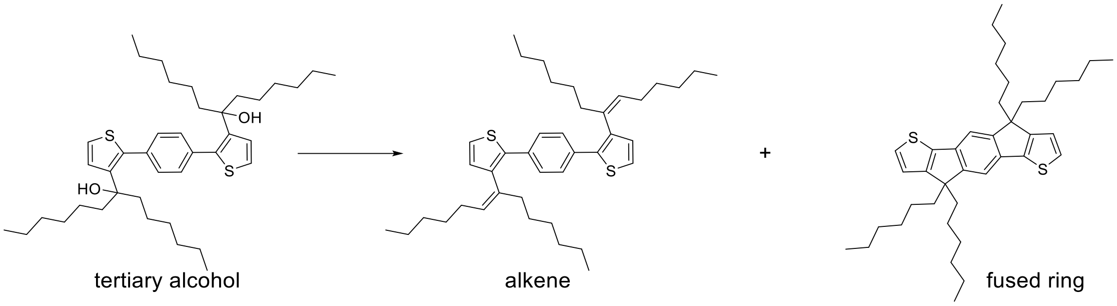

SM	Lewis acid	Solvent	Temperature	Product
Tertiary alcohol	Amberlyst15	Toluene	reflux	Alkenes[a]
Tertiary alcohol	Conc. H_2SO_4	CH_3COOH	r.t.	Alkenes
Tertiary alcohol	TfOH	Methylene chloride	r.t.	Alkenes
Tertiary alcohol	$TiCl_4$	Methylene chloride	r.t	Alkenes
alkene	$BF_3{\cdot}OEt_2$ (2eq)	Dichloroethane	80 °C	Alkenes
Tertiary alcohol	$Yb(OTf)_3$ (0.01eq)	Dichloroethane	r.t. or 80 °C	Alkene[b]
alkene	$BF_3{\cdot}OEt_2$ (3eq)	Dichloroethane	80 °C	Fused ring
Tertiary alcohol	$BF_3{\cdot}OEt_2$ (20eq)	Dichloroethane	80 °C	Fused ring

a. Three types of alkenes with different configurations
b. Only one type of alkene with one configuration

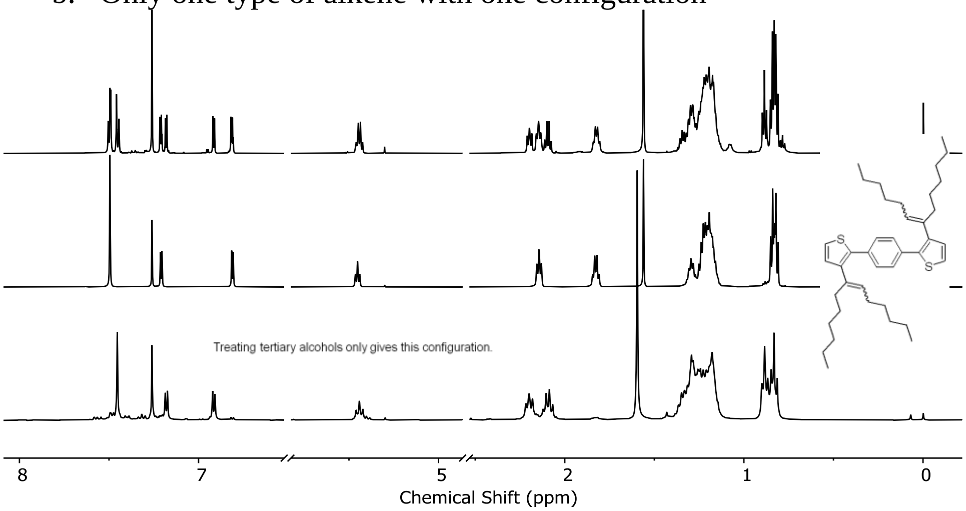

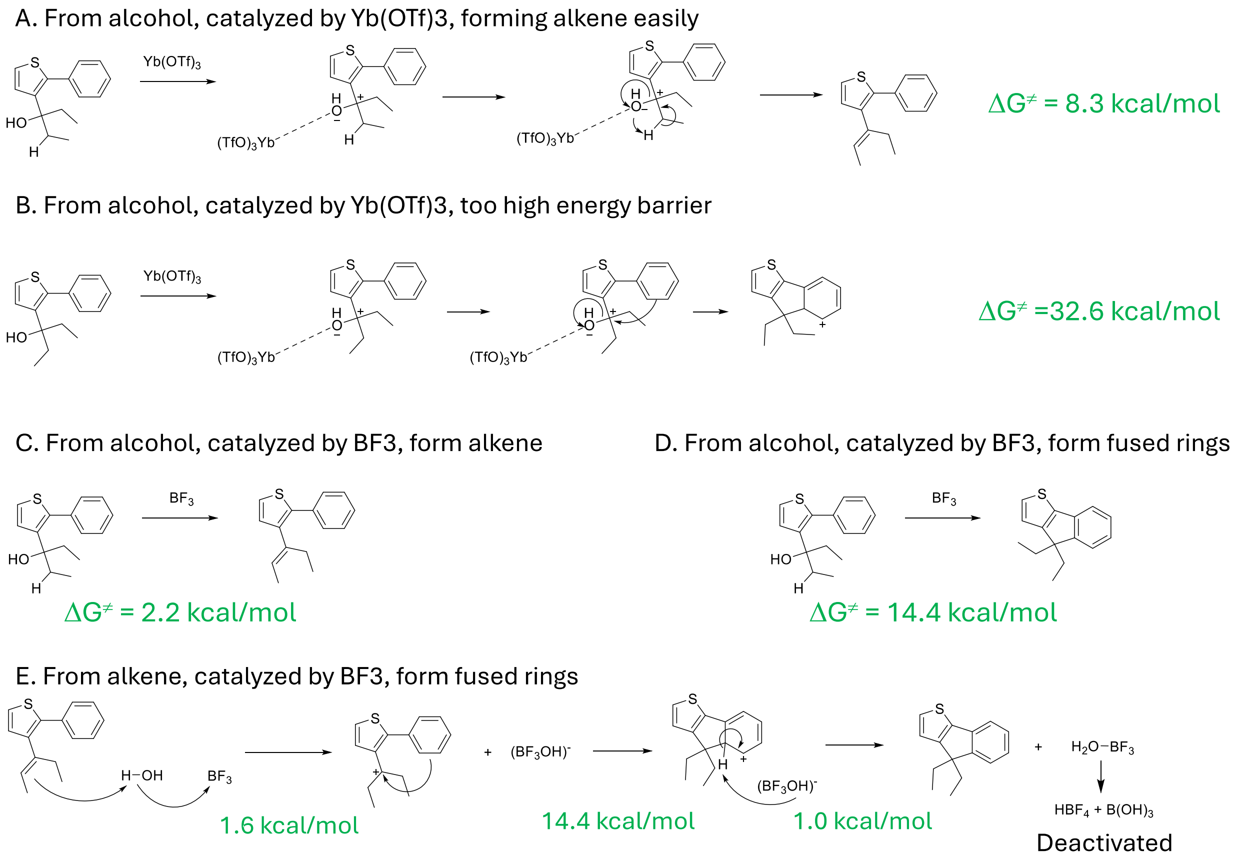

All calculations were performed with Gaussian 16 C01 package (26). Structures were optimized using the DFT M062X exchange-correlation energy density functional (27). The basis set is triple-zeta 6-311+G(d) for S and F elements, 6-311G(d,p) for C and H elements, and CRENBL ECP basis set for Yb (28, 29). Dichloroethane was the solvent included with the CPCM (30) implicit solvent model employed.

Figure S2. Proposed mechanisms for fusing rings having two alkyl side chains in a 2-step process with energy barrier of the transition state.

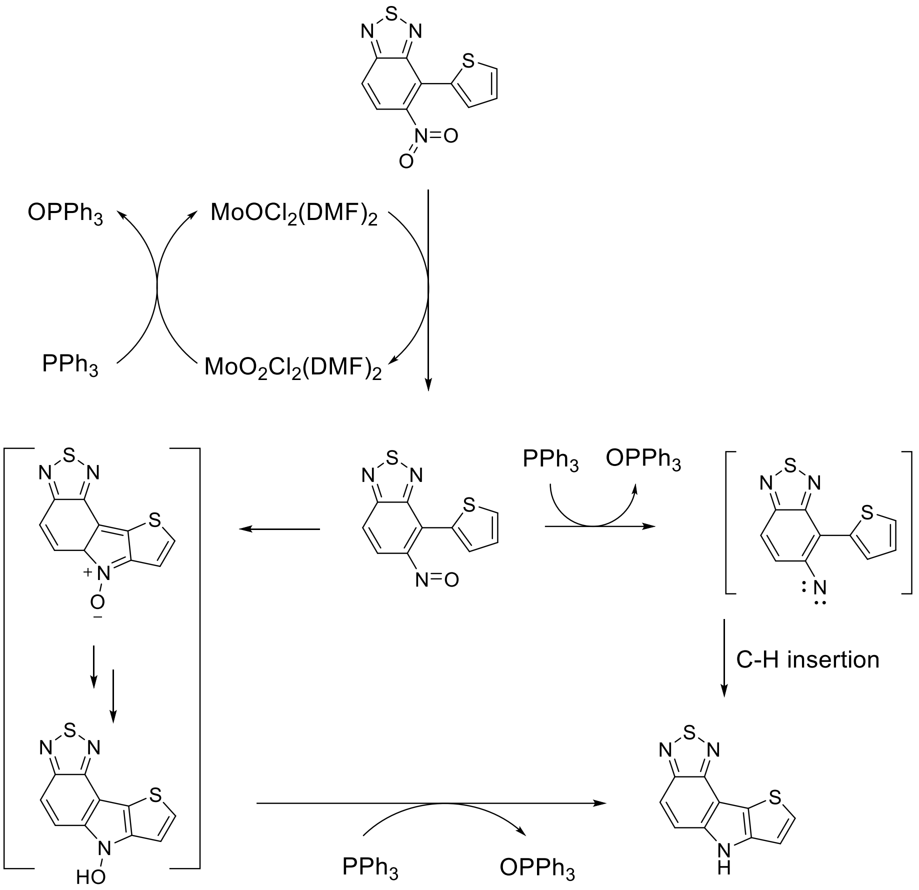

Figure S3. Proposed mechanism for oxo-molybdenum catalyzed Cadogan-Sundberg indole synthesis.

Table S2. Screening conditions for Knoevenagel condensation catalyzed by proline

Solvent	Equivalency of IC unit	Result[a]	TLC
EtOH	3	target product and reactant	Figure S4 (c) below
Chloroform	3	mono-condensed product, and reactant	Figure S4 (d) below
Chloroform/EtOH[b]	3	target product	Figure S4 (b) below
Chloroform/EtOH	2	target product, mono-condensed product, reactant	

a. Monitor the reaction by TLC after 1 h
b. We found that the ratio between chloroform and ethanol was mildly important; chloroform dissolves the reactants and ethanol helps activate the proline and IC unit. The key is to dissolve all the reagents with a mixture of chloroform and ethanol.

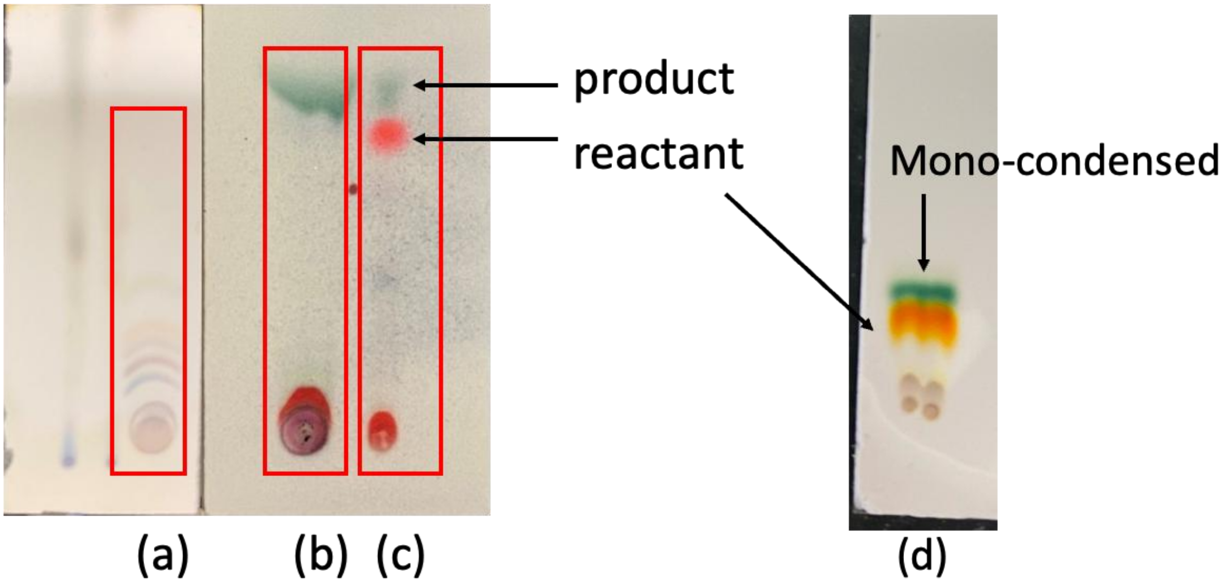

Figure S4. Photos of TLC plates for the condensation reaction under different conditions. (a) pyridine-catalyzed condensation under 50 °C where many impurities can be seen from the TLC; proline-catalyzed condensation at room temperature using (b) ethanol and chloroform as the co-solvent, (c) ethanol-only as the solvent, and (d) chloroform-only as the solvent. Since the dialdehyde is barely soluble in ethanol, the reaction was slow in the ethanol-only solvent as indicated by TLC in (c). On the other hand, ethanol is important for this reaction as the chloroform-only based reaction produced mono-condensed product only (in addition to uncreated starting material, i.e., dialdehyde as the orange spot indicated by TLC in (d)).

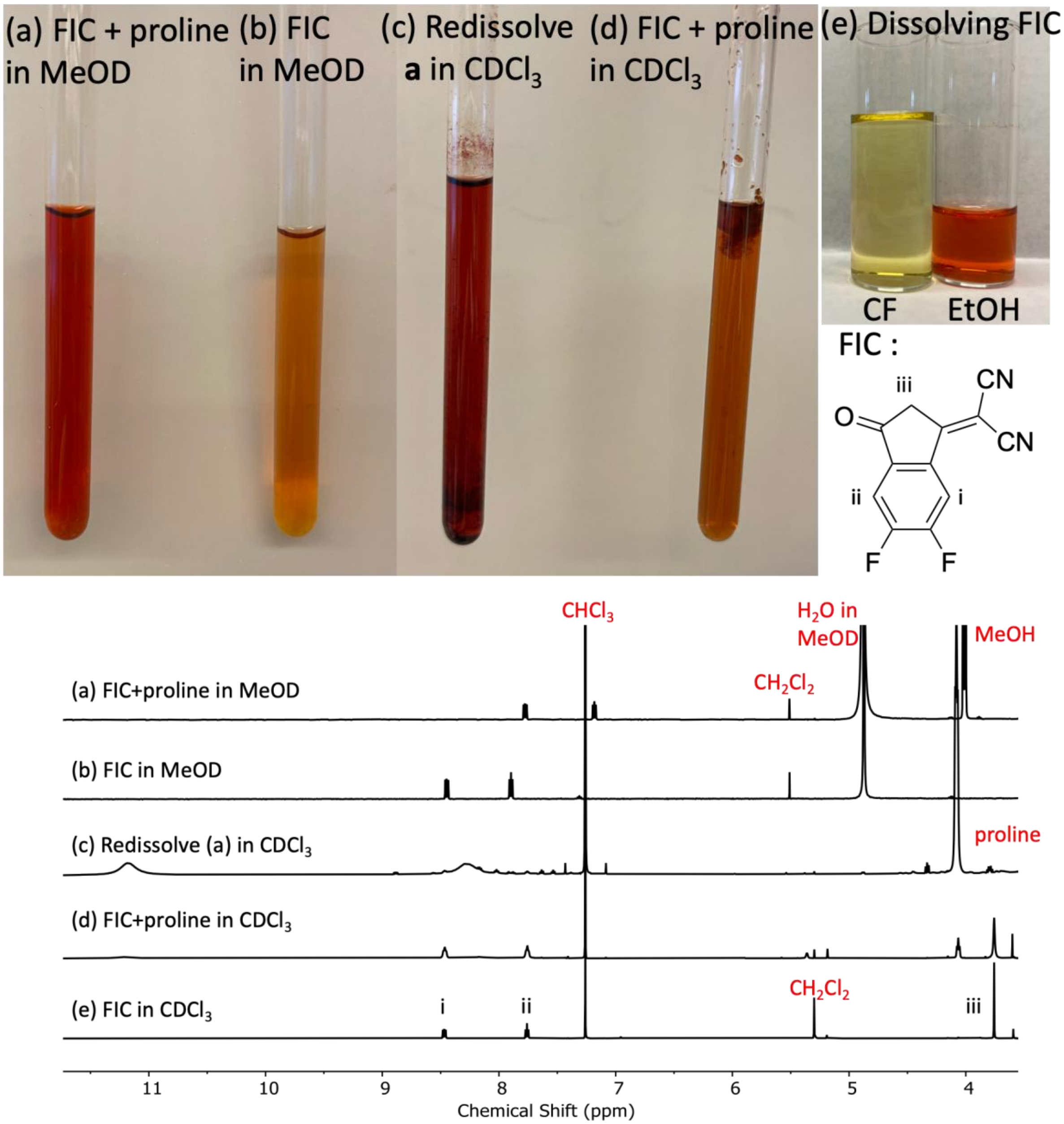

Figure S5. Photos of dissolving IC unit (FIC in this case). (a) FIC and proline (excess) in MeOD showed red color. (b) FIC in MeOD showed orange color. (c) Removing solvent of (a) and redissolve the recovered FIC and proline into $CDCl_3$ color, the color was red as well; however, if FIC and proline were *directly* dissolved in $CDCl_3$, color would be orange, as shown in (d). (e) FIC shows different colors in chloroform and ethanol, respectively.

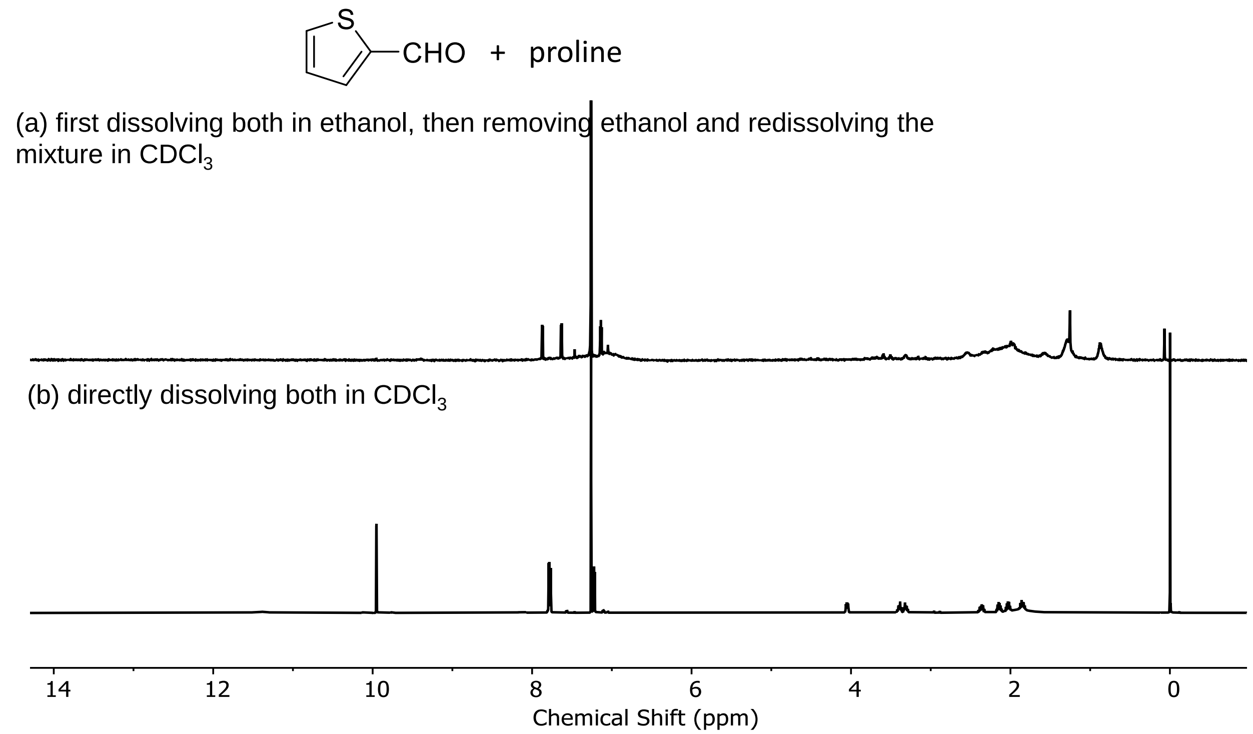

Figure S6. Stacked NMR spectra of treating thiophene-2-carboxyaldehyde with proline in different conditions. (a) These two compounds were mixed in 1 to 1 ratio in ethanol firstly, then the solvent was removed and the obtained mixture was dissolved in deuterated chloroform for NMR measurement. (b) These two compounds were mixed in 1 to 1 ratio in deuterated chloroform directly. When ethanol was used as the solvent, the thiophene-aldehyde apparently interacted with proline as shown in (a) (e.g., the disappearance of the aldehyde peak). By contract, (b) clearly shows the two separated compounds (thiophene-2-carboxyaldehyde and proline).

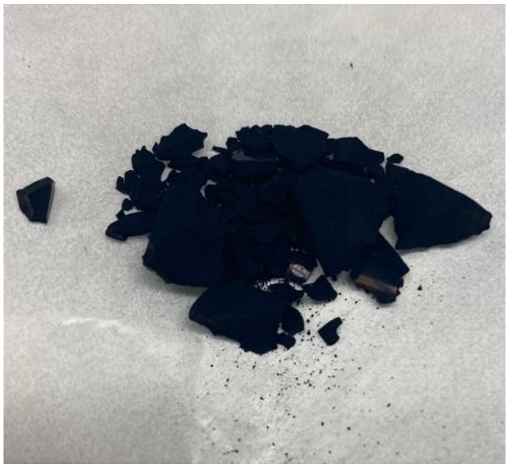

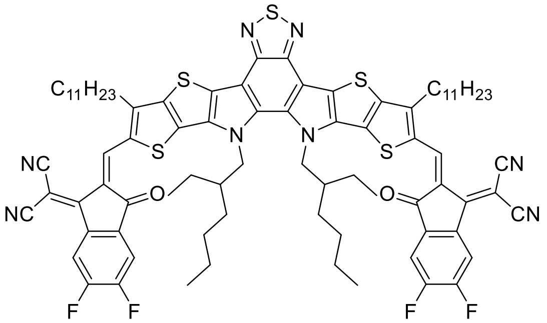

Figure S7. Picture of 1 g Y6 with a total yield of 73% (starting from the dinitro compound), while the reported yield is 38% (2).

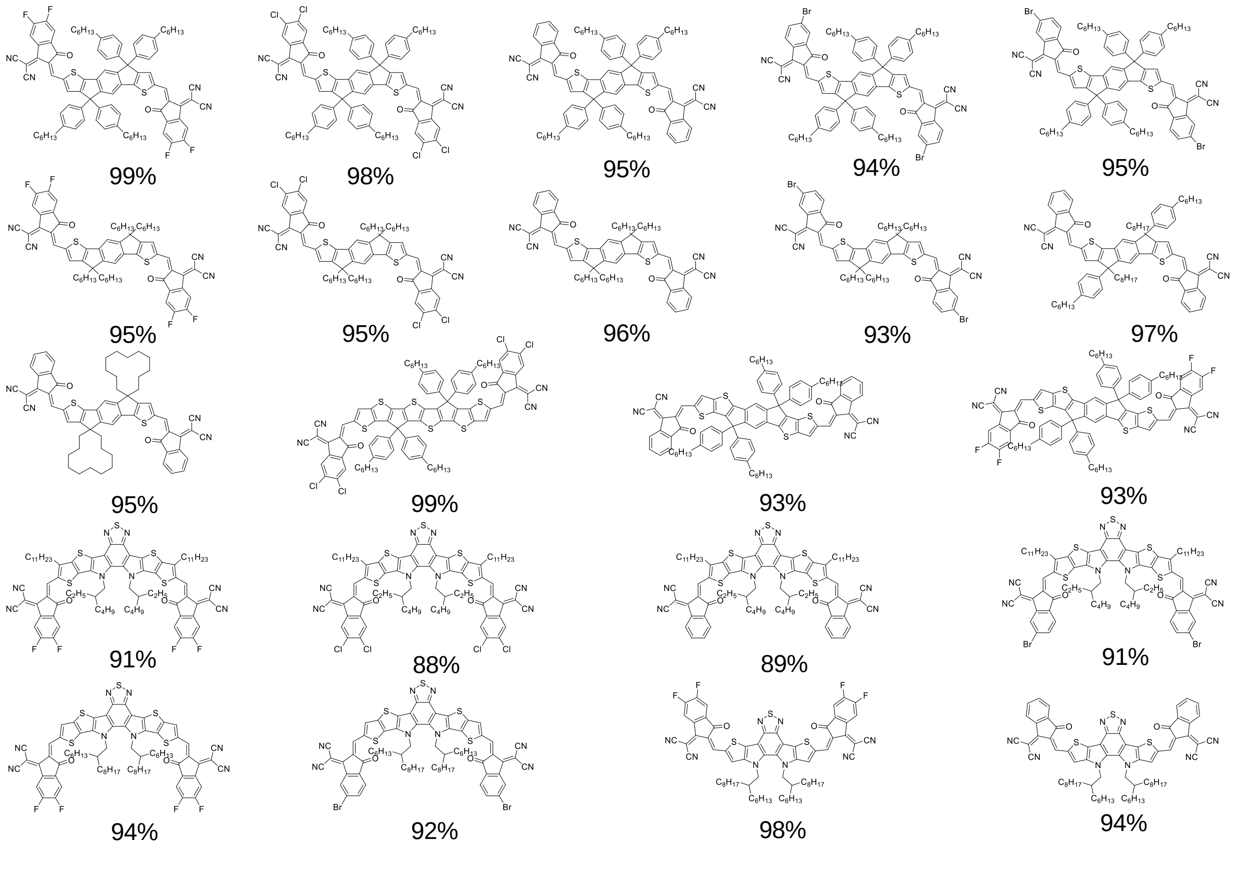

Expanded **Figure 4B** from main text.

Cost Analysis

Y6

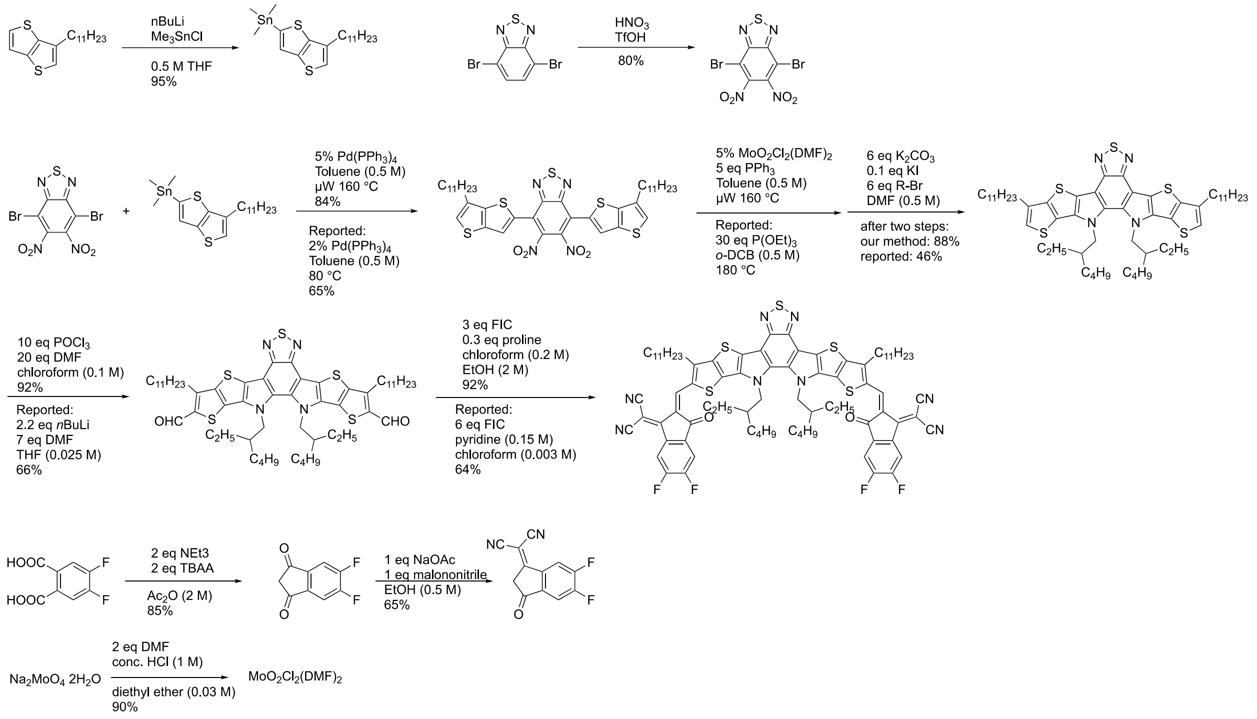

To make **1 mmol** Y6

Chemical	Vendor	Reported ()		Our method	
		mmol or mL	cost	mmol or mL	cost
3-undecylthieno[3,2-*b*]thiophene	Ambeed	17.89	131.75	3.54	26.04
4,7-Dibromo-2,1,3-benzothiadiazole	Ambeed	10.1125	3.66	2	0.72
nBuLi	Sigma	23.1	0.87	3.54	0.13
Me_3SnCl	Sigma	19.68	29.72	3.89	5.87
Fuming HNO_3	Fisher	10 mL	3.34	2 mL	0.66
TfOH	Sigma	10 mL	9.38	2 mL	1.89
$Pd(PPh_3)_2Cl_2$	Sigma	0.16	2.17		
$Pd(PPh_3)_4$	Sigma			0.08	1.3
PPh_3	Sigma			6.71	0.16
$P(OEt)_3$	Sigma	153.40	1.32		
K_2CO_3	Sigma	15.78	0.14	8.06	0.07
KI	Sigma	0..53	0.12	0.13	0.03
2-Ethylhexylbromide	Ambeed	15.78	0.11	8.06	0.06
$POCl_3$	Sigma			11.81	0.19
L-proline	Sigma			0.33	0.03
4,5-Difluorophthalic acid	Ambeed	16.98	22.89	5.9	7.95

NEt_3	Sigma	33.95mmol	0.5	11.8 mmol	0.09
Tert-butyl acetoacetate (TBAA)	Sigma	33.95	0.26	11.8 mmol	0.17
NaOAc	Sigma	16.98	0.15	5.9	0.05
Malononitrile	Sigma	16.98	0.34	5.9	0.12
THF	Fisher	131 mL	1.36	4	0.04
Toluene	Fisher	16 mL	0.08	6mL	0.03
o-DCB	Fisher	11mL	0.38		
DMF	Sigma	43.28 mL	1.57	4.83 mL	0.17
Chloroform	Fisher	450mL	3.72	17mL	0.13
Pyridine	Sigma	10 mL	0.01		
EtOH	Fisher	34 mL	0.17	1	0.01
Ac_2O	Sigma	8 mL	0.1		
$MoO_2Cl_2(DMF)_2$*				0.07	0.03
**In total**			214.11		45.94

ITIC

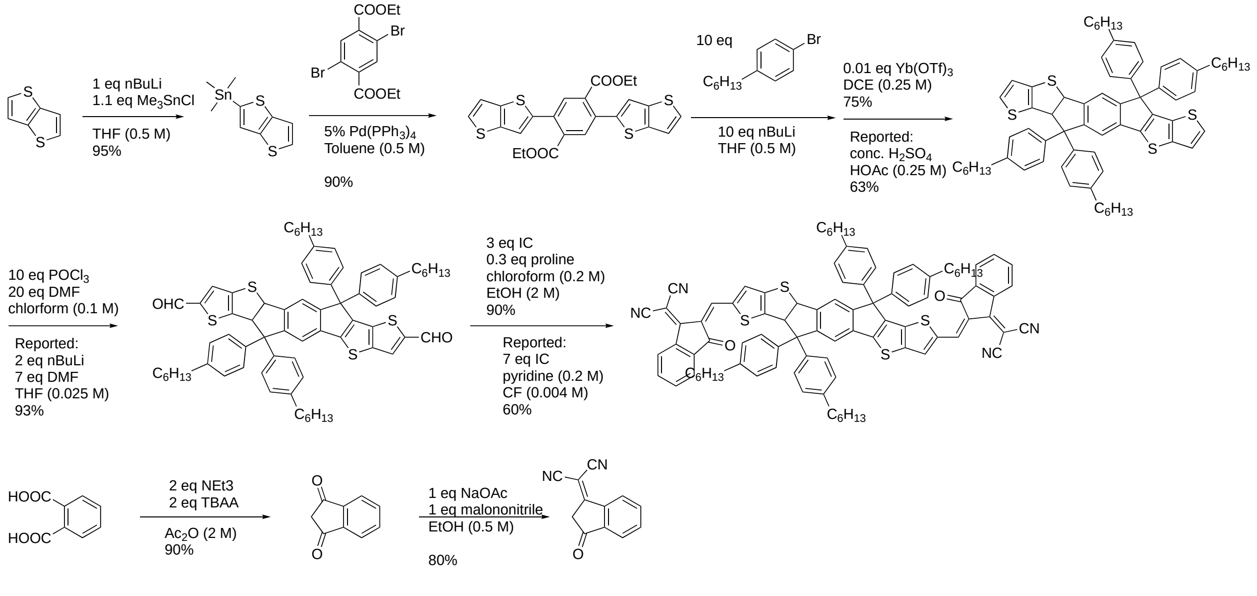

To make **1 mmol** ITIC

Chemical	Vendor	Reported		Our method	
		mmol or mL	cost	mmol or mL	cost
thieno[3,2-*b*]thiophene	Ambeed	9.38	5.01	3.92	2.09
Diethyl 2,5-dibromoterephthalate	Ambeed	4.24	1.64	1.77	0.69
nBuLi	Sigma	40.89	1.54	19.85	0.75
Me_3SnCl	Sigma	10.32	15.58	4.31	6.5
$Pd(PPh_3)_4$	Sigma	0.21	3.45	0.09	1.44
1-Bromo-4-hexylbenzene	Ambeed			15.93	5.89

Sulfuric Acid	Sigma	1mL	0.02		
HOAc	Sigma	11mL	0.26		
$Yb(OTf)_3$				0.02	0.19
phthalic acid	Ambeed	16.21	0.06	4.63	0.02
NEt_3	Sigma	32.42	0.25	9.25	0.07
Tert-butyl acetoacetate (TBAA)	Sigma	32.42	0.48	9.25	0.14
NaOAc	Sigma	16.21	0.15	4.63	0.04
Malononitrile	Sigma	16.21	0.32	4.63	0.09
THF	Fisher	97 mL	1.01	11 mL	0.11
Toluene	Fisher	8 mL	0.04	4 mL	0.02
Dichloroethane	Sigma			6 mL	0.22
$POCl_3$				11.95	0.19
DMF	Sigma	0.97 mL	0.03	1.85mL	0.07
Chloroform	Fisher	417	3.31	18mL	0.13
L-proline				0.33	0.03
Pyridine	Sigma	11	0.01		
EtOH	Fisher	32 mL	0.16	1mL	
Ac_2O	Sigma	0.008	0.09		
**In total**			33.41		18.68

Purification comparison

	Reported	Our method
Coupling	Column	recrystallization
Friedel-Crafts alkylation	Column	Short-path silica gel filtration
Indole synthesis	Column	Column
Formylation	Column	Short-path silica gel filtration
Aldol condensation	Column	recrystallization

Detailed synthetic procedure for selected compounds

1,4-bis(3-bromothiophen-2-yl)benzene

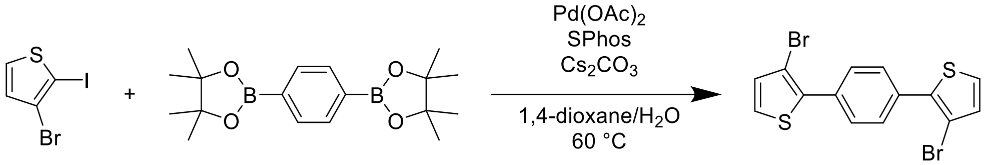

A mixture of 3-bromo-2-iodothiophene (2.95 g, 10.2 mmol, 2.04 eq), 1,4-bis(4,4,5,5-tetramethyl-1,3,2-dioxaborolan-2-yl)benzene (1.65 g, 5.0 mmol, 1 eq), palladium acetate (55 mg, 5 % mmol), SPhos (205 mg, 10 % mmol) and cesium carbonate (9.97 g, 30.6 mmol, 6.3 eq) were mixed in a RB flask, which was degassed via vacuum and refilled with argon three times. Subsequently, 30 mL of degassed 1,4-dioxane and 10 mL degassed water were added to the mixture. Then the mixture was stirred at 60 °C overnight. The reaction was quenched by 100 mL water and extracted by chloroform (100 mL in total in three times). The organic phase was dried over magnesium sulfate. Dry loading the mixture on silica gel and hexane was used for column chromatography to give the product as white solid. (1.63 g, 81 %).

^{1}H NMR (400 MHz $CDCl_3$ ppm): 7.74 (s, 4H), 7.31 (dd, J = 5.4, 1.9 Hz, 2H), 7.08 (dd, J = 5.4, 1.9 Hz, 2H).
^{13}C NMR (100 MHz, $CDCl_3$) δ: 137.54, 132.69, 131.94, 129.03, 125.34, 107.84.
HRMS([m]·) m/z calcd. for C14H8Br2S2 397.84342, found 397.84250.

diethyl 2,2'-(1,4-phenylene)bis(thiophene-3-carboxylate)

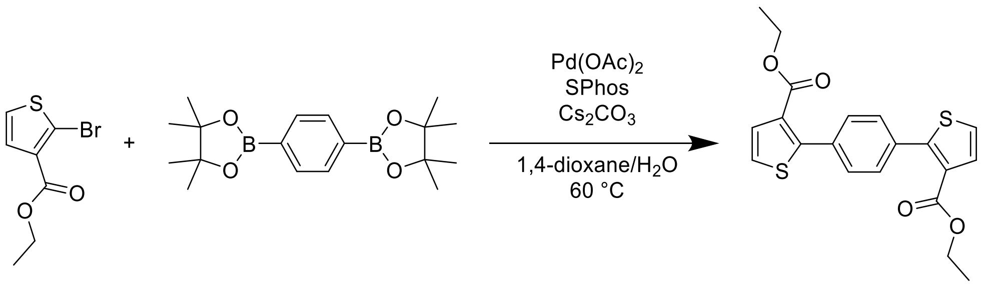

A mixture of ethyl 2-bromothiophene-3-carboxylate (2.40 g, 10.2 mmol, 2.04 eq), 1,4-bis(4,4,5,5-tetramethyl-1,3,2-dioxaborolan-2-yl)benzene (1.65 g, 5.0 mmol, 1 eq), palladium acetate (55 mg, 5 % mmol), SPhos (205 mg, 10 % mmol) and cesium carbonate (9.97 g, 30.6 mmol, 6.3 eq) were mixed in a RB flask, which was degassed via vacuum and refilled with argon three times. Subsequently, 30 mL degassed 1,4-dioxane and 10 mL degassed water were added to the mixture. Then the mixture was stirred at 60 °C overnight. The reaction was quenched by 100 mL water and extracted by chloroform (100 mL in total in three times). The organic phase was dried over magnesium sulfate. Dry loading the mixture on silica gel and hexane was used for column chromatography to give the product as white solid. (1.98 g, 93 %).

^{1}H NMR (400 MHz $CDCl_3$ ppm): 7.55-7.50 (m, 6H), 7.26 (d, J=5.2 Hz, 2H), 4.23 (q, 4H), 1.22 (t, 6H).

diethyl 2,2'-(1,4-phenylene)bis(thiophene-3-carboxylate)

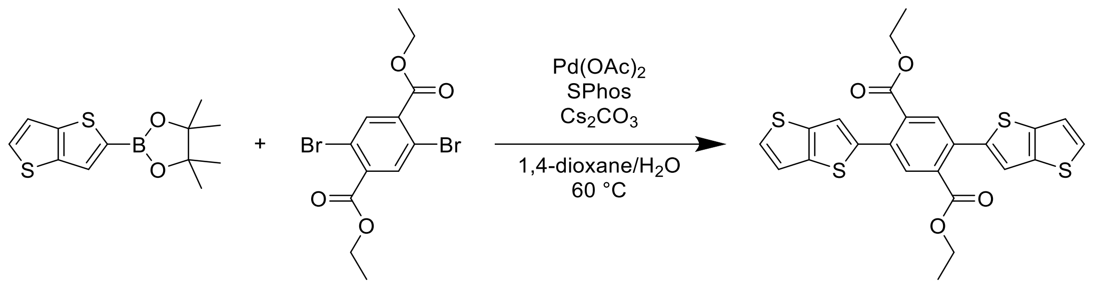

A mixture of ethyl 4,4,5,5-tetramethyl-2-(thieno[3,2-b]thiophen-2-yl)-1,3,2-dioxaborolane (640 mg, 2.4 mmol, 2.1 eq), diethyl 2,5-dibromoterephthalate (380 mg, 1 mmol, 1 eq), palladium acetate (11 mg, 5 % mmol), SPhos (41 mg, 10 % mmol) and cesium carbonate (2.0 g, 30.6 mmol, 6.3 eq) were mixed in a RB flask, which was degassed via vacuum and refilled with argon three times. Later, 10 mL degassed 1,4-dioxane and 2 mL degassed water were added to the mixture. Then the mixture was stirred at 60 ℃ overnight. The reaction was quenched by 50 mL water and extracted by chloroform (30 mL in total in three times). The organic phase was dried over magnesium sulfate. Dry loading the mixture on silica gel and hexane was used for column chromatography to give the product as white solid. (440 mg, 88 %).

^{1}H NMR (600 MHz, $CDCl_3$) δ 7.89 (s, 2H), 7.40 (d, *J* = 5.2 Hz, 2H), 7.30 – 7.26 (m, 4H), 4.25 (q, 4H), 1.13 (t, *J* = 7.1 Hz, 6H).

diethyl 2,2'-(1,4-phenylene)bis(thiophene-3-carboxylate)

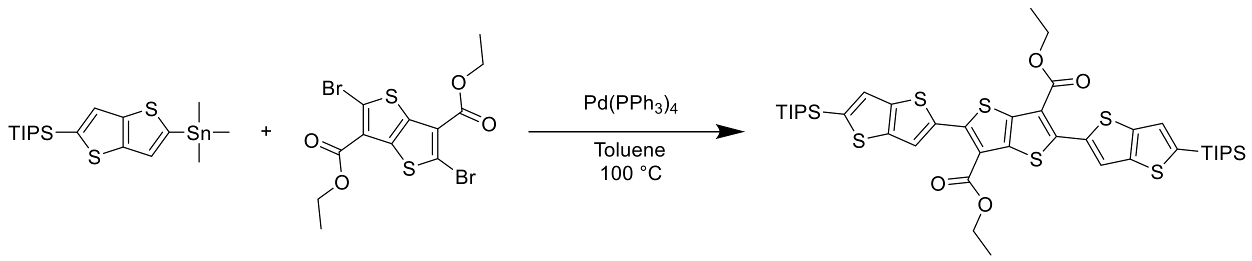

A mixture of triisopropyl(5-(trimethylstannyl)thieno[3,2-b]thiophen-2-yl)silane (640 mg, 2.4 mmol, 2.1 eq), diethyl 2,5-dibromothieno[3,2-b]thiophene-3,6-dicarboxylate (380 mg, 1 mmol, 1 eq), palladium tetrakis(triphenylphosphine (11 mg, 5 % mmol) were mixed in a RB flask, which was degassed via vacuum and refilled with argon three times. Subsequently, 10 mL degassed toluene was added to the mixture. Then the mixture was stirred at 100 ℃ overnight. The reaction was quenched by 100 mL water and extracted by chloroform (30 mL in total in three times). The organic phase was dried over magnesium sulfate. Dry loading the mixture on silica gel and hexane was used for column chromatography to give the product as white solid. (440 mg, 88 %).

^{1}H NMR (400 MHz $CDCl_3$ ppm): 7.82 (s, 2H), 7.37 (s, 2H), 4.43 (q, 4H), 1.41 (t+sept, 12H), 1.13 (d, 36H).

tertiary alcohols

From double nuclephilic attack to the ester with aryl lithium:

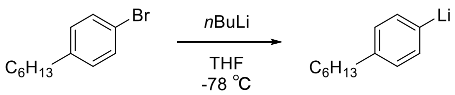

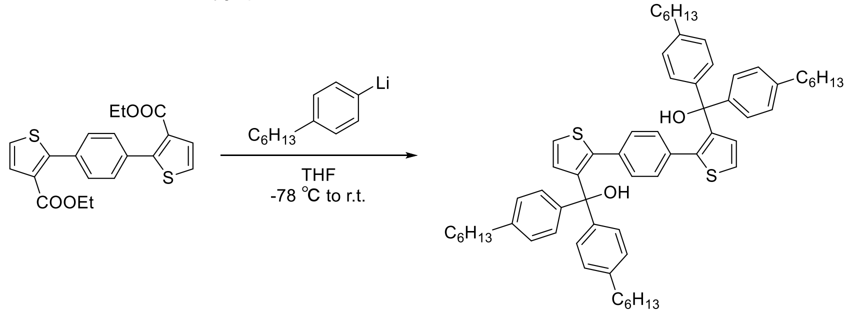

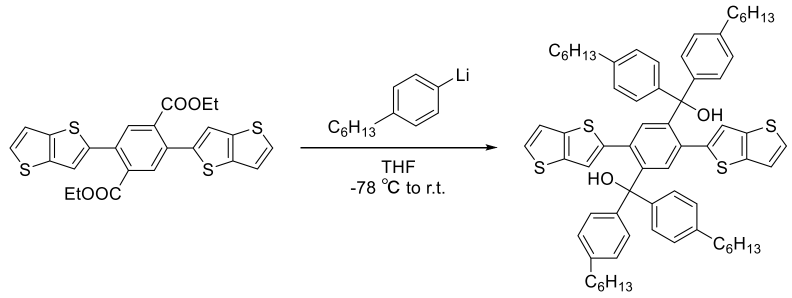

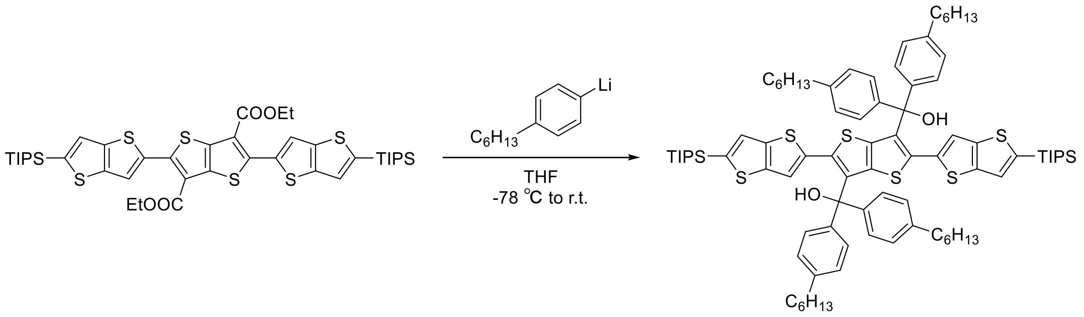

From double nuclephilic attack to the ester with Grignard reagents:

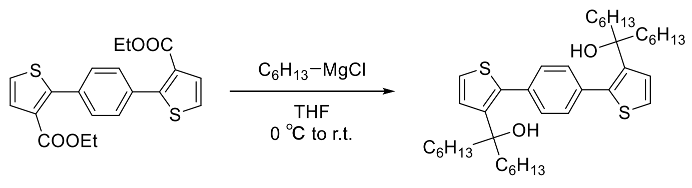

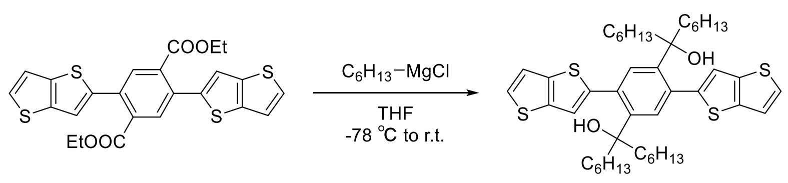

From nucleophilic attack of the core (Grignard) to ketone to synthesize tertiary ketone in one step

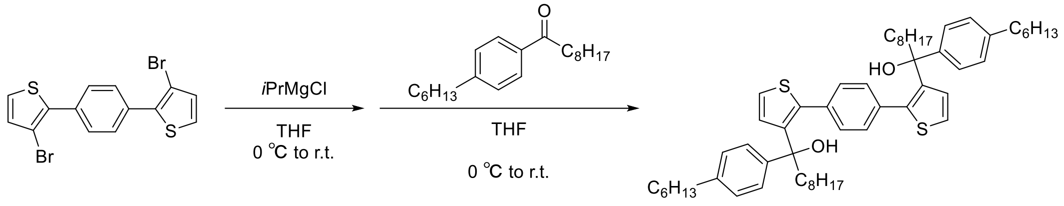

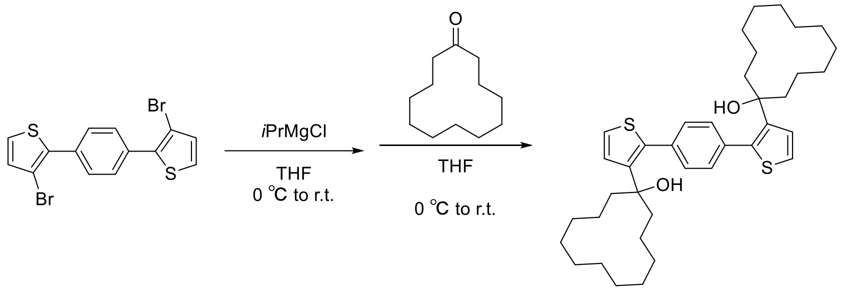

Representative synthetic procedure

Under argon atmosphere, 1,4-bis(3-bromothiophen-2-yl)benzene (400 mg, 1 mmol) was dissolved in 3 mL THF in a RB flask. Then isopropylmagnesium chloride (2M THF solution, 3 mmol) was added dropwise under 0 °C. The reaction mixture was kept stirring at room temperature for 3 hours, before the specific ketone (4 mmol) was added at 0 °C. Then the reaction was heated at 50 °C overnight. The reaction mixture was quenched by saturated ammonia chloride solution and the products were extracted by ethyl acetate. The organic phase was dried over magnesium sulfate. For some reactions, dry loading the mixture on silica gel and hexane/EtOAc (20/1 to 10/1) was used for column chromatography to give the product as white solid. The (crude) product is used immediately in next step.

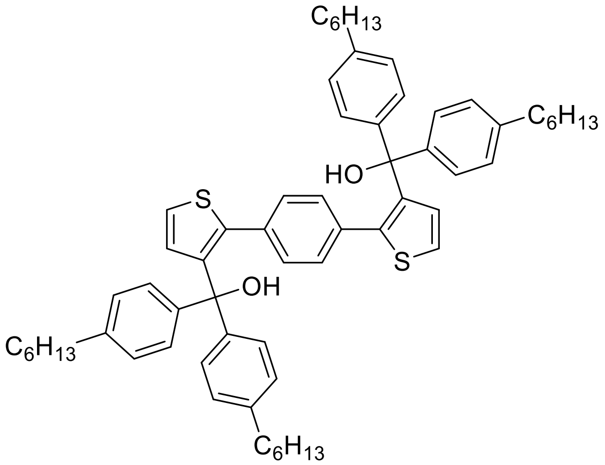

^{1}H NMR (400 MHz $CDCl_3$ ppm): 7.13 – 7.03 (m, 18H), 6.90 (s, 4H), 6.52 (d, J = 5.3 Hz, 2H), 2.61 – 2.54 (t, 8H), 1.59 (m, 8H), 1.25 (m, 24 H), 0.88 (m, 12H).

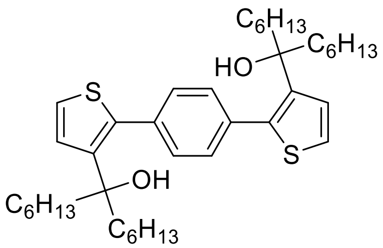

^{1}H NMR (400 MHz $CDCl_3$ ppm): 7.42 (s, 4H), 7.25 (d, *J* = 5.3 Hz, 2H), 7.01 (d, *J* = 5.3 Hz, 2H), 1.69 (m, 8H), 1.25 (m, 32 H), 0.88 (t, 12H).

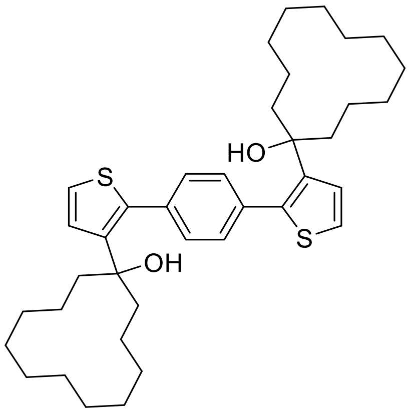

^{1}H NMR (400 MHz $CDCl_3$ ppm): 7.47 (s, 4H), 7.21 (d, *J* = 5.3 Hz, 2H), 7.06 (d, *J* = 5.3 Hz, 2H), 1.84 (m, 4H), 1.77 (m, 4H), 1.33 (m, 36H).

Fused rings via carbon atoms

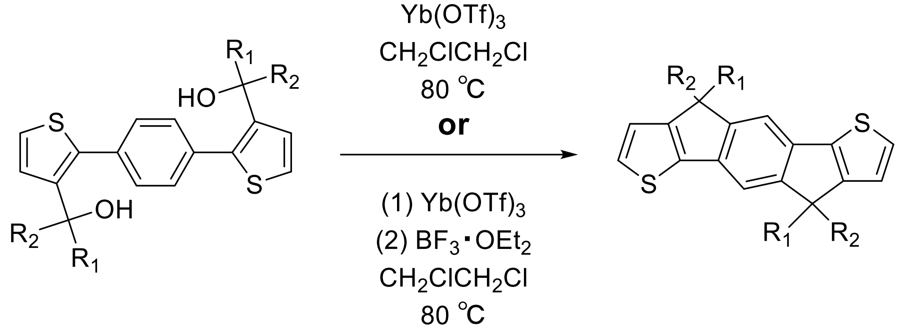

<u>Representative synthetic procedure</u>

The tertiary alcohol was dissolved (1 mmol) in 10 mL dichloroethane in a RB flask. 1 mol% of ytterbium triflate was then added, and the reaction mixture was kept stirring at 80 °C. TLC with hexane as the eluent was used to monitor the reaction. When the reaction was completed (usually within 1h), the reaction mixture was filtered to recover the catalyst for future use. To obtain the target fused ring product:

(1) <u>When both side chains are hexylphenyl chains:</u>
Passing the filtrate through a short path of silica gel, and then removing solvent of the filtrate to obtain the product. No column is necessary (typically).

(2) <u>When side chains contain alkyl chains</u>
After filtration, 3 eq. boron trifluoride etherate was added into the filtrate. The mixture was kept stirring at 80 ℃ and its progress was monitored with TLC using hexane as the eluent. When the reaction was completed, 1mL ethanol was added to quench the reaction. The mixture was then filtered through a short path of silica gel to remove the boron compounds. The pure product was obtained after solvent removal.

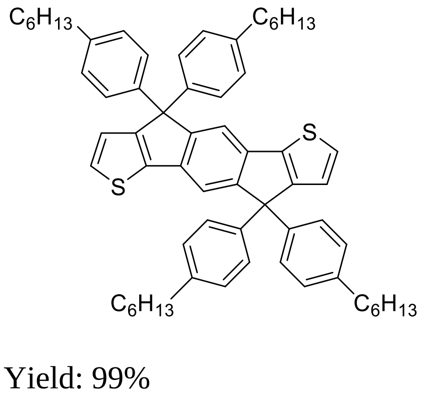

Yield: 99%
^{1}H NMR (400 MHz $CDCl_3$ ppm): 7.43 (s, 2H), 7.24 (d, J = 4.9 Hz, 2H), 7.15 (d, J = 8.3 Hz, 8H), 7.05 (d, J = 8.3 Hz, 8H), 7.00 (d, J = 4.9 Hz, 2H), 2.58 – 2.51 (m, 8H), 1.61 – 1.56 (m, 8H), 1.37 – 1.23 (m, 24H), 0.86 (d, J = 6.7 Hz, 12H).Agreed with previously reported (7).

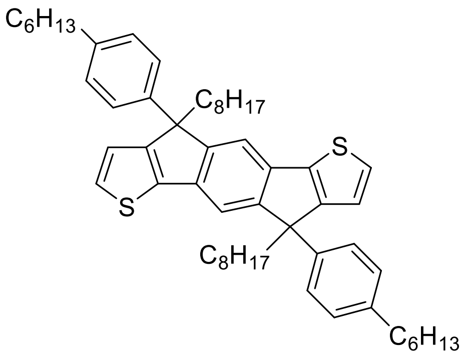

Yield: 92% after two steps

^{1}H NMR (400 MHz $CDCl_3$ ppm): 7.20-7.03 (m, 12H), 6.66 (s, 2H), 2.54 (t, 4H), 2.38 (m, 2H), 2.15 (m, 2H), 2.31 (m, 40H), 0.87 (t, 12H).

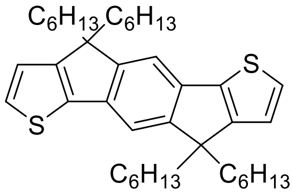

Yield: 95% after two steps

^{1}H NMR (400 MHz $CDCl_3$ ppm): 7.26 (m, 4H), 6.96 (d, 2H), 1.95 (m, 4H), 1.85 (m, 4H), 1.20-0.96 (m, 32H), 0.77 (t, 12H). Agreed with previously reported (31).
^{1}H NMR (500 MHz CD_2Cl_2 ppm): 7.31 (s, 2H), 7.26 (d, *J* = 4.8 Hz, 2H), 6.97 (d, *J* = 4.8 Hz, 2H), 2.02 – 1.92 (m, 4H), 1.92 – 1.80 (m, 4H), 1.07 (m, 24H), 0.75 (m 20H).

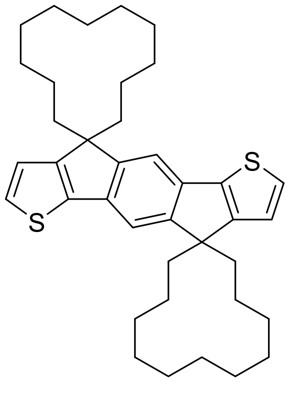

Yield: 96% after two steps

^{1}H NMR (400 MHz $CDCl_3$ ppm): 7.43 (s, 2H), 7.22 (d, *J* = 4.9 Hz, 2H), 7.06 (d, *J* = 4.9 Hz, 2H), 1.75 (m, 4H), 1.69 (m, 4H), 1.51-1.25 (m, 36H).

^{13}C NMR (100 MHz, $CDCl_3$) δ: 157.12, 154.36, 140.57, 133.80, 125.74, 122.97, 115.31, 52.00, 32.01, 29.75, 26.97, 23.04, 22.45, 21.49

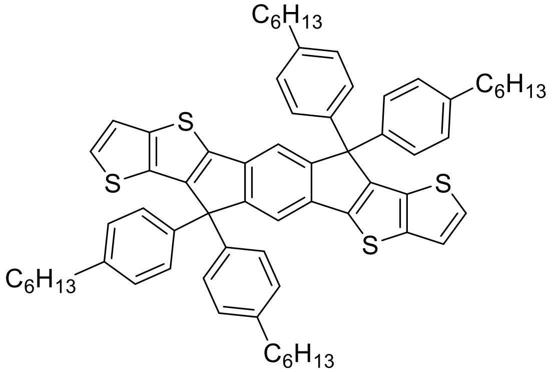

Yield: 98%

^1H NMR (600 MHz $CDCl_3$ ppm): 7.51 (s, 2H), 7.39 (dd, *J* = 5.2 Hz, 2H), 7.25 (dd, *J* = 5.2 Hz, 2H), 7.21 (d, *J* = 8.3 Hz, 8H), 7.10 (d, *J* = 8.3 Hz, 8H), 2.58 (t, 8H), 1.67 (m, 8H), 1.2 (m, 24H), 0.88 (t, 12H).

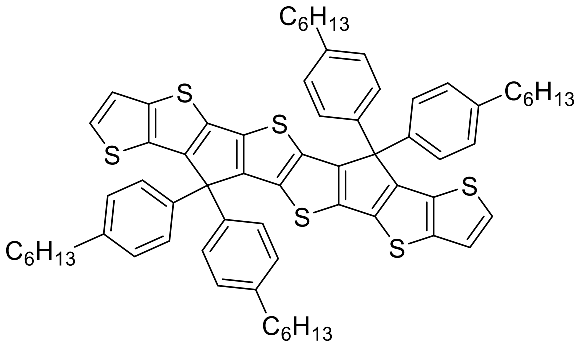

Yield: 98%

^1H NMR (400 MHz, $CDCl_3$) δ 7.26 (d, *J* = 5.2 Hz, 2H), 7.23 (d, *J* = 5.2 Hz, 2H), 7.16 (d, *J* = 8.3 Hz, 8H), 7.10 (d, *J* = 8.3 Hz, 8H), 2.54 (dd, 8H), 1.59 (d, 8H), 1.38 – 1.19 (m, 24H), 0.85 (m, 12H).

Synthesis of Y6 series:

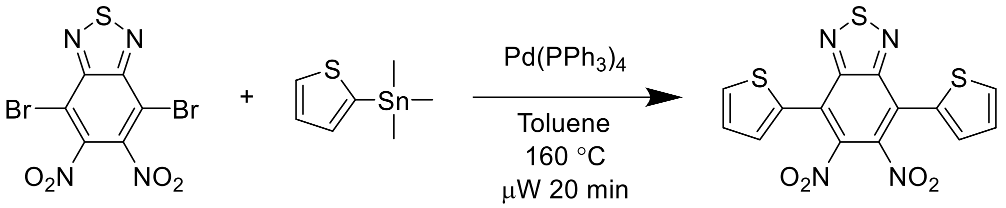

A mixture of 4,7-dibromo-5,6-dinitrobenzo[c][1,2,5]thiadiazole (382 mg, 1 mmol, 1 eq), trimethyl(thiophen-2-yl)stannane (550 mg, 2.1 mmol, 2.1 eq), palladium tetrakis(triphenylphosphine (11 mg, 5 % mmol) were mixed in a microwave tube with 10 mL toluene in glovebox. Then the mixture was stirred at 160 ℃ 20 min with CEM discover 2.0 microwave synthesizer. The reaction was quenched by 100 mL water and extracted by chloroform (30 mL in total in three times). The organic phase was dried over magnesium sulfate. Dry loading the mixture on silica gel and hexane was used for column chromatography to give the product as orange solid. (355 mg, 91 %).

^{1}H NMR (600 MHz $CDCl_3$ ppm): 7.74 (dd, *J* = 5.1, 1.2 Hz, 2H), 7.52 (dd, *J* = 3.7, 1.2 Hz, 2H), 7.24 (dd, *J* = 5.1, 3.7 Hz, 2H). Agreed with previously reported (13).

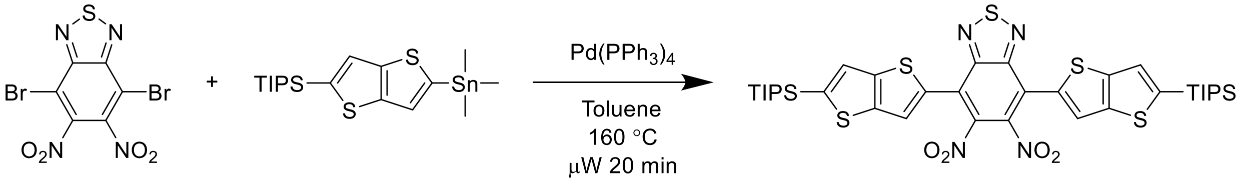

Followed the previous coupling procedure. Yield: 92%

^{1}H NMR (600 MHz $CDCl_3$ ppm): 7.71 (s, 2H), 7.43 (d, 2H), 1.43 (t, 6H), 1.14 (d, 36H). Agreed with previously reported (32).

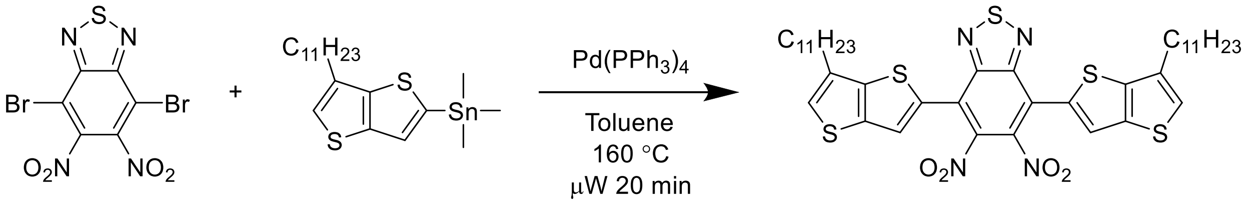

Followed the previous coupling procedure. Yield: 84%

^{1}H NMR (600 MHz $CDCl_3$ ppm): 7.71 (s, 2H), 7.18 (s, 2H), 2.78 (t, 4H), 1.79 (m, 4H), 1.45-1.18 (m, 32H), 0.88 (t, 6H). Agreed with previously reported (2).

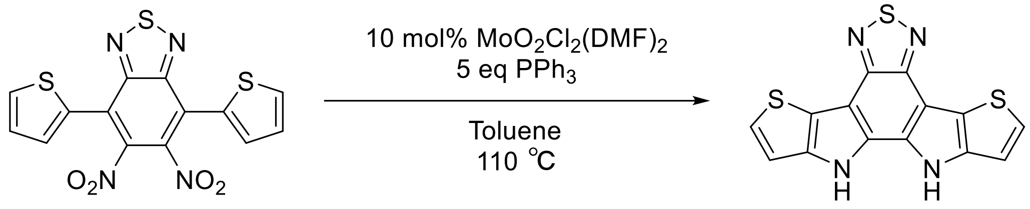

Preparation of $MoO_2Cl_2(DMF)_2$

$Na_2MoO_4 \cdot 2H_2O$ (4.84 g, 20 mmol) was dissolved in H_2O (20 mL) in a RB flask and concentrated HCl (20 mL) was then added. 30 min later, ethyl ether (3 × 10 mL) was used for extraction. The combined organic phase was dried over Na_2SO_4 and filtered. To the filtrate, DMF (3 mL) was added. 5 min later, the precipitates were filtered and washed by ethyl ether. The catalyst was used as obtained without further purification and characterization. It was stored under nitrogen for future use. Yield: 90%.

Indole synthesis

The above nitro compounds (1 mmol), $MoO_2Cl_2(DMF)_2$ (5% mmol) and PPh_3 (5 mmol) were dissolved in toluene (2 mL) in a RB flask. The reaction was heated at 160 °C and held at that temperature for 20 min in a CEM Discover 2.0 microwave reactor. For conventional oil-bath heating, the reaction was kept stirring at 100 °C overnight. The crude product was used for the next step without further purification. For stable compounds, the reaction could be precipitated into ethanol and the crude product was obtained by filtration as a solid. Otherwise, the product was subjected to next alkylation without solvent removal.

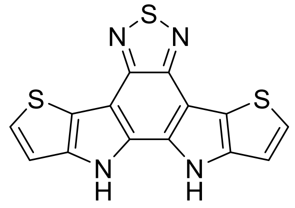

Yield: 92%

^1H NMR (400 MHz DMSO-*d*6 ppm): 11.90 (s, 2H), 7.62 (d, *J* = 5.1 Hz, 2H), 7.44 (d, *J* = 5.1 Hz, 2H).

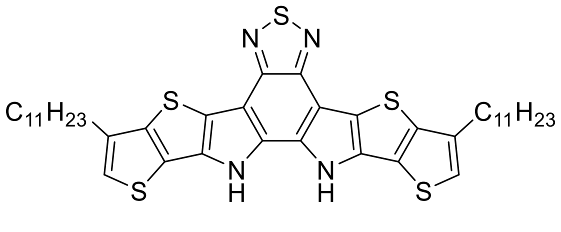

^1H NMR (600 MHz $CDCl_3$ ppm): 11.92 (s, 2H), 6.83 (s, 2H), 2.77 (t, 4H), 1.81 (m, 4H), 1.44-1.22 (m, 32H), 0.87 (d, 6H).

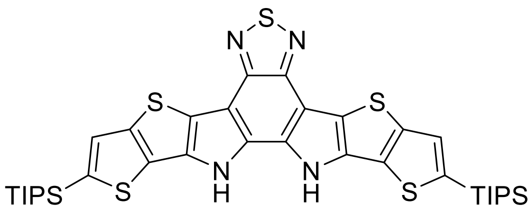

Yield: 94%

^{1}H NMR (400 MHz $CDCl_3$ ppm): 9.42 (s, 2H), 7.50 (s, 2H), 2.77 (t, 4H), 1.43 (m, 6H), 1.18 (d, 36H).

<u>*Adding alkyl chain to the indole*</u>

Because the indole precursor is highly unstable in air, it was used immediately for the next *N*-alkylation step. Specifically, after the indole synthesis, the reaction mixture was added into DMF (final concentration estimated to be ~0.2 M) and then K_2CO_3 (6 eq), KI (0.2 eq) and alkyl bromide (6 eq) were added into this solution. The mixture was then heated at 80 ℃ overnight. Then the solvent was removed and water was added. The product was then extracted by EtOAc and organic phase was dried over Na_2SO_4. The crude product was purified via silica gel column with hexane/EtOAc (10/1) as the eluent.

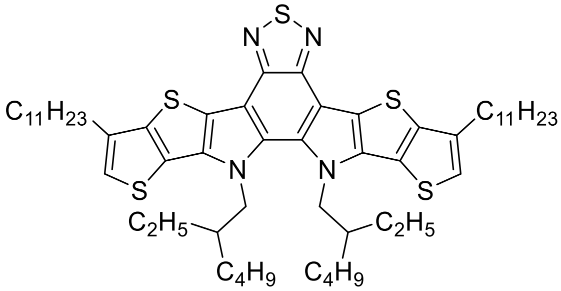

Yield: 88% after two steps

^{1}H NMR (600 MHz, $CDCl_3$) δ 7.01 (s, 2H), 4.60 (t, 4H), 2.82 (t, 4H), 2.07 – 2.01 (m, 2H), 1.86 (m, 4H), 1.26 (m, 36H), 0.95 – 0.78 (m, 18H), 0.61 (m, 12H). Agreed with previously reported (2).

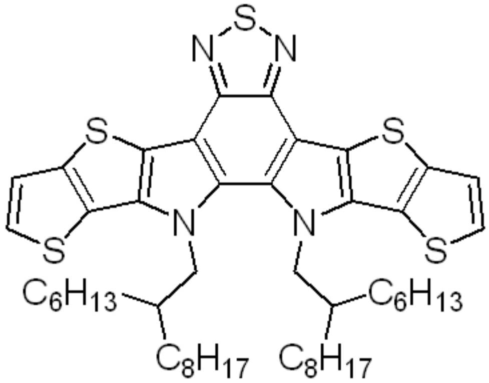

Yield: 96%

^{1}H NMR (600 MHz, $CDCl_3$) δ 7.45 (d, *J* = 5.1 Hz, 2H), 7.43 (d, *J* = 5.2 Hz, 2H), 4.63 (d, 4H), 2.11 – 2.02 (m, 2H), 1.3-0.87 (m, 76H). Agreed with previously reported (32).

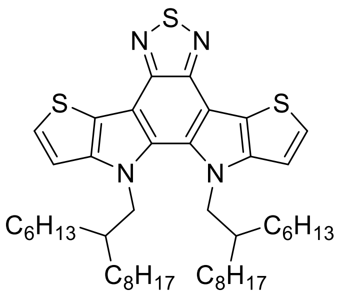

Yield: 90%

^{1}H NMR (400 MHz $CDCl_3$ ppm): 7.42 (d, *J* = 5.2 Hz, 2H), 7.15 (d, *J* = 5.2 Hz, 2H), 4.48 (d, 4H), 2.06-1.93 (m, 2H), 1.25-0.71 (m, 60H). Agreed with previously reported (33).

Synthesis of di-aldehydes

The fused aromatic core (1 mmol) was dissolved in 10 mL chloroform and 1 mL DMF. Oxyphosphorous chloride (20 mmol) was then added dropwise at 0 °C. The reaction was kept stirring at 50 °C overnight. Water was used to quench the reaction slowly and then the mixture was extract with chloroform. The organic phase was filtered through a short path of silica gel. Product was obtained after solvent removal.

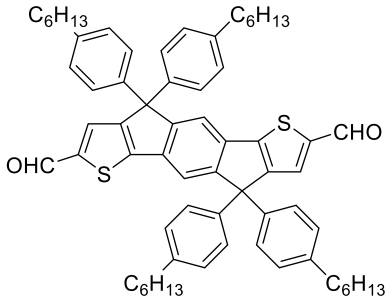

Yield: 96%

^{1}H NMR (400 MHz $CDCl_3$ ppm): 9.82 (s, 2H), 7.66 (s, 2H), 7.56 (s, 2H), 7.90 (q, *J* = 8.4 Hz, 16 H), 2.56 (t, 8H), 1.28 (m, 32H), 0.88 (t, 12H). Agreed with previously reported (7).

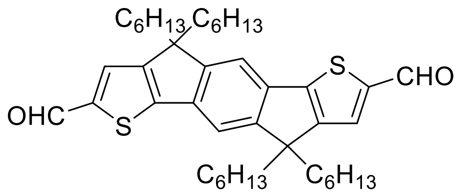

Yield: 90%

^{1}H NMR (600 MHz, $CDCl_3$) δ 9.91 (s 2H), 7.66 – 7.62 (s, 2H), 7.45 (s, 2H), 2.08 – 2.00 (m, 4H), 1.91 (m, 4H), 1.17 – 1.05 (m, 32H), 0.77 (t, 12H). Agreed with previously reported (31).

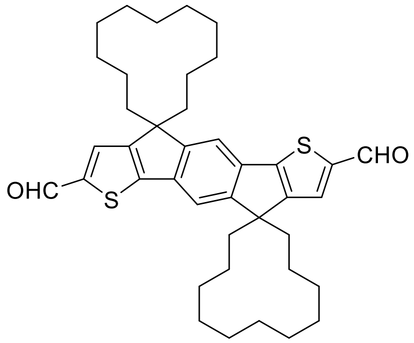

Yield: 88%

^{1}H NMR (400 MHz, $CDCl_3$) δ 9.90 (s, 2H), 7.74 (s, 2H), 7.59 (s, 2H), 1.75 (m, 8H), 0.91 – 0.75 (m, 36H).

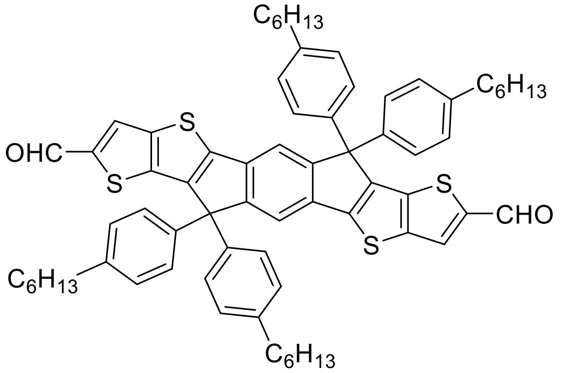

Yield: 90%

^{1}H NMR (400 MHz, $CDCl_3$) δ 9.90 (s, 2H), 7.94 (s, 2H), 7.63 (s, 2H), 7.15 (q, J = 8.4 Hz, 16H), 2.58 (t, 8H), 1.59 (m, 8H), 1.28 (m, 24H), 0.89 (t, 12H).

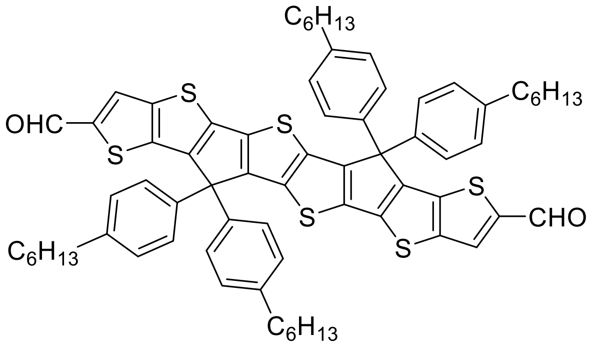

Yield: 92%

^{1}H NMR (400 MHz, $CDCl_3$) δ 9.90 (s, 2H), 7.94 (s, 2H), 7.15 (s, 16H), 2.58 (t, J = 7.9 Hz, 8H), 1.59 (m, 8H), 1.28 (m, 24H), 0.89 (t, 12H).

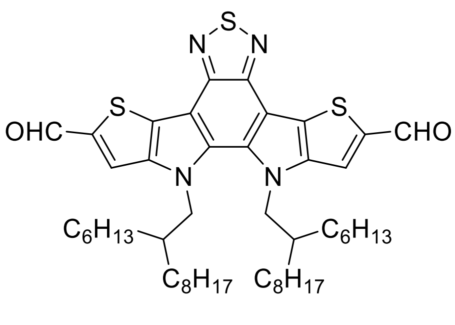

Yield: 90%

^{1}H NMR (400 MHz, $CDCl_3$) δ 10.02 (s, 2H), 7.82 (s, 2H), 4.53 (d, 4H), 2.07 – 1.93 (m, 2H), 1.24 – 0.69 (m, 60H). Agreed with previously reported (33).

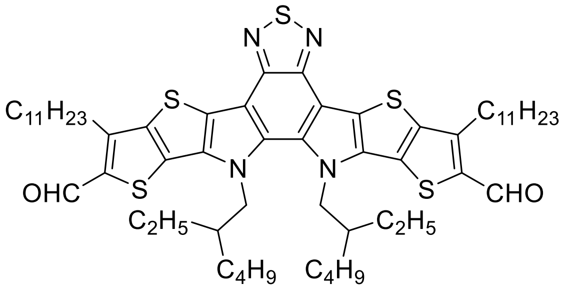

Yield: 92%

^{1}H NMR (600 MHz, $CDCl_3$) δ 10.14 (s, 2H), 4.68 – 4.58 (m, 4H), 3.20 (t, 4H), 2.00 (m, 2H), 1.93 (m, 4H), 1.51 – 1.42 (m, 4H), 1.38 (m, 4H), 1.34 – 1.19 (m, 24H), 0.93 – 0.77 (m, 16H), 0.63 (dtd, 12H). Agreed with previously reported (2).

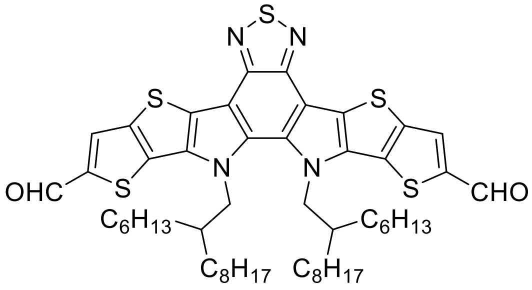

Yield: 86%

^{1}H NMR (600 MHz, $CDCl_3$) δ 10.03 (s, 2H), 7.82(s, 2H), 4.53 (d, 4H), 1.99 (m, 2H), 1.24-0.73 (m, 60H). Agreed with previously reported (32).

Synthesis of A-D-A molecules

The dialdehyde (0.1 mmol), end-group units (0.3 mmol) and proline (30 %mmol) were mixed in 5 mL chloroform and 0.5 mL ethanol. The reaction was typically completed within 1h with sonication. Upon solvent removal, a large amount of ethanol was used to wash out the unreacted end-group units and proline to obtain the condensed products as the precipitate. Centrifuge is used to isolate the solid. Silica gel column chromatography with hexane/toluene as eluent could be used to further purify the products.

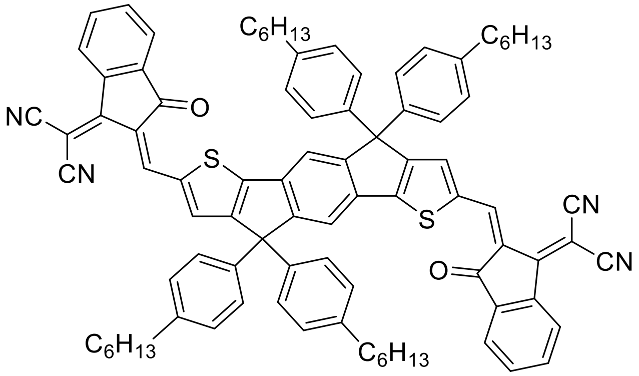

Yield: 95%

^{1}H NMR (400 MHz $CDCl_3$ ppm): 8.90 (s, 2H), 8.69 (d, *J* = 6.5 Hz, 2H), 7.92 (d, *J* = 6.5 Hz, 2H),7.77-7.71 (m, 8H), 7.13 (d+d, *J* = 8.3 Hz, 16H), 2.58 (t, 8H), 1.28 (m, 32 H), 0.88 (t, 12H). Agreed with previously reported (7).

HRMS([m]·) m/z calcd. for C90H82N4O2S2 1314.58792, found 1314.58724.

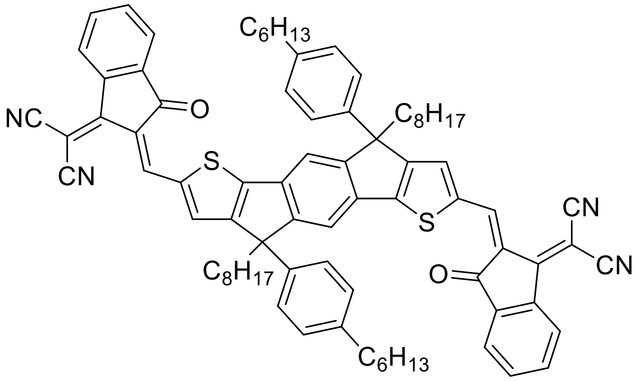

Yield: 97%

^{1}H NMR (600 MHz, $CDCl_3$ppm) δ 8.93 (s, 2H), 8.70 (d, *J* = 6.5 Hz, 2H), 7.94 (d, *J* = 6.5 Hz, 2H), 7.81 – 7.73 (m, 4H), 7.67 (s, 2H), 7.56 (s+s, 2H), 7.22 – 7.09 (m, 8H), 2.62 – 2.48 (m, 6H), 2.26 (dtd, 2H), 1.64 – 1.56 (m, 4H), 1.40 – 1.10 (m, 36H), 0.90 – 0.78 (m, 12H).

HRMS([m]·) m/z calcd. for C82H82N4O2S2 1218.58792, found 1218.58875.

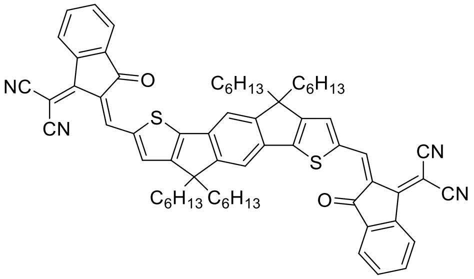

Yield: 96%

^{1}H NMR (400 MHz, $CDCl_3$) δ 8.99 (s, 2H), 8.72 (d, *J* = 6.3 Hz, 2H), 7.95 (dd, *J* = 6.3 Hz, 2H), 7.82 – 7.74 (m, 4H), 7.73 (s, 2H), 7.60 (s, 2H), 2.07 (d, *J* = 4.5 Hz, 4H), 1.95 (s, 4H), 1.12 (s, 32H), 0.78 (t, *J* = 6.7 Hz, 12H). Agreed with previously reported (31).

HRMS([m]·) m/z calcd. for C66H66N4O2S2 1010.46272, found 1010.46199.

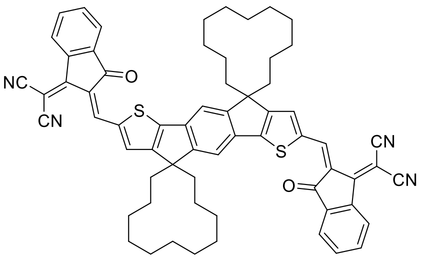

Yield: 95%
^{1}H NMR (400 MHz, $CDCl_3$) δ 9.01 (d, 2H), 8.74 (d, *J* = 6.3 Hz, 2H), 7.97 (d, *J* = 6.3 Hz, 2H), 7.85 – 7.72 (m, 8H), 1.81 (m, 8H), 1.28-0.81 (m, 36H).

HRMS([m]·) m/z calcd. for C64H58N4O2S2 978.40012, found 978.40090.

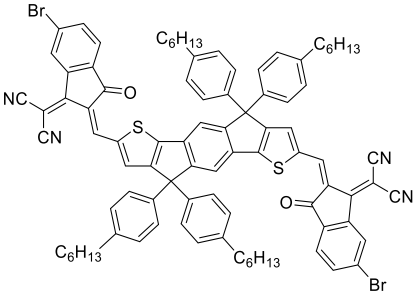

Yield: 95%
^{1}H NMR (600 MHz, $CDCl_3$) δ 8.82 (d, *J* = 1.4 Hz, 2H), 7.87 (dd, *J* = 8.0, 1.5 Hz, 2H), 7.76 (d, *J* = 8.0 Hz, 2H), 7.73 (s+s, 4H), 7.17 – 7.07 (m, 16H), 2.58 (t, 8H), 1.63 – 1.57 (m, 8H), 1.38 – 1.23 (m, 24H), 0.91 – 0.81 (m, 12H).
HRMS([m]·) m/z calcd. for C90H80Br2N4O2S2 1470.40895, found 1470.40815.

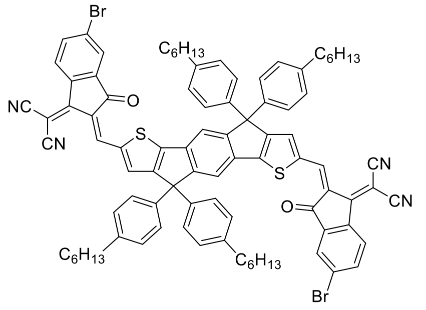

Yield: 94%

^{1}H NMR (600 MHz, $CDCl_3$) δ 8.90 (s, 2H), 8.53 (d, , *J* = 8.4 Hz, 2H), 7.99 (d, , *J* = 1.9 Hz, 2H), 7.84 (dd, *J* = 8.4, 1.9 Hz, 2H), 7.73 (s+s, 4H), 7.13 (d+d, 16H), 2.58 (t, 8H), 1.60 (dd, 8H), 1.29 (m, 24H), 0.92 – 0.81 (t, 12H).

HRMS([m]·) m/z calcd. for C90H80Br2N4O2S2 1470.40895, found 1470.40812.

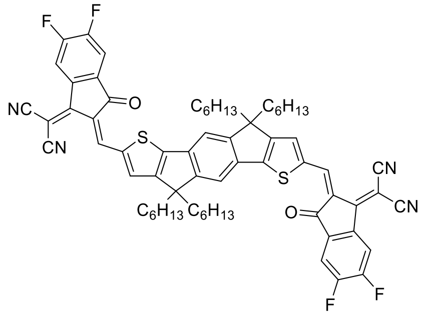

Yield: 95%
^{1}H NMR (600 MHz, $CDCl_3$) δ 8.98 (s, 2H), 8.57 (dd, *J* = 9.8, 6.3 Hz, 2H), 7.78 – 7.68 (m, 4H), 7.62 (s, 2H), 2.13 – 2.03 (m, 4H), 2.00 – 1.91 (m, 4H), 1.15 (m, 26H), 0.78 (t, *J* = 7.0 Hz, 12H).
HRMS([m]·) m/z calcd. for C66H62F2N4O2S2 1082.42503, found 1082.402485.

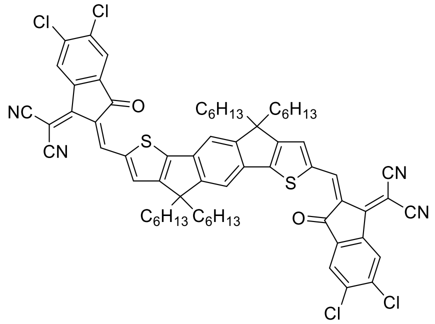

Yield: 95%
^{1}H NMR (600 MHz, $CDCl_3$) δ 9.03 (s, 2H), 8.83 (s, 2H), 8.00 (s, 2H), 7.77 (s, 2H), 7.65 (s, 2H), 2.14 – 2.07 (m, 4H), 2.02 – 1.95 (m, 4H), 1.23 – 1.07 (m, 26H), 0.80 (t, 12H).
HRMS([m]·) m/z calcd. for C66H62Cl4N4O2S2 1146.30683, found 1146.30621.

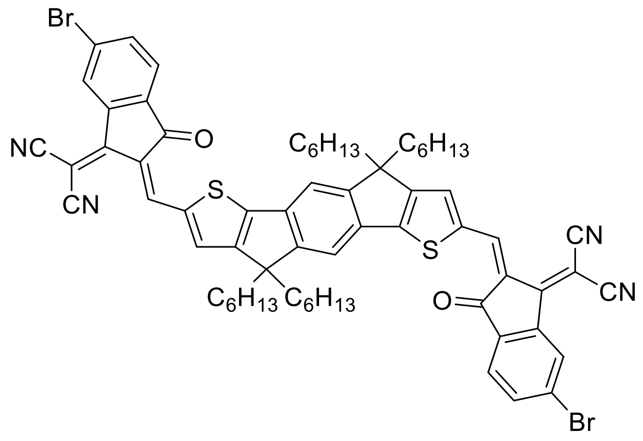

Yield: 93%

^{1}H NMR (600 MHz, $CDCl_3$) δ 9.02 (s, 2H), 8.87 (d, *J* = 1.5 Hz, 2H), 7.91 (dd, *J* = 7.9, 1.5 Hz, 2H), 7.82 (d, *J* = 8.0 Hz, 2H), 7.76 (s, 2H), 7.63 (d, *J* = 1.5 Hz, 2H), 2.13 – 2.07 (m, 4H), 2.00 – 1.94 (m, 4H), 1.16 (m, 32H), 0.80 (t, 12H).

HRMS([m]·) m/z calcd. for C66H64Br2N4O2S2 1166.68375, found 1166.68329.

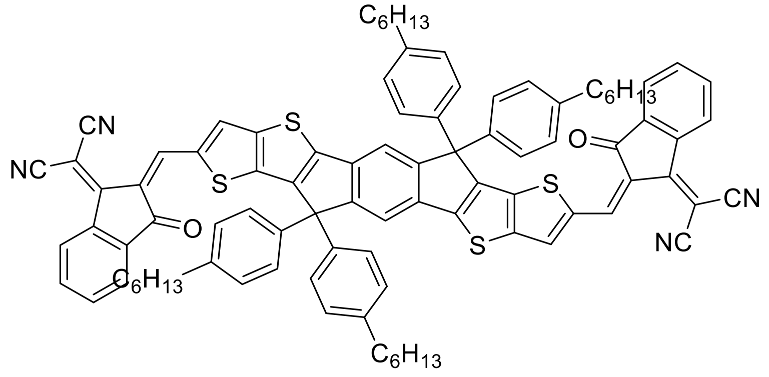

Yield: 93%
^{1}H NMR (600 MHz, $CDCl_3$) δ 8.86 (s, 2H), 8.68 (d, *J* = 7.6 Hz, 2H), 8.22 (s, 2H), 7.94 – 7.89 (m, 2H), 7.75 (dt, *J* = 19.2, 7.3 Hz, 4H), 7.63 (s, 2H), 7.23 – 7.06 (m, 16H), 2.56 (t, 8H), 1.59 (t, 8H), 1.38 – 1.21 (m, 24H), 0.93 – 0.78 (m, 12H). Agreed with that previously reported (1).

HRMS([m]·) m/z calcd. for C94H82N4O2S4 1426.53206, found 1426.53129.

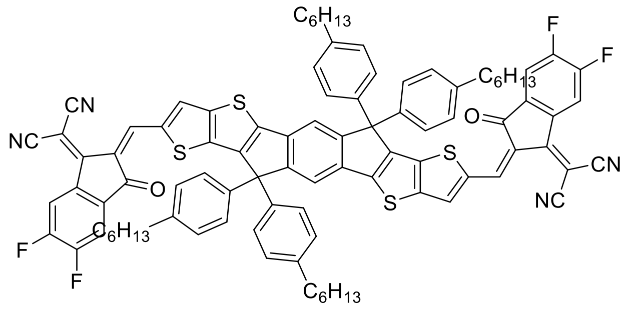

Yield: 90%

^{1}H NMR (600 MHz, $CDCl_3$) δ 8.85 (s, 2H), 8.53 (dd, 2H), 8.23 (s, 2H), 7.68 (t, 2H), 7.65 (s, 2H), 7.22 – 7.10 (m, 16H), 2.60 – 2.53 (m, 8H), 1.62 – 1.57 (m, 8H), 1.39 – 1.14 (m, 24H), 0.88 – 0.80 (m, 12H). Agreed with that previously reported (34).

HRMS([m]·) m/z calcd. for C94H78F4N4O2S24 1498.49438, found 1498.49342.

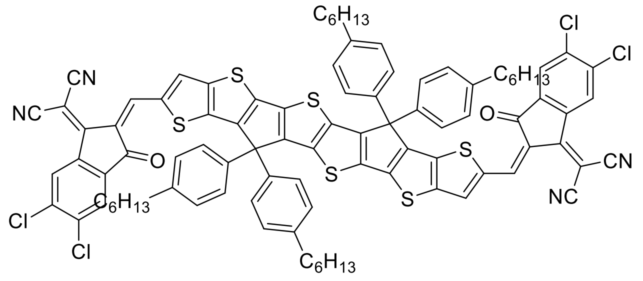

Yield: 99%

^{1}H NMR (400 MHz, $CDCl_3$) δ 8.85 (s, 2H), 8.75 (s, 2H), 8.19 (s, 2H), 7.93 (s, 2H), 7.18 (m, 16H), 2.61 – 2.53 (m, 8H), 1.58 (m 8H), 1.27 (m 24H), 0.85 (t, 12H). Agreed with previously reported (35).

HRMS([m]·) m/z calcd. for C94H76Cl4N4O2S8 1624.30467, found 1624.30401.

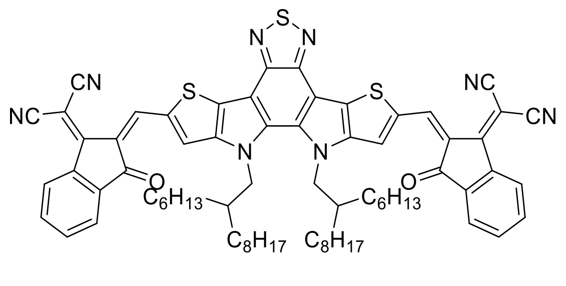

Yield: 94%

^{1}H NMR (400 MHz, $CDCl_3$) δ 8.97 (s, 2H), 8.74 – 8.66 (m, 2H), 8.04 (s, 2H), 8.01 – 7.94 (m, 2H), 7.82 – 7.74 (m, 4H), 4.56 (d, *J* = 7.8 Hz, 4H), 2.08 (d, *J* = 10.2 Hz, 2H), 1.22 – 0.83 (m, 51H), 0.79 (s, 6H), 0.72 (s, 6H).

HRMS([m]·) m/z calcd. for C72H78N8O2S3 1182.54099, found 1182.54003.

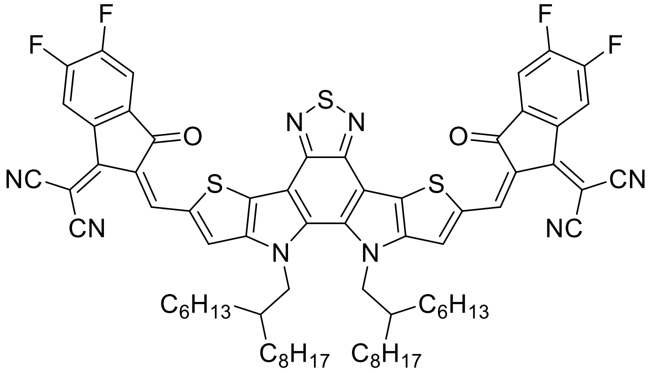

Yield: 98%

^{1}H NMR (600 MHz, $CDCl_3$) δ 8.92 (s, 2H), 8.53 (dd, 2H), 7.99 (s, 2H), 7.74 – 7.67 (m, 2H), 4.57 (d, 4H), 2.11 (s, 2H), 1.22 – 0.82 (m, 48H), 0.79 (t, 6H), 0.73 (t, 6H). Agreed with previously reported (33).

HRMS([m]·) m/z calcd. for C72H74F4N8O2S3 1254.50330, found 1254.50237.

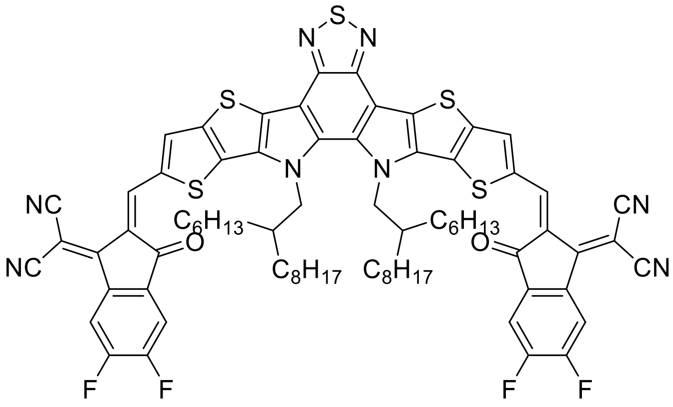

Yield: 94%

^{1}H NMR (600 MHz, $CDCl_3$) δ 9.02 (s, 2H), 8.58 (dd, 2H), 8.25 (s, 2H), 7.72 (t, 2H), 4.76 (d, 4H), 2.10 (d, 2H), 1.18 – 0.75 (m, 54H), 0.68 (t, *J* = 7.1 Hz, 6H).Agreed with previously reported (32).

HRMS([m]·) m/z calcd. for C76H74F4N8O2S5 1366.44744, found 1366.447682.

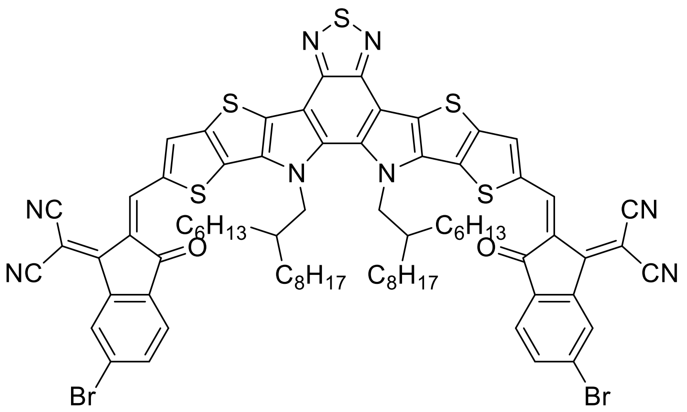

Yield: 92%

^{1}H NMR (600 MHz, $CDCl_3$) δ 9.04 (s, 2H), 8.85 (s, 2H), 8.24 (s, 2H), 7.90 (d, *J* = 7.9 Hz, 2H), 7.81 (d, *J* = 7.9 Hz, 2H), 4.76 (d, 4H), 2.11 (m, 2H), 1.25 – 0.85 (m, 48H), 0.78 (t, 3H), 0.68 (t, 3H).

HRMS([m]·) m/z calcd. for C76H76Br2N8O2S5 1450.30616, found 1450.30539.

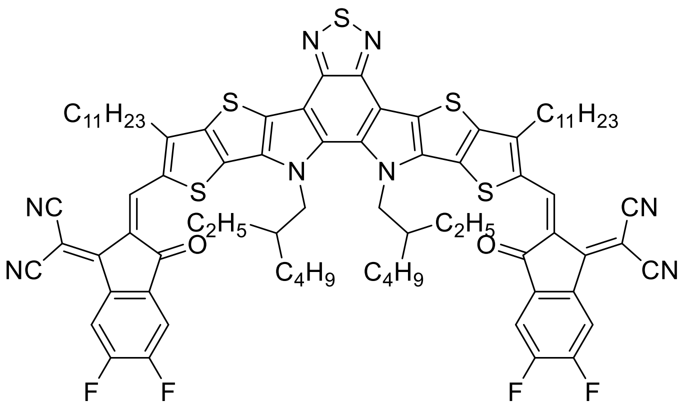

Yield: 92%

^{1}H NMR (600 MHz, $CDCl_3$) δ 9.14 (s, 2H), 8.55 (dd, 2H), 7.71 (t, 2H), 4.78 (t, 4H), 3.22 (t, 4H), 2.16 – 2.06 (m, 2H), 1.93 – 1.83 (m, 4H), 1.50 (t, 4H), 1.27-0.92 (m, 44H), 0.91 – 0.81 (m, 6H), 0.76 (q, 6H), 0.67 (td, 6H). Agreed with that previously reported (2).

HRMS([m]·) m/z calcd. for C82H86F4N8O2S5 1450.54134, found 1450.354066.

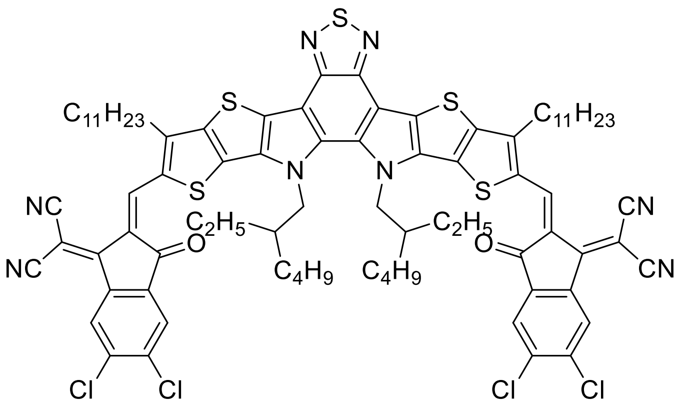

Yield: 88%

^{1}H NMR (600 MHz, $CDCl_3$) δ 9.22 (s, 2H), 8.84 (s, 2H), 8.00 (s, 2H), 4.79 (m, 4H), 3.28 (t, 4H), 2.11 (m, 2H), 1.91 (m, 4H), 1.52 (m, 4H), 1.38-1.00 (m 44H), 0.86 (t, 6H), 0.77 (t, 6H), 0.68 (t, 6H). Agreed with that previously reported (36).

HRMS([m]·) m/z calcd. for C82H86Cl4N8O2S5 1514.42314, found 1514.42262.

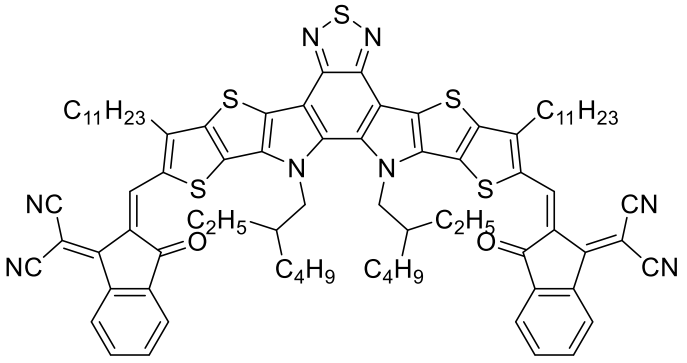

Yield: 89%

^{1}H NMR (600 MHz, $CDCl_3$) δ 9.20 (s, 2H), 8.75 – 8.71 (m, 2H), 7.97 – 7.94 (m, 2H), 7.80 – 7.74 (m, 4H), 4.77 (t, 4H), 3.25 (t, 4H), 2.09 (m, 2H), 1.89 (m, 4H), 1.52 (m, 4H), 1.43 –1.01 (m, 44H), 0.86 (t, J = 7.0 Hz, 6H), 0.73 (t, 6H), 0.66 (t, 6H). Agreed with that previously reported (37).

HRMS([m]·) m/z calcd. for C82H90N8O2S5 1378.57903, found 1378.57808.

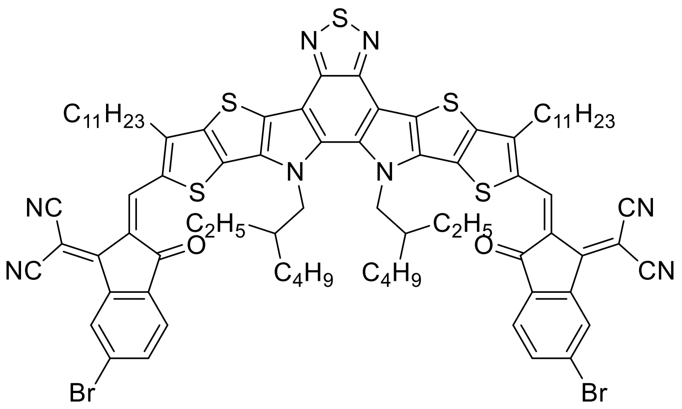

Yield: 91%

^{1}H NMR (600 MHz, $CDCl_3$) δ 9.16 (s, 2H), 8.83 (s, 2H), 7.88 (d, 2H), 7.80 (d, 2H), 4.77 (m, 4H), 3.22 (t, 4H), 2.16 (m, 2H), 1.87 (m, 4H), 1.49 (m, 4H), 1.37 (m, 4H), 1.32 – 0.96 (m, 40H) 0.86 (t, 6H), 0.75 (m, 6H), 0.72 – 0.59 (m, 6H). Agreed with previously reported (38).

HRMS([m]·) m/z calcd. for C82H88Br2N8O2S5 1534.40006, found 1534.39028.

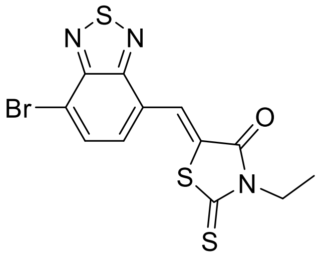

Yield: 84%

^{1}H NMR (400 MHz $CDCl_3$ ppm): 8.43 (s, 1H), 7.98 (d, *J* = 7.6 Hz, 1H), 7.53 (d, *J* = 7.8 Hz, 1H), 4.24 (q, 2H), 1.32 (t, 3H).

HRMS([m]·) m/z calcd. for C12H8BrN2OS2 384.90129, found 384.90208.

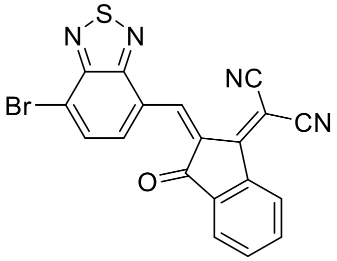

Yield: 80%

^{1}H NMR (400 MHz $CDCl_3$ ppm): 9.40 (s, 1H), 8.86 (d, *J* = 7.8 Hz, 1H), 8.76 (d, J = 7.9 Hz, 1H), 8.06 (d, J = 7.7 Hz, 1H), 7.97 (d, J = 7.4 Hz, 1H), 7.91 – 7.87 (m, 1H), 7.83 (dd, J = 8.2, 6.7 Hz, 1H).

HRMS([m]·) m/z calcd. for C19H7BrN4OS 417.95240, found 417.95117.

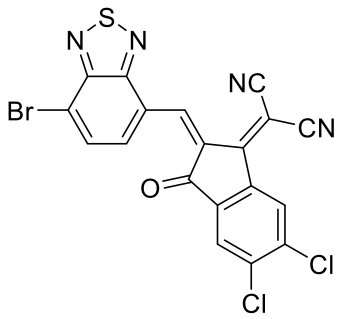

Yield: 82%

^{1}H NMR (400 MHz $CDCl_3$ ppm): 9.46 (s, 1H), 8.85 (d+s, *J* = 7.8 Hz, 2H), 8.07 (d, *J* = 7.8 Hz, 1H), 8.02 (s, 1H).

HRMS([m]·) m/z calcd. for C19H5BrCl2N4OS 485.87445, found 485.87347.

NMR spectra

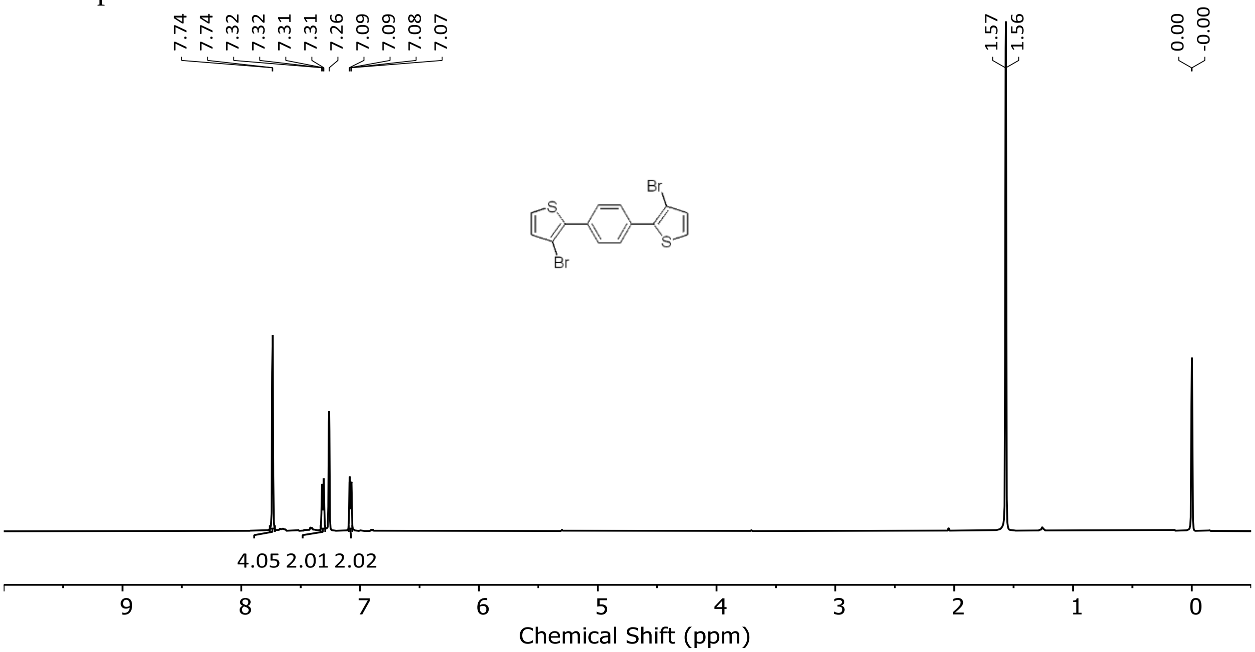

7.74
7.74
7.32
7.32
7.31
7.31
7.26
7.09
7.09
7.08
7.07
1.57
1.56
0.00
-0.00
Br
S
S
Br
4.05 2.01 2.02
9 8 7 6 5 4 3 2 1 0
Chemical Shift (ppm)

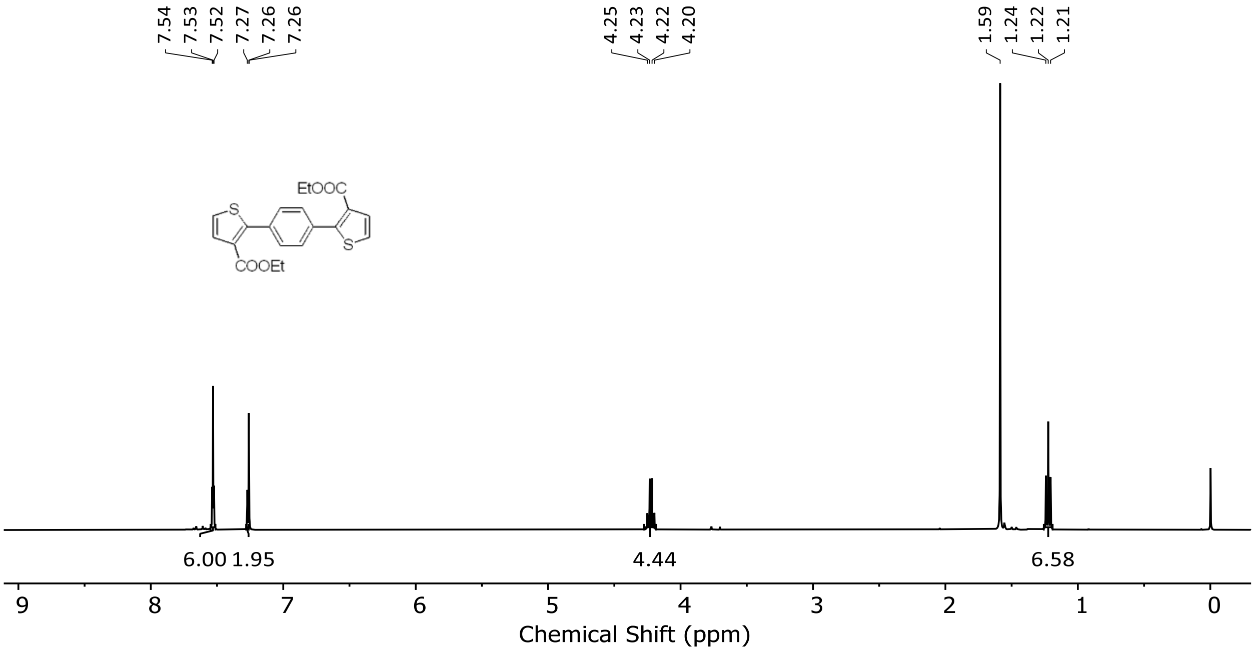

7.54
7.53
7.52
7.27
7.26
7.26
4.25
4.23
4.22
4.20
1.59
1.24
1.22
1.21
EtOOC
S
S
COOEt
6.00 1.95
4.44
6.58
9 8 7 6 5 4 3 2 1 0
Chemical Shift (ppm)

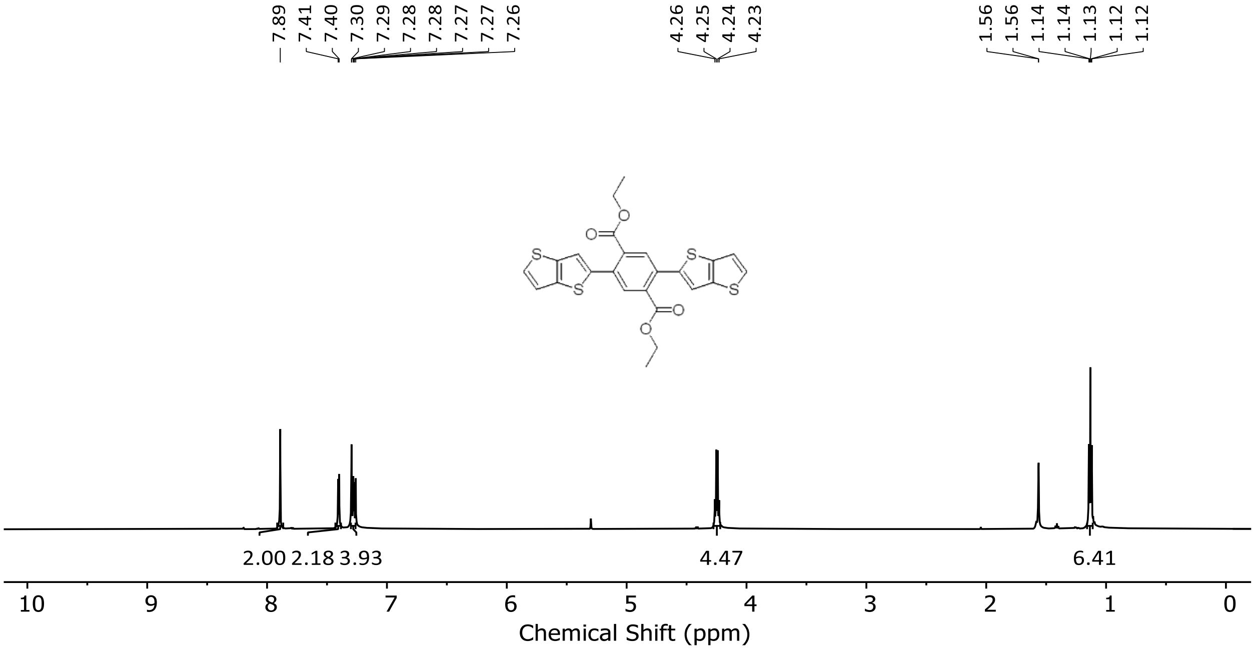

7.89
7.41
7.40
7.30
7.29
7.28
7.28
7.27
7.27
7.26
4.26
4.25
4.24
4.23
1.56
1.56
1.14
1.14
1.13
1.12
1.12
S
S
S
S
O
O
O
O
2.00 2.18 3.93
4.47
6.41
10 9 8 7 6 5 4 3 2 1 0
Chemical Shift (ppm)

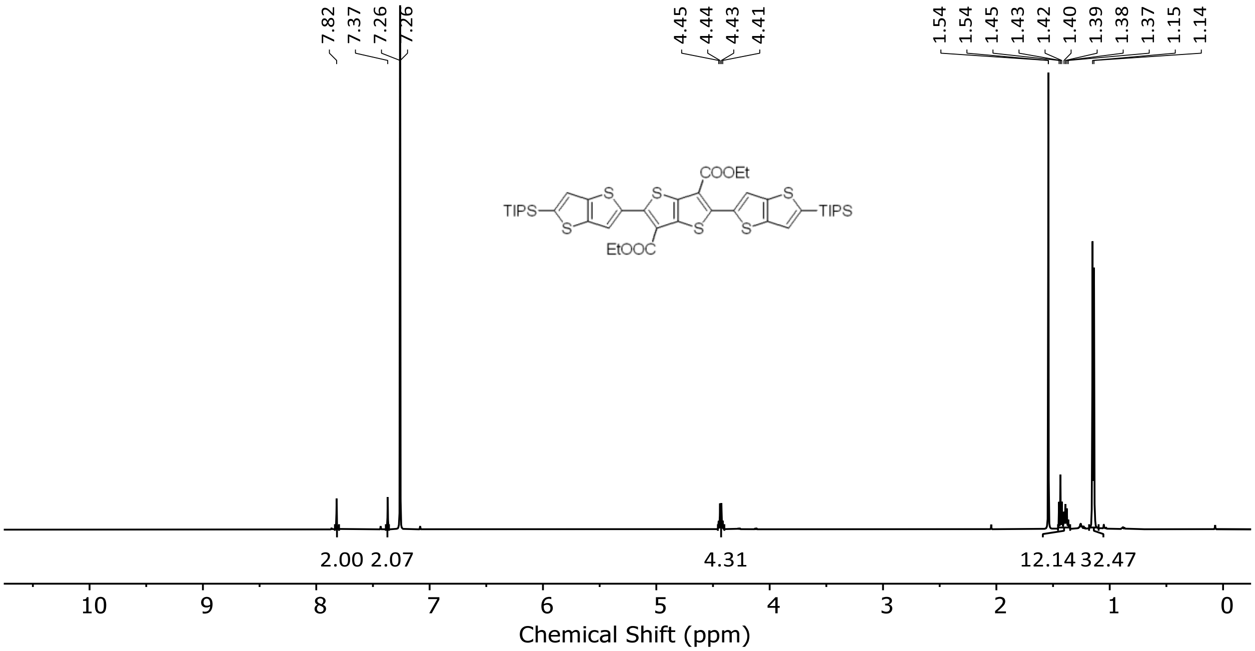

7.82
7.37
7.26
7.26
4.45
4.44
4.43
4.41
1.54
1.54
1.45
1.43
1.42
1.40
1.39
1.38
1.37
1.15
1.14
COOEt
TIPS
TIPS
EtOOC
S
S
S
S
S
S
2.00 2.07
4.31
12.14 32.47
10 9 8 7 6 5 4 3 2 1 0
Chemical Shift (ppm)

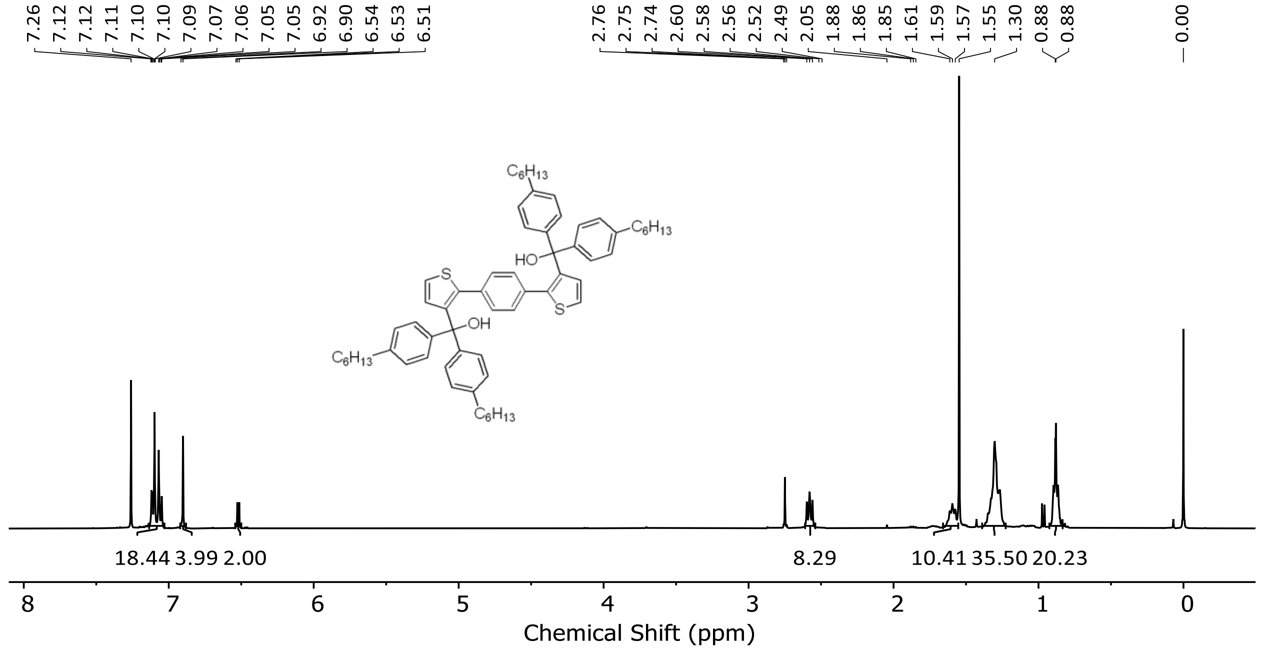
7.26
7.12
7.12
7.11
7.10
7.10
7.09
7.07
7.06
7.05
7.05
6.92
6.90
6.54
6.53
6.51
2.76
2.75
2.74
2.60
2.58
2.56
2.52
2.49
2.05
1.88
1.86
1.85
1.61
1.59
1.57
1.55
1.30
0.88
0.88
0.00
C6H13
HO
OH
S
18.44 3.99 2.00
8.29
10.41 35.50 20.23
8 7 6 5 4 3 2 1 0
Chemical Shift (ppm)

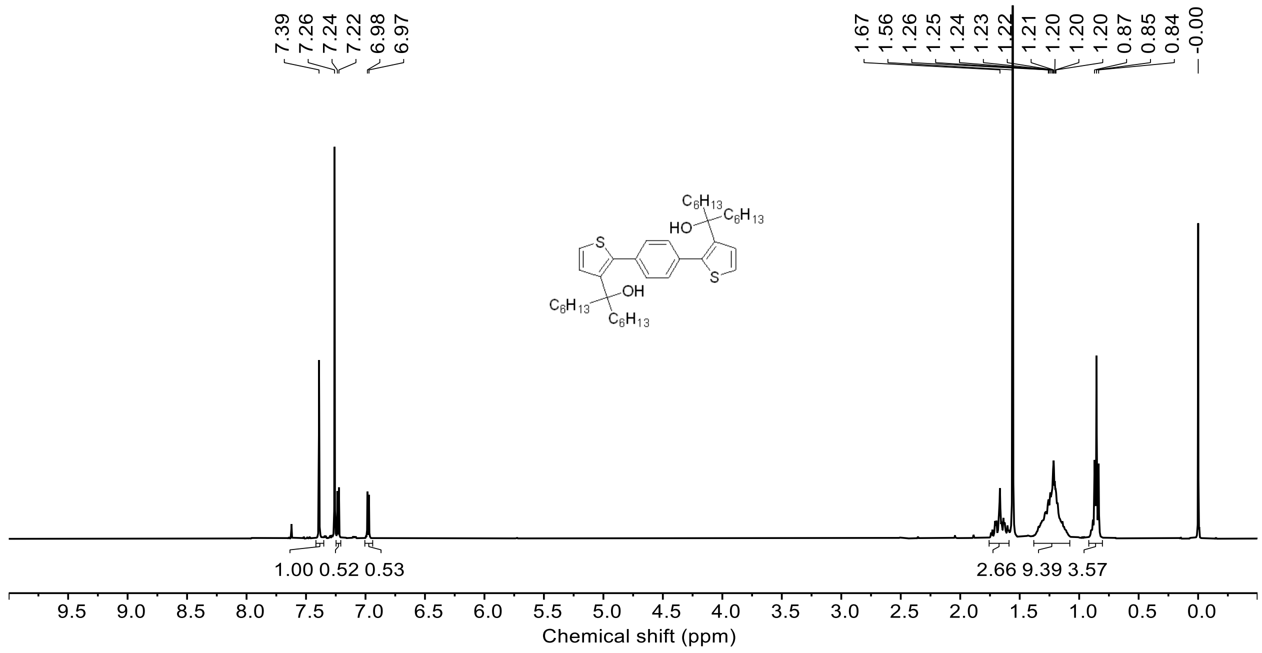
7.39
7.26
7.24
7.22
6.98
6.97
1.67
1.56
1.26
1.25
1.24
1.23
1.22
1.21
1.20
1.20
1.20
0.87
0.85
0.84
-0.00
C6H13
HO
OH
S
1.00 0.52 0.53
2.66 9.39 3.57
9.5 9.0 8.5 8.0 7.5 7.0 6.5 6.0 5.5 5.0 4.5 4.0 3.5 3.0 2.5 2.0 1.5 1.0 0.5 0.0
Chemical shift (ppm)

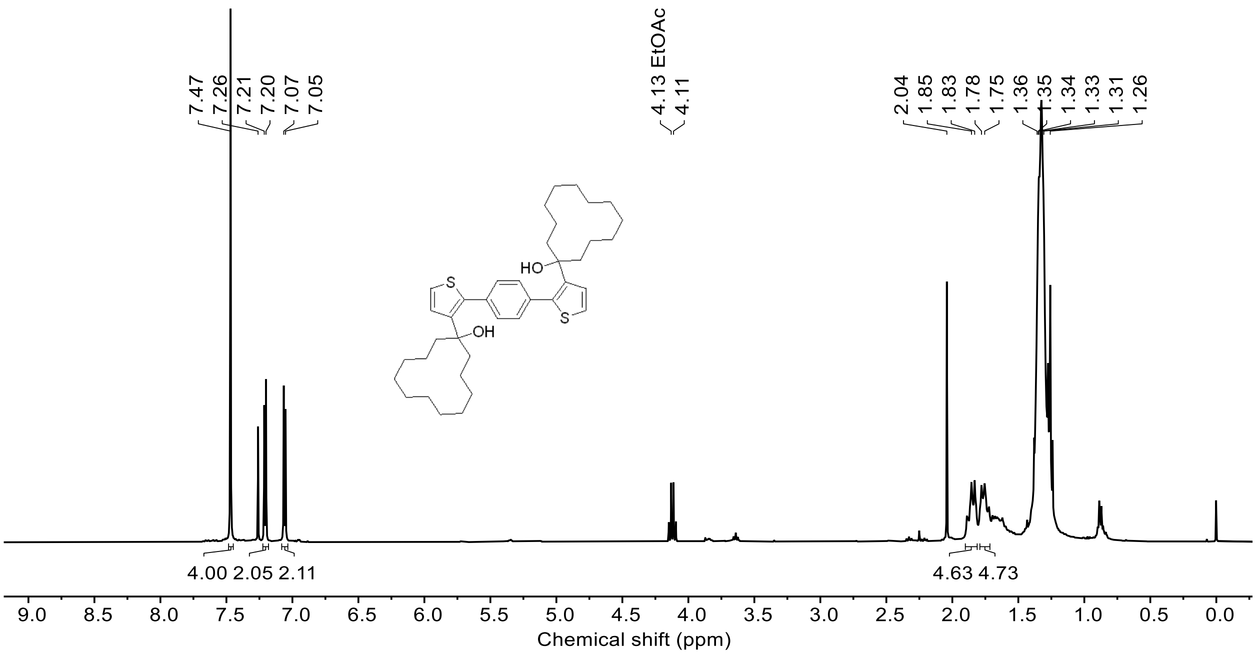

7.47
7.26
7.21
7.20
7.07
7.05
4.13 EtOAc
4.11
2.04
1.85
1.83
1.78
1.75
1.36
1.35
1.34
1.33
1.31
1.26
HO
OH
S
4.00 2.05 2.11
4.63 4.73
9.0 8.5 8.0 7.5 7.0 6.5 6.0 5.5 5.0 4.5 4.0 3.5 3.0 2.5 2.0 1.5 1.0 0.5 0.0
Chemical shift (ppm)

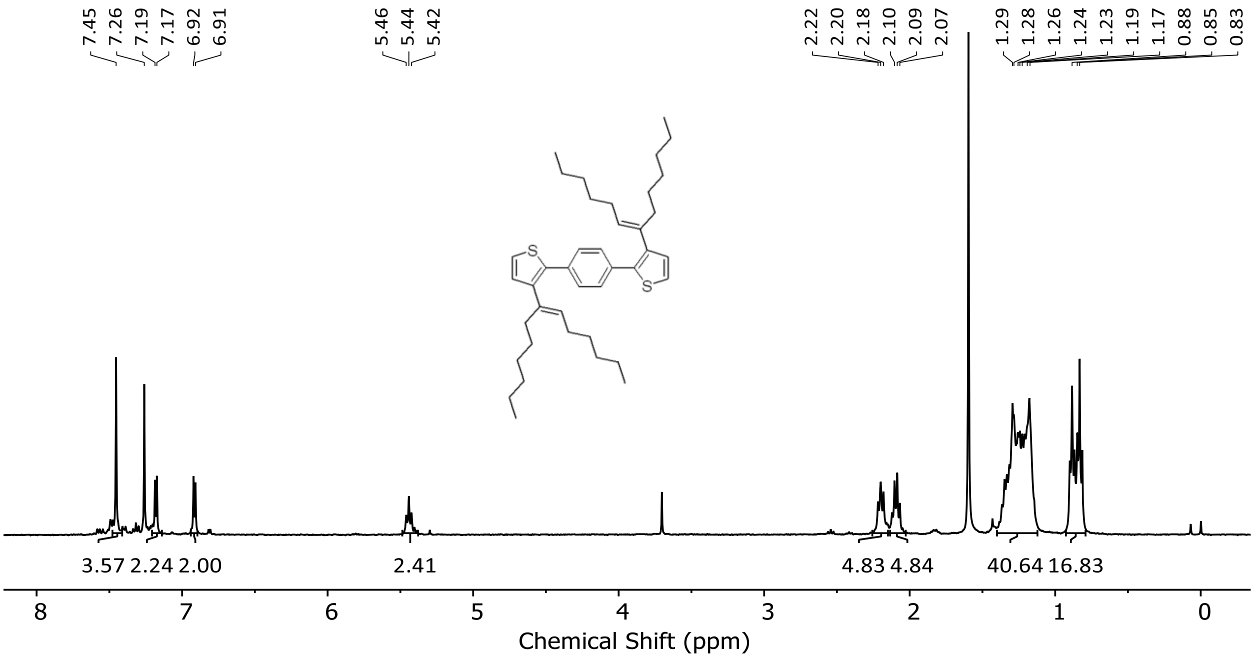

7.45
7.26
7.19
7.17
6.92
6.91
5.46
5.44
5.42
2.22
2.20
2.18
2.10
2.09
2.07
1.29
1.28
1.26
1.24
1.23
1.19
1.17
0.88
0.85
0.83
S
3.57 2.24 2.00
2.41
4.83 4.84
40.64 16.83
8 7 6 5 4 3 2 1 0
Chemical Shift (ppm)

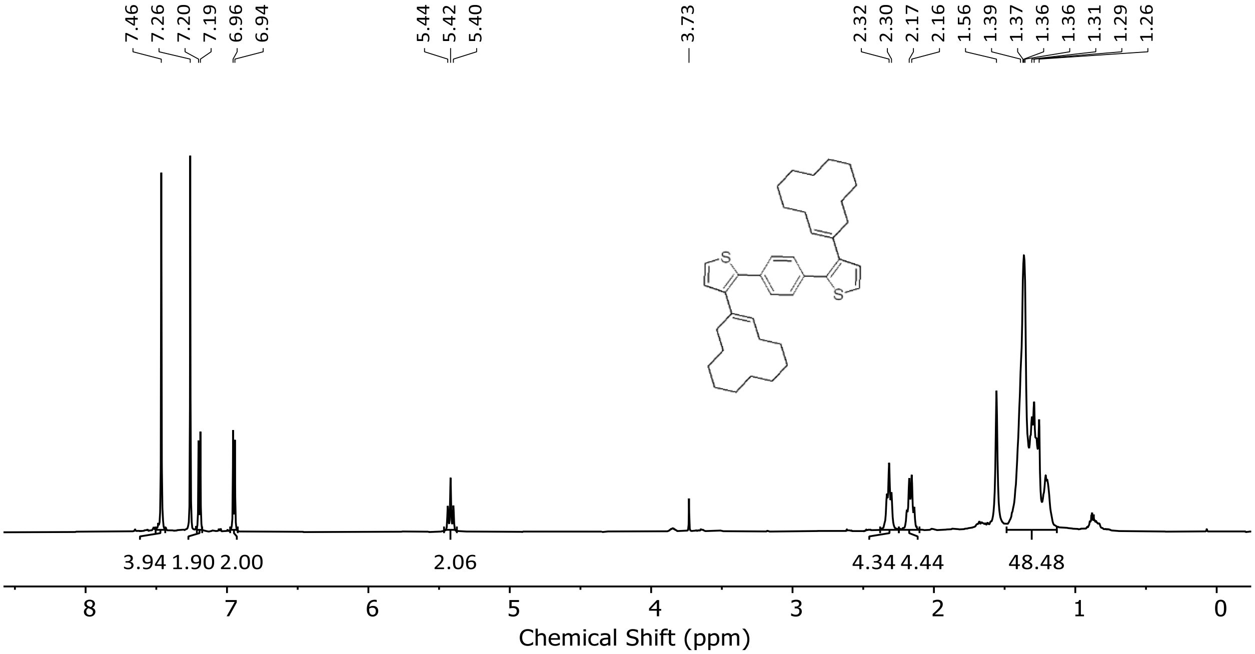

7.46 7.26 7.20 7.19 6.96 6.94
5.44 5.42 5.40
3.73
2.32 2.30 2.17 2.16 1.56 1.39 1.37 1.36 1.36 1.31 1.29 1.26
S
S
3.94 1.90 2.00
2.06
4.34 4.44
48.48
8 7 6 5 4 3 2 1 0
Chemical Shift (ppm)

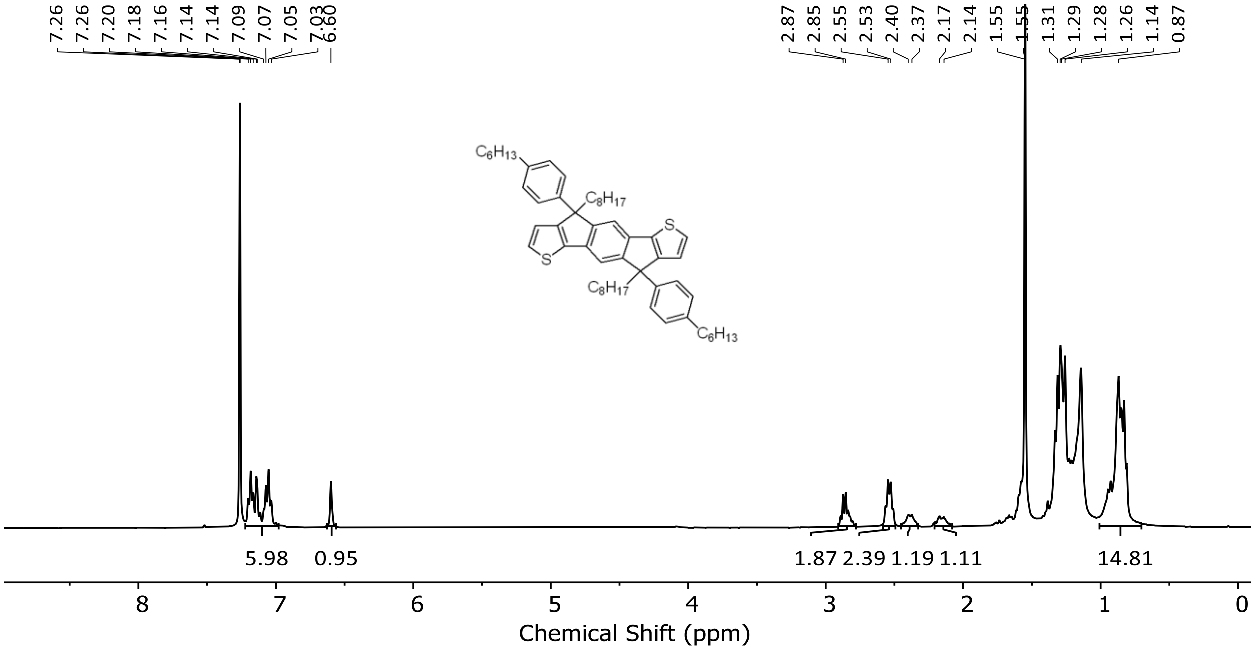

7.26 7.26 7.20 7.18 7.16 7.14 7.14 7.09 7.07 7.05 7.03 6.60
2.87 2.85 2.55 2.53 2.40 2.37 2.17 2.14 1.55 1.55 1.31 1.29 1.28 1.26 1.14 0.87
C_6H_{13}
C_8H_{17}
S
S
C_8H_{17}
C_6H_{13}
5.98 0.95
1.87 2.39 1.19 1.11
14.81
8 7 6 5 4 3 2 1 0
Chemical Shift (ppm)

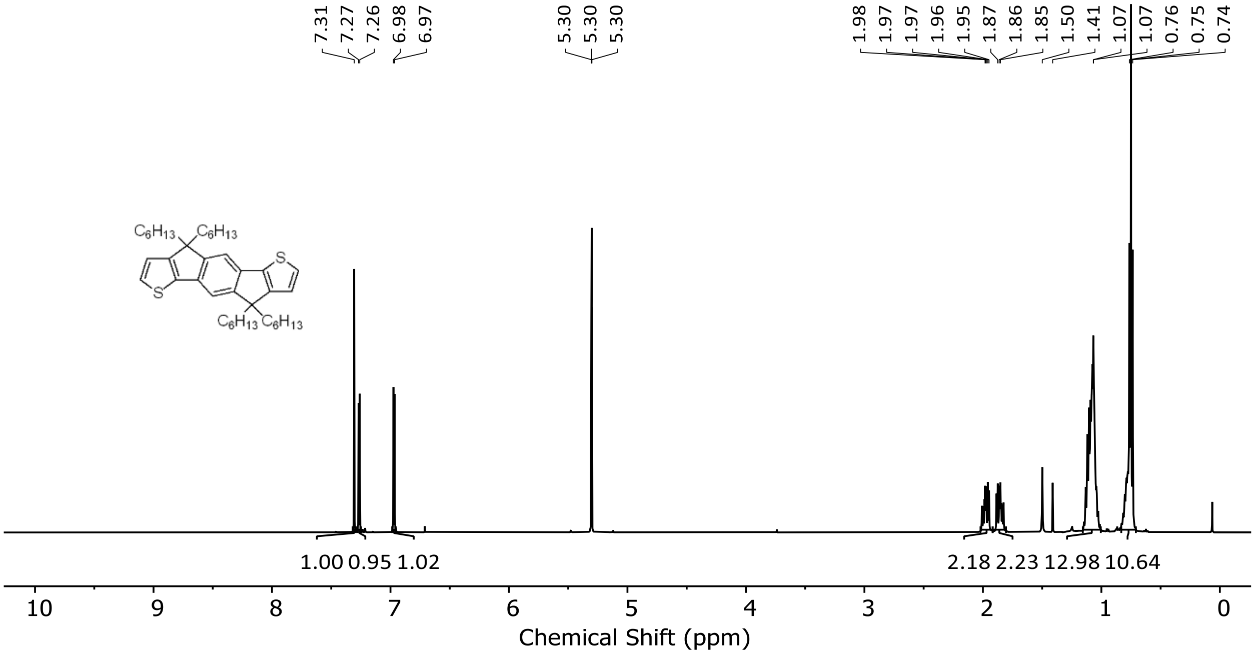
7.31 7.27 7.26 6.98 6.97
5.30 5.30 5.30
1.98 1.97 1.97 1.96 1.95 1.87 1.86 1.85 1.50 1.41 1.07 1.07 0.76 0.75 0.74
C_6H_{13} C_6H_{13} S S C_6H_{13} C_6H_{13}
1.00 0.95 1.02
2.18 2.23 12.98 10.64
10 9 8 7 6 5 4 3 2 1 0
Chemical Shift (ppm)

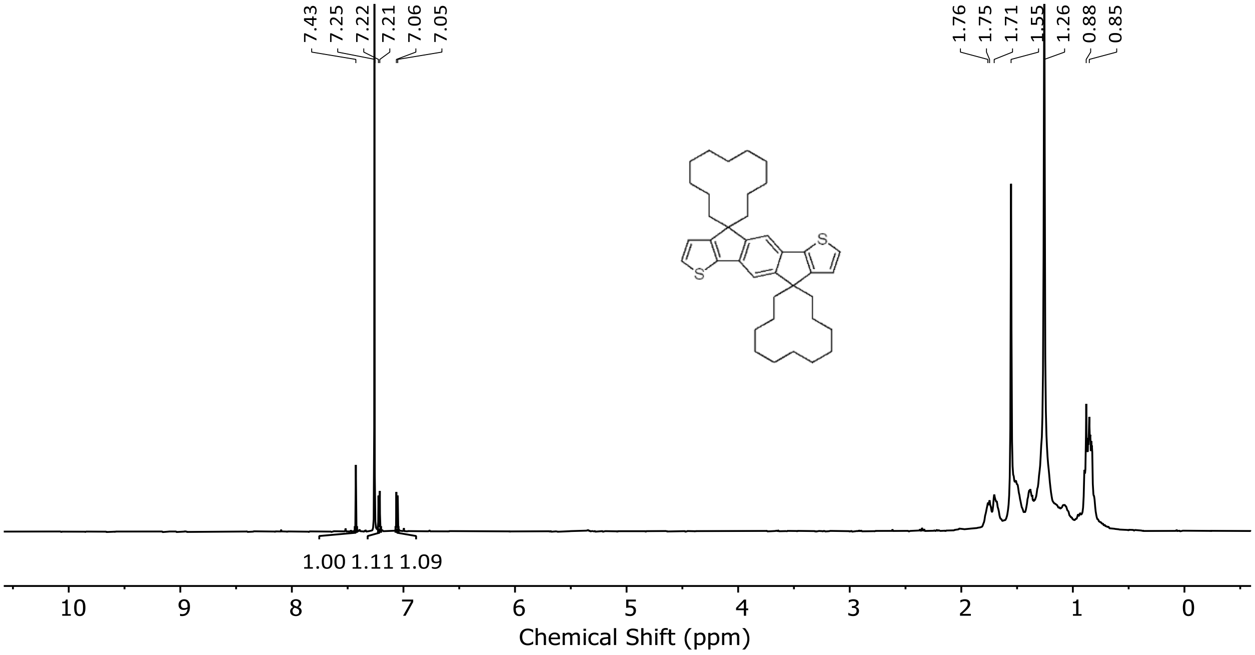
7.43 7.25 7.22 7.21 7.06 7.05
1.76 1.75 1.71 1.55 1.26 0.88 0.85
S S
1.00 1.11 1.09
10 9 8 7 6 5 4 3 2 1 0
Chemical Shift (ppm)

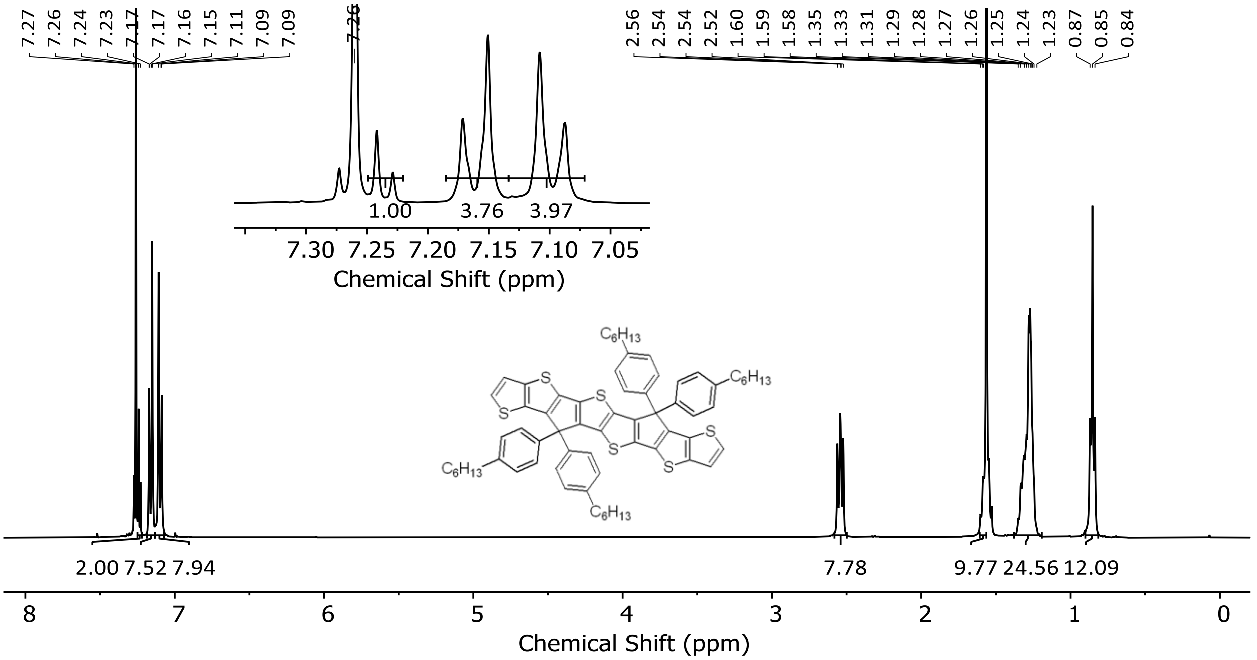
7.27
7.26
7.24
7.23
7.17
7.17
7.16
7.15
7.11
7.09
7.09
7.26
1.00
3.76
3.97
7.30 7.25 7.20 7.15 7.10 7.05
Chemical Shift (ppm)
2.56
2.54
2.54
2.52
1.60
1.59
1.58
1.35
1.33
1.31
1.29
1.28
1.27
1.26
1.25
1.24
1.23
0.87
0.85
0.84
C6H13
2.00 7.52 7.94
7.78
9.77 24.56 12.09
8 7 6 5 4 3 2 1 0
Chemical Shift (ppm)

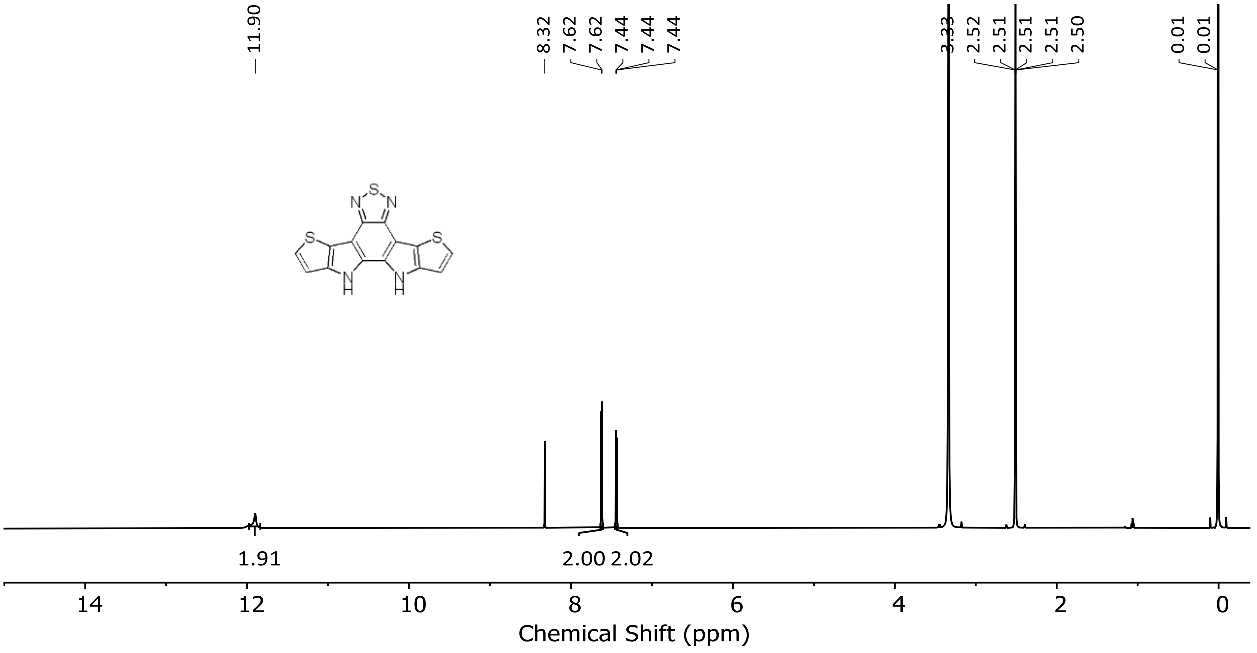
11.90
8.32
7.62
7.62
7.44
7.44
7.44
3.33
2.52
2.51
2.51
2.51
2.50
0.01
0.01
1.91
2.00 2.02
14 12 10 8 6 4 2 0
Chemical Shift (ppm)

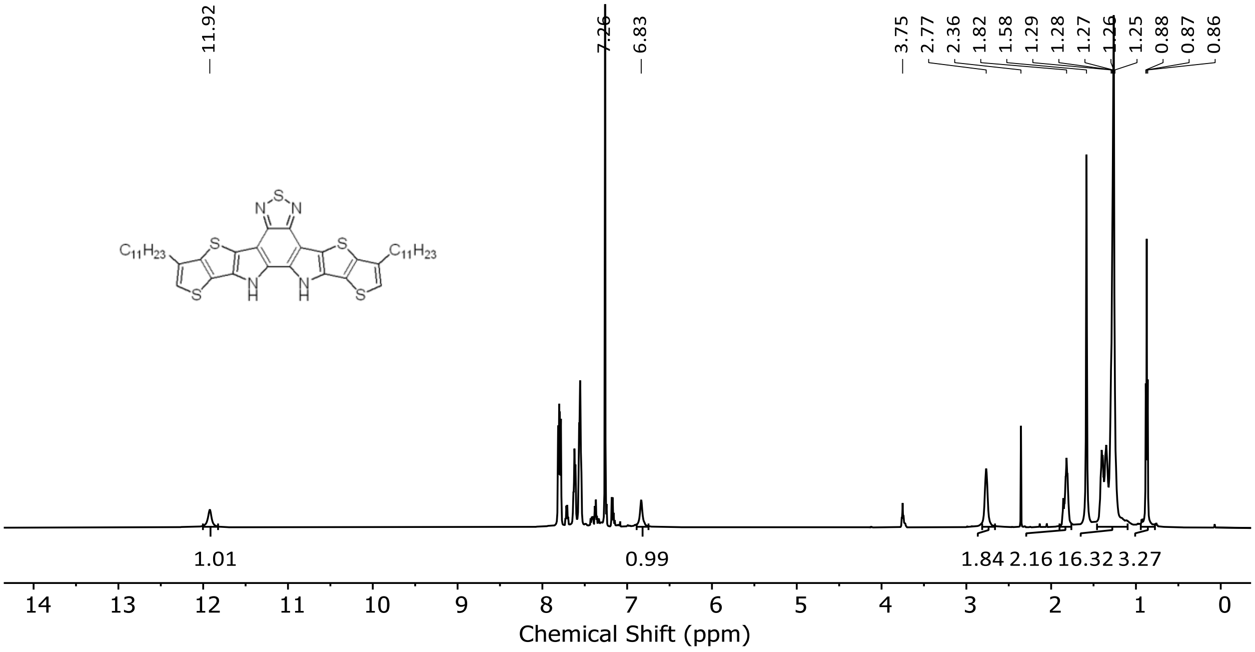

11.92
7.26
6.83
3.75
2.77
2.36
1.82
1.58
1.29
1.28
1.27
1.26
1.25
0.88
0.87
0.86
C11H23
S
N
H
1.01
0.99
1.84
2.16
16.32
3.27
14
13
12
11
10
9
8
7
6
5
4
3
2
1
0
Chemical Shift (ppm)

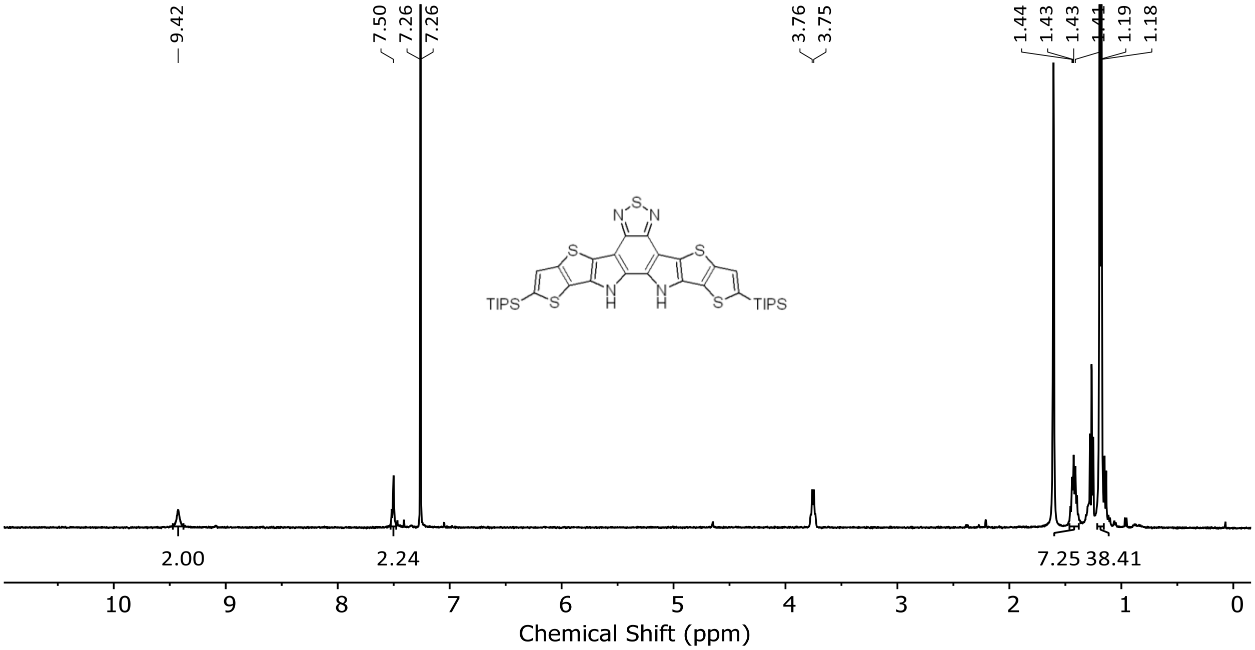

9.42
7.50
7.26
7.26
3.76
3.75
1.44
1.43
1.43
1.41
1.19
1.18
TIPS
S
N
H
2.00
2.24
7.25
38.41
10
9
8
7
6
5
4
3
2
1
0
Chemical Shift (ppm)

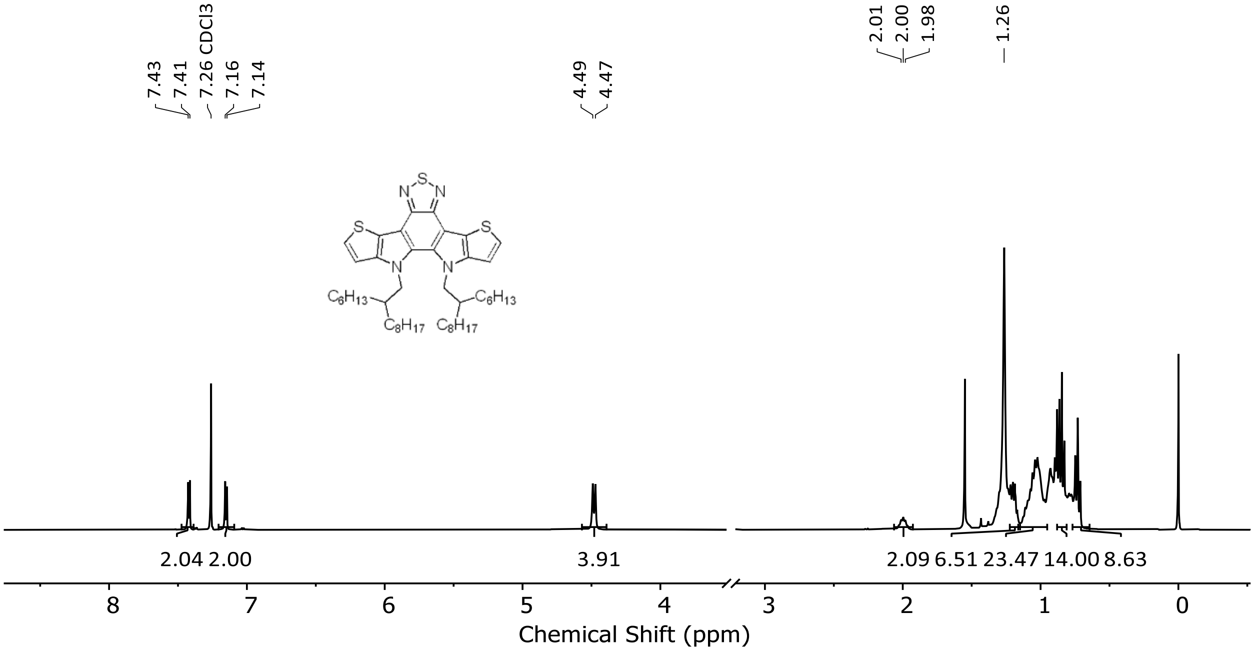
7.43
7.41
7.26 CDCl3
7.16
7.14
4.49
4.47
2.01
2.00
1.98
1.26
2.04 2.00
3.91
2.09 6.51 23.47 14.00 8.63
Chemical Shift (ppm)

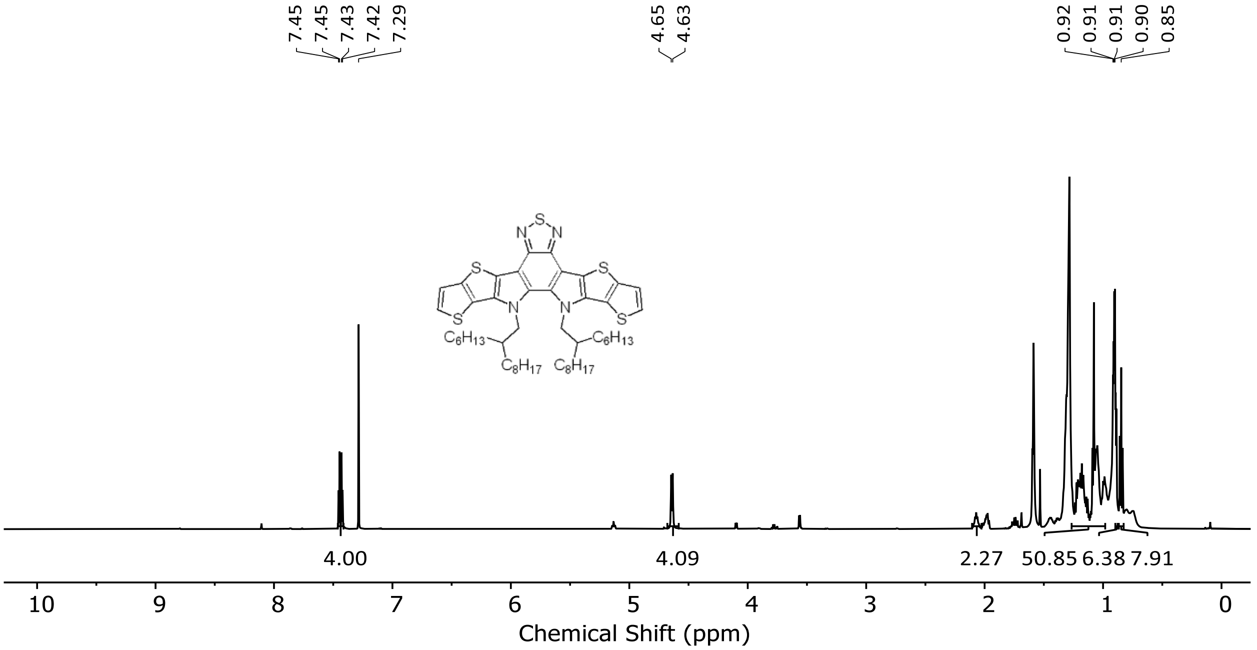
7.45
7.45
7.43
7.42
7.29
4.65
4.63
0.92
0.91
0.91
0.90
0.85
4.00
4.09
2.27 50.85 6.38 7.91
Chemical Shift (ppm)

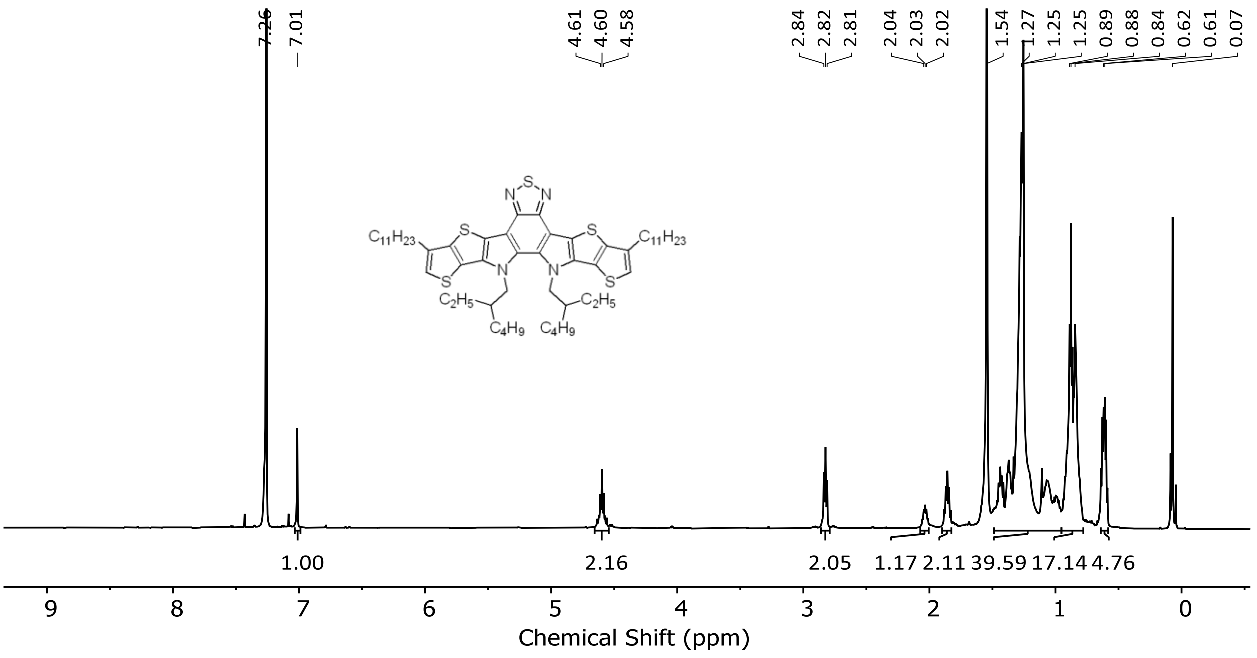

7.26
7.01
4.61
4.60
4.58
2.84
2.82
2.81
2.04
2.03
2.02
1.54
1.27
1.25
1.25
0.89
0.88
0.84
0.62
0.61
0.07
1.00
2.16
2.05
1.17
2.11
39.59
17.14
4.76
9
8
7
6
5
4
3
2
1
0
Chemical Shift (ppm)

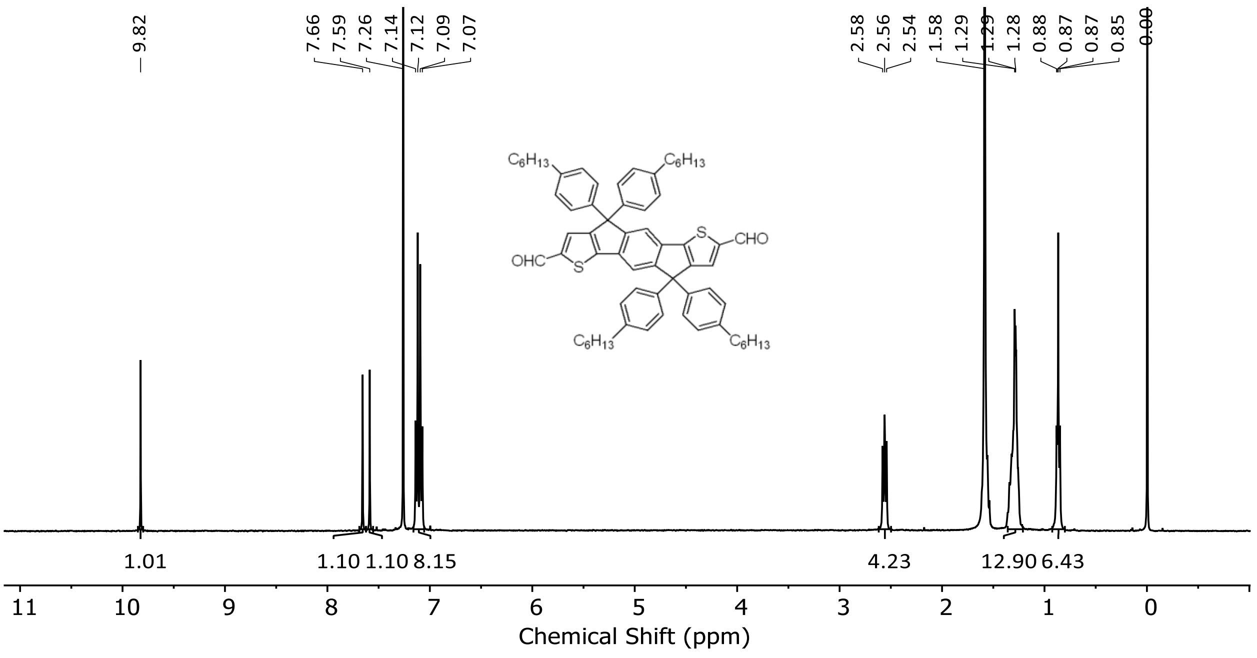

9.82
7.66
7.59
7.26
7.14
7.12
7.09
7.07
2.58
2.56
2.54
1.58
1.29
1.29
1.28
0.88
0.87
0.87
0.85
0.00
1.01
1.10
1.10
8.15
4.23
12.90
6.43
11
10
9
8
7
6
5
4
3
2
1
0
Chemical Shift (ppm)

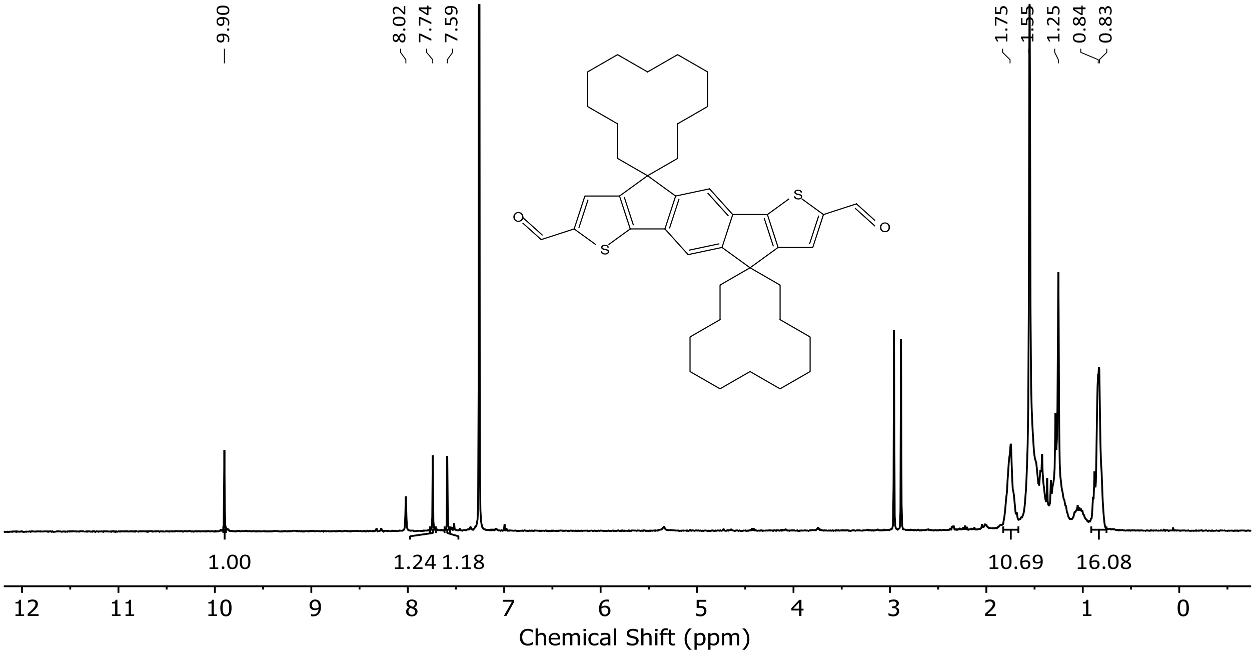
9.90
8.02
7.74
7.59
1.75
1.55
1.25
0.84
0.83
1.00
1.24 1.18
10.69
16.08
12 11 10 9 8 7 6 5 4 3 2 1 0
Chemical Shift (ppm)

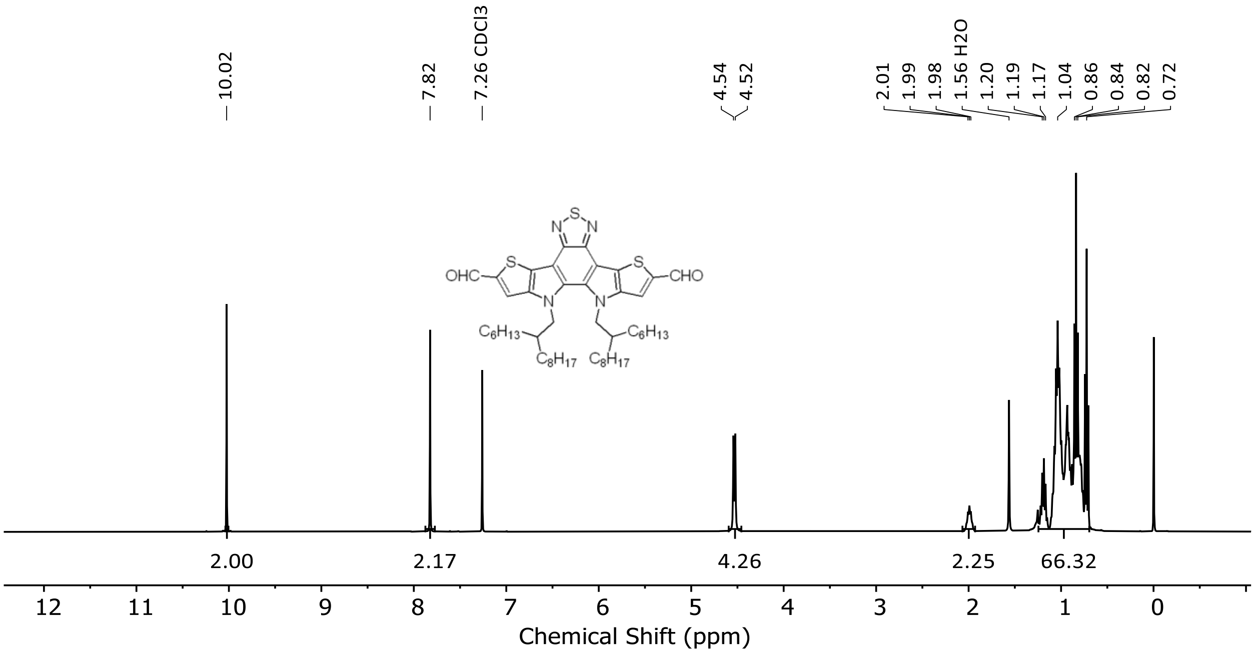
10.02
7.82
7.26 CDCl3
4.54
4.52
2.01
1.99
1.98
1.56 H2O
1.20
1.19
1.17
1.04
0.86
0.84
0.82
0.72
2.00
2.17
4.26
2.25
66.32
12 11 10 9 8 7 6 5 4 3 2 1 0
Chemical Shift (ppm)

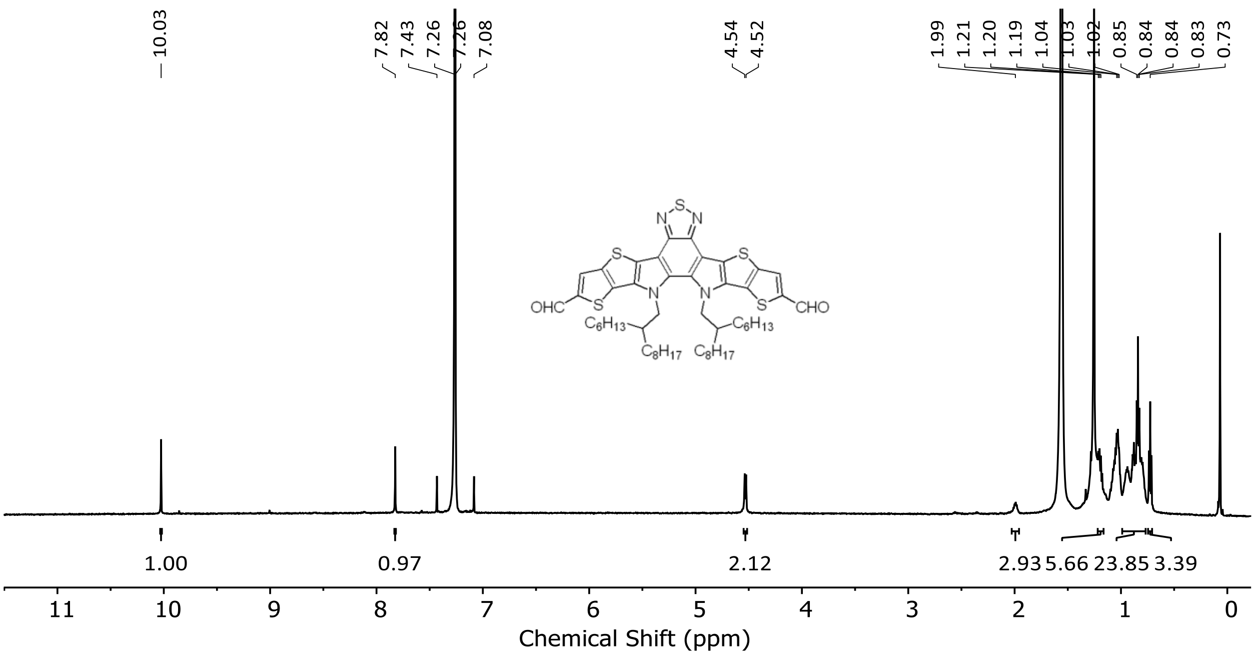
10.03
7.82
7.43
7.26
7.26
7.08
4.54
4.52
1.99
1.21
1.20
1.19
1.04
1.03
1.02
0.85
0.84
0.84
0.83
0.73
OHC
CHO
C_6H_{13}
C_8H_{17}
1.00
0.97
2.12
2.93
5.66
23.85
3.39
11 10 9 8 7 6 5 4 3 2 1 0
Chemical Shift (ppm)

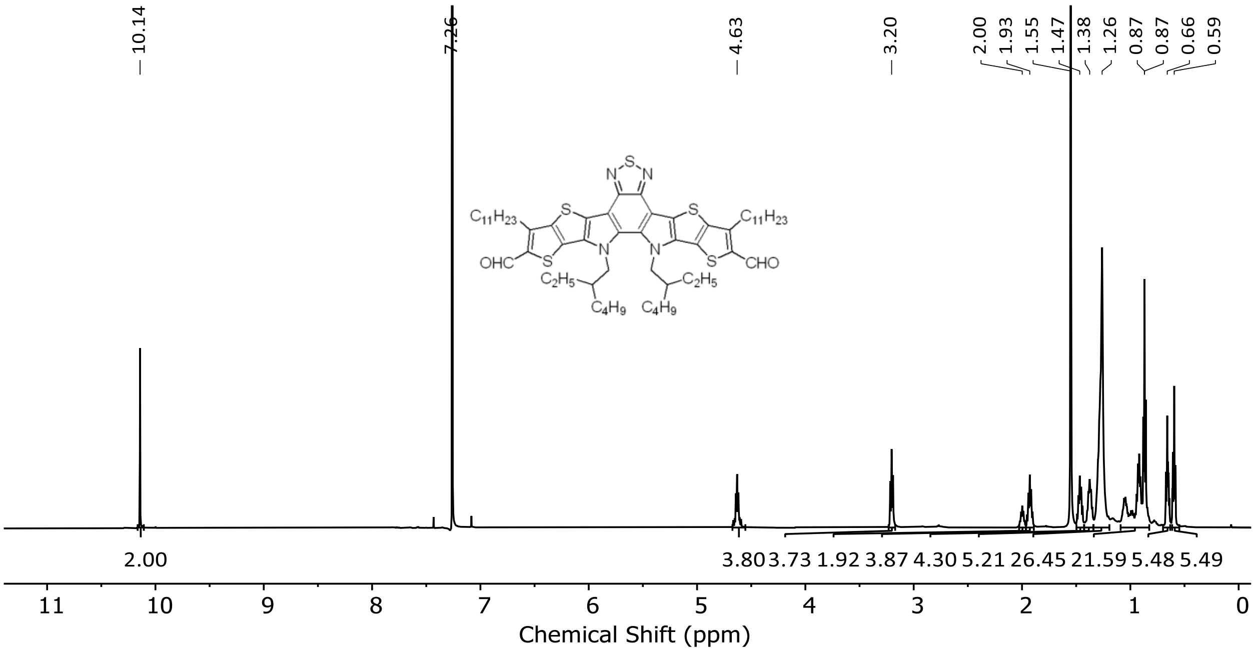
10.14
7.26
4.63
3.20
2.00
1.93
1.55
1.47
1.38
1.26
0.87
0.87
0.66
0.59
$C_{11}H_{23}$
OHC
CHO
C_2H_5
C_4H_9
2.00
3.80
3.73
1.92
3.87
4.30
5.21
26.45
21.59
5.48
5.49
11 10 9 8 7 6 5 4 3 2 1 0
Chemical Shift (ppm)

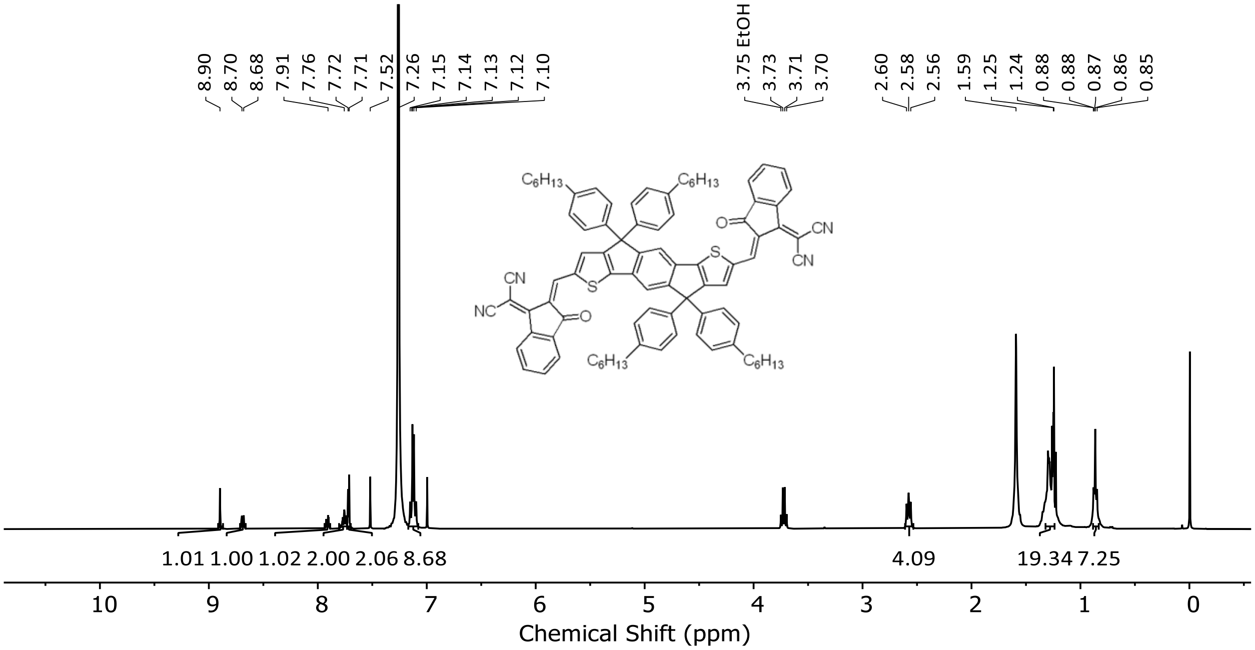
8.90 8.70 8.68 7.91 7.76 7.72 7.71 7.52 7.26 7.15 7.14 7.13 7.12 7.10
3.75 EtOH 3.73 3.71 3.70
2.60 2.58 2.56 1.59 1.25 1.24 0.88 0.88 0.87 0.86 0.85
C6H13
CN
NC
O
S
1.01 1.00 1.02 2.00 2.06 8.68
4.09
19.34 7.25
10 9 8 7 6 5 4 3 2 1 0
Chemical Shift (ppm)

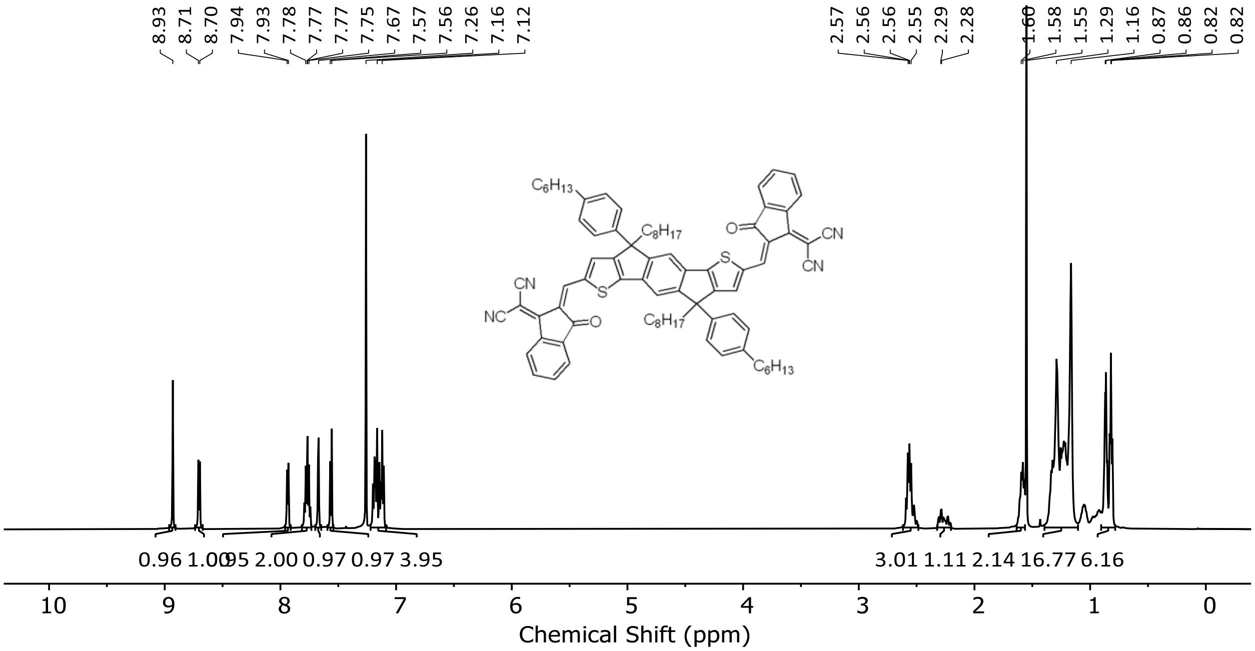
8.93 8.71 8.70 7.94 7.93 7.78 7.77 7.77 7.75 7.67 7.57 7.56 7.26 7.16 7.12
2.57 2.56 2.56 2.55 2.29 2.28 1.60 1.58 1.55 1.29 1.16 0.87 0.86 0.82 0.82
C6H13
C8H17
CN
NC
O
S
0.96 1.00 0.95 2.00 0.97 0.97 3.95
3.01 1.11 2.14 16.77 6.16
10 9 8 7 6 5 4 3 2 1 0
Chemical Shift (ppm)

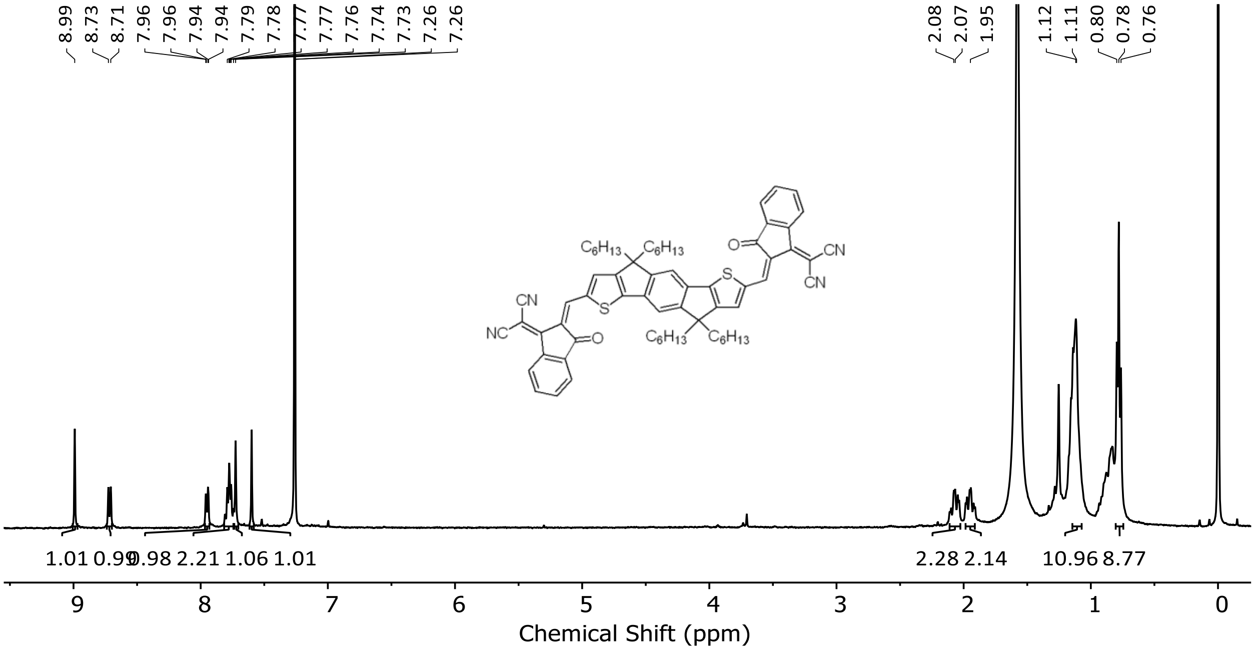
8.99
8.73
8.71
7.96
7.96
7.94
7.94
7.79
7.78
7.77
7.77
7.76
7.74
7.73
7.26
7.26
2.08
2.07
1.95
1.12
1.11
0.80
0.78
0.76
1.01 0.99 0.98 2.21 1.06 1.01
2.28 2.14
10.96 8.77
9 8 7 6 5 4 3 2 1 0
Chemical Shift (ppm)
C6H13
C6H13
CN
NC
CN
CN
O
O
S
S

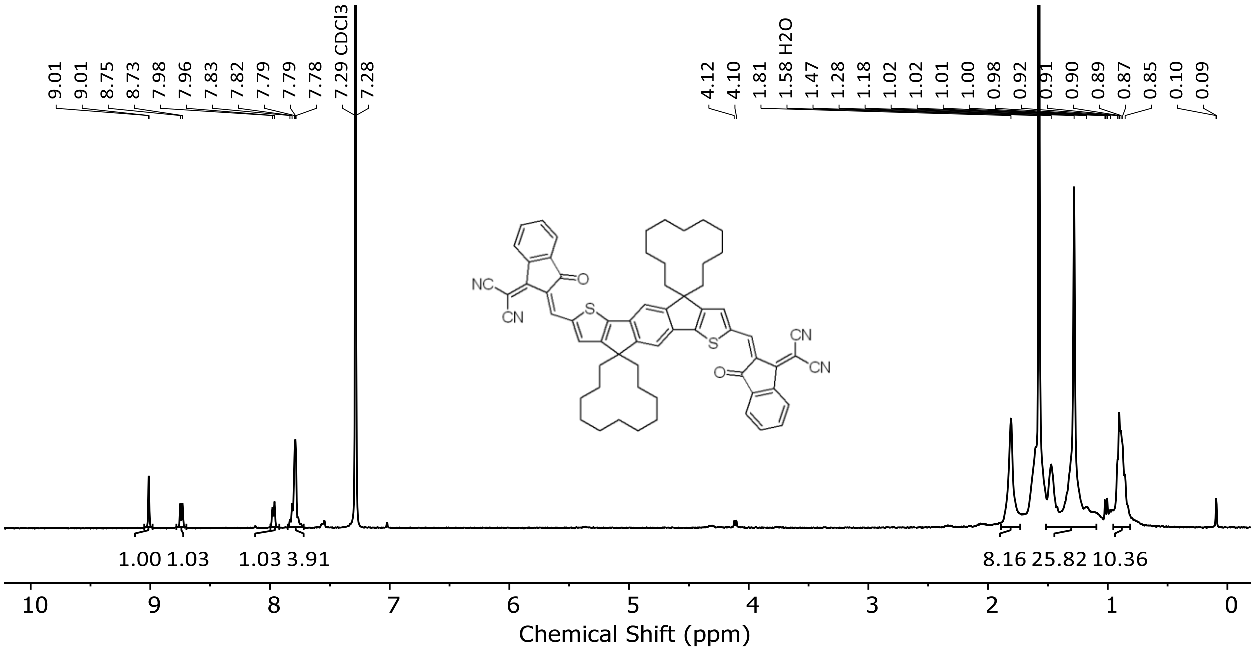
9.01
9.01
8.75
8.73
7.98
7.96
7.83
7.82
7.79
7.79
7.78
7.29 CDCl3
7.28
4.12
4.10
1.81
1.58 H2O
1.47
1.28
1.18
1.02
1.02
1.01
1.00
0.98
0.92
0.91
0.90
0.89
0.87
0.85
0.10
0.09
1.00 1.03
1.03 3.91
8.16 25.82 10.36
10 9 8 7 6 5 4 3 2 1 0
Chemical Shift (ppm)
NC
CN
CN
CN
O
O
S
S

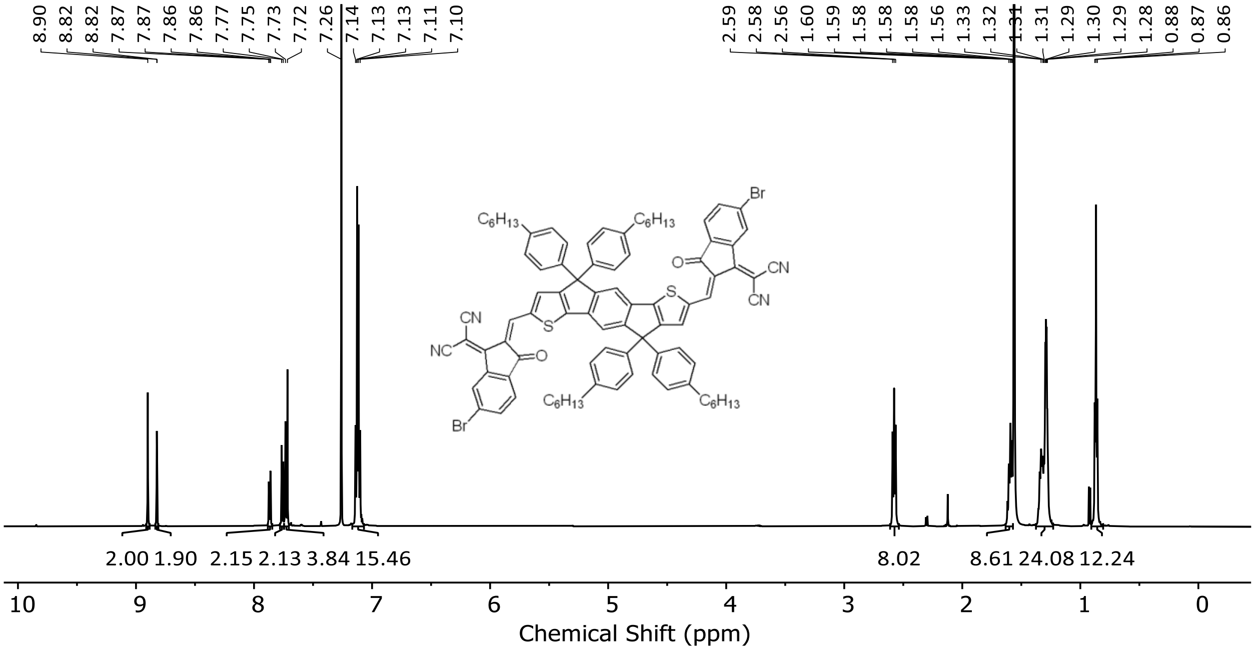
8.90 8.82 8.82 7.87 7.87 7.86 7.86 7.77 7.75 7.73 7.72 7.26 7.14 7.13 7.13 7.11 7.10
2.59 2.58 2.56 1.60 1.59 1.58 1.58 1.58 1.56 1.33 1.32 1.31 1.31 1.29 1.30 1.29 1.28 0.88 0.87 0.86
2.00 1.90 2.15 2.13 3.84 15.46
8.02 8.61 24.08 12.24
Chemical Shift (ppm)

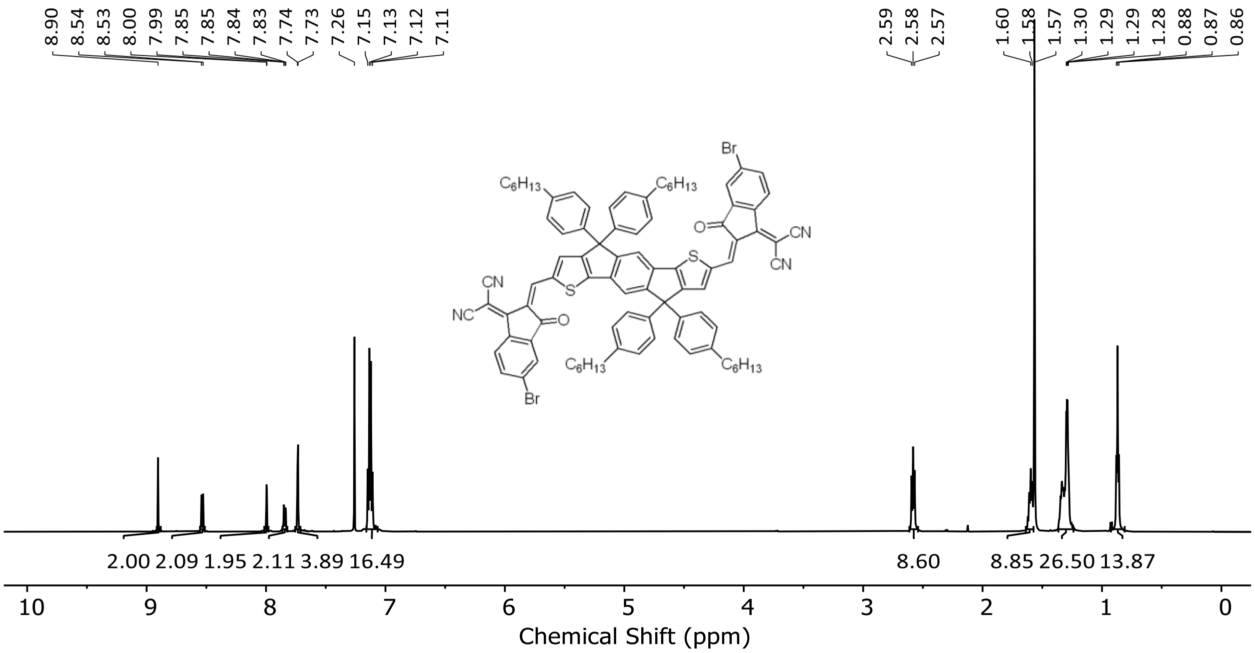
8.90 8.54 8.53 8.00 7.99 7.85 7.85 7.84 7.83 7.74 7.73 7.26 7.15 7.13 7.12 7.11
2.59 2.58 2.57 1.60 1.58 1.57 1.30 1.29 1.29 1.28 0.88 0.87 0.86
2.00 2.09 1.95 2.11 3.89 16.49
8.60 8.85 26.50 13.87
Chemical Shift (ppm)

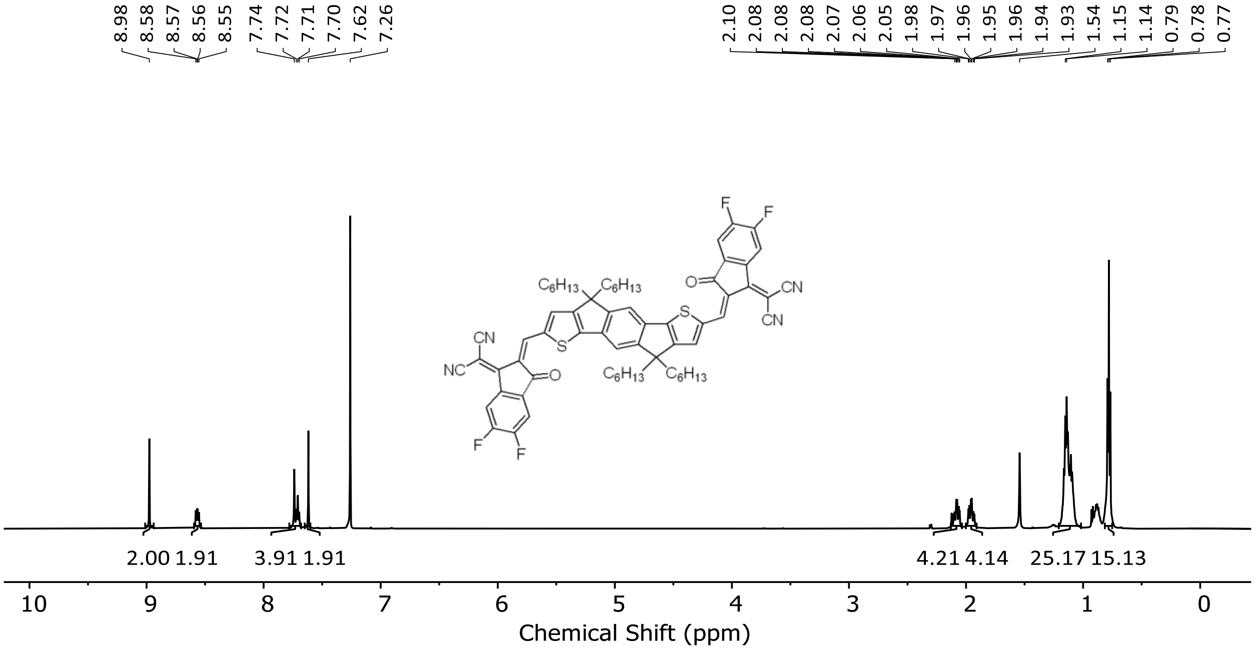
8.98
8.58
8.57
8.56
8.55
7.74
7.72
7.71
7.70
7.62
7.26
2.10
2.08
2.08
2.07
2.06
2.05
1.98
1.97
1.96
1.95
1.96
1.94
1.93
1.54
1.15
1.14
0.79
0.78
0.77
2.00 1.91
3.91 1.91
4.21 4.14
25.17 15.13
10 9 8 7 6 5 4 3 2 1 0
Chemical Shift (ppm)

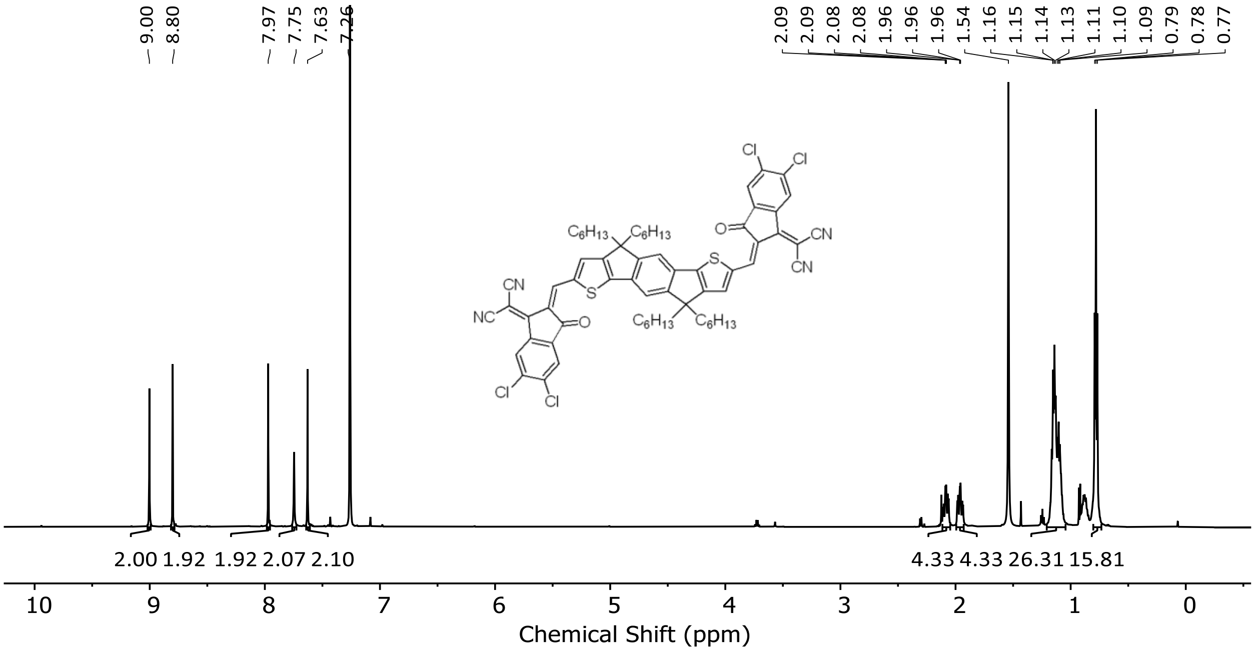
9.00
8.80
7.97
7.75
7.63
7.26
2.09
2.09
2.08
2.08
1.96
1.96
1.96
1.54
1.16
1.15
1.14
1.13
1.11
1.10
1.09
0.79
0.78
0.77
2.00 1.92 1.92 2.07 2.10
4.33 4.33 26.31 15.81
10 9 8 7 6 5 4 3 2 1 0
Chemical Shift (ppm)

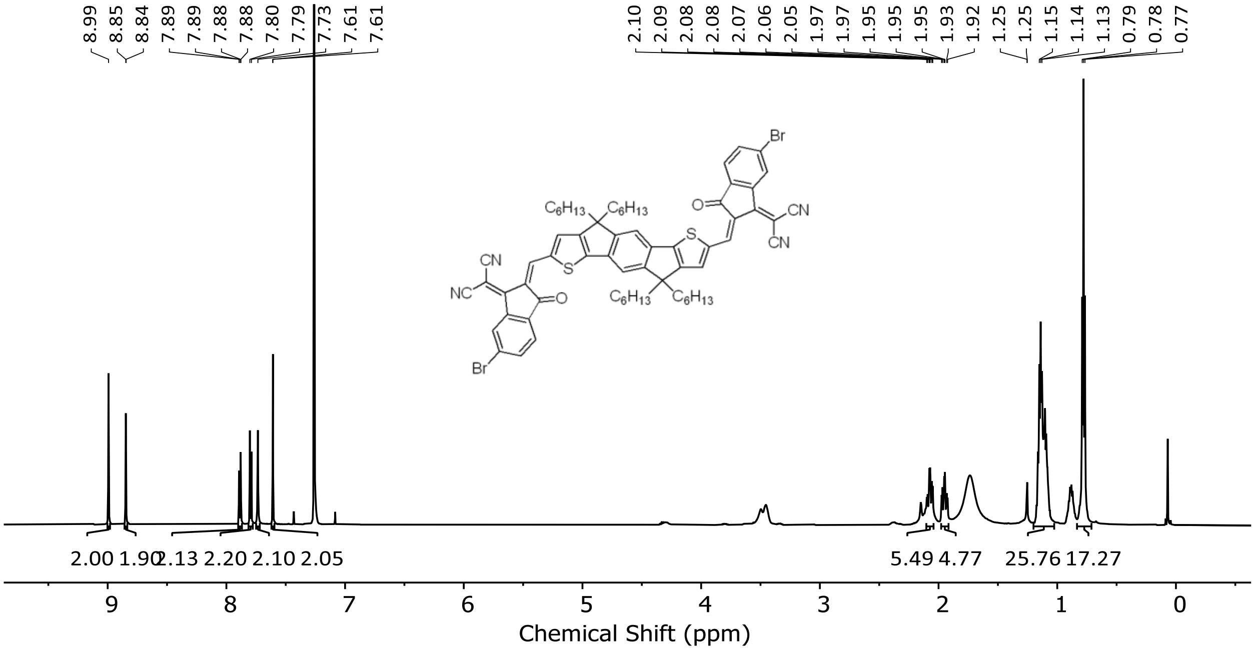
8.99 8.85 8.84 7.89 7.89 7.88 7.88 7.80 7.79 7.73 7.61 7.61
2.10 2.09 2.08 2.08 2.07 2.06 2.05 1.97 1.97 1.95 1.95 1.95 1.93 1.92 1.25 1.25 1.15 1.14 1.13 0.79 0.78 0.77
2.00 1.90 2.13 2.20 2.10 2.05
5.49 4.77 25.76 17.27
Chemical Shift (ppm)

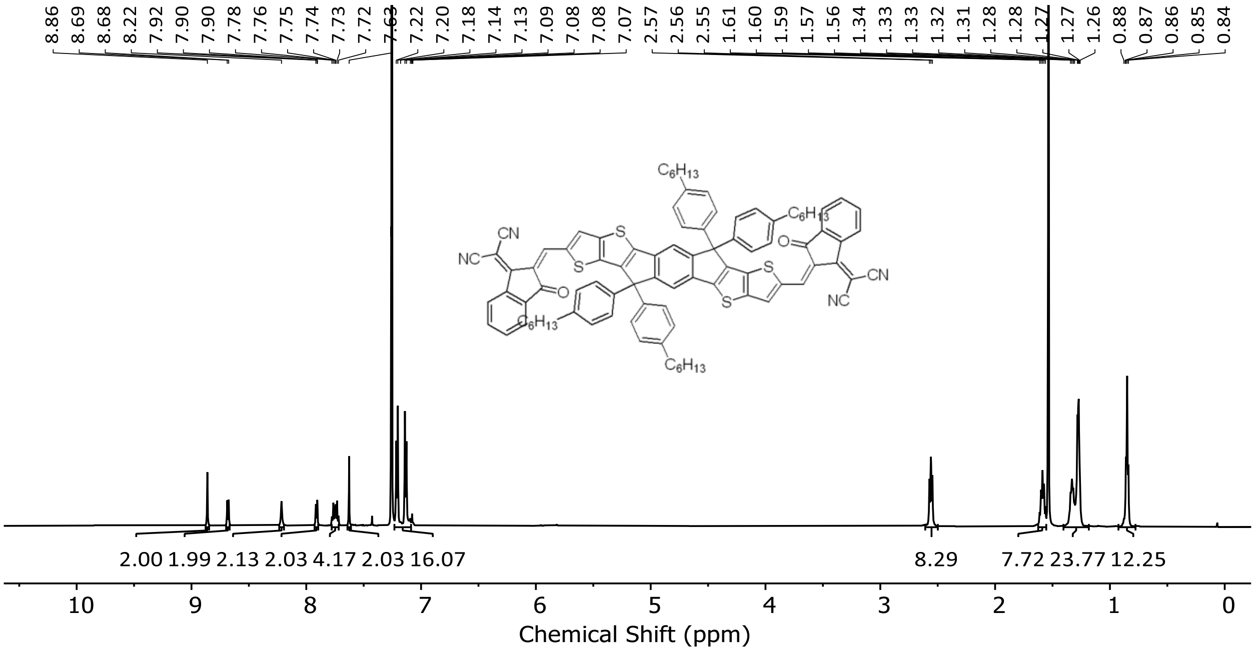
8.86 8.69 8.68 8.22 7.92 7.90 7.90 7.78 7.76 7.75 7.74 7.73 7.72 7.62 7.22 7.20 7.18 7.14 7.13 7.09 7.08 7.08 7.07
2.57 2.56 2.55 1.61 1.60 1.59 1.57 1.56 1.34 1.33 1.33 1.32 1.31 1.28 1.28 1.27 1.27 1.26 0.88 0.87 0.86 0.85 0.84
2.00 1.99 2.13 2.03 4.17 2.03 16.07
8.29 7.72 23.77 12.25
Chemical Shift (ppm)

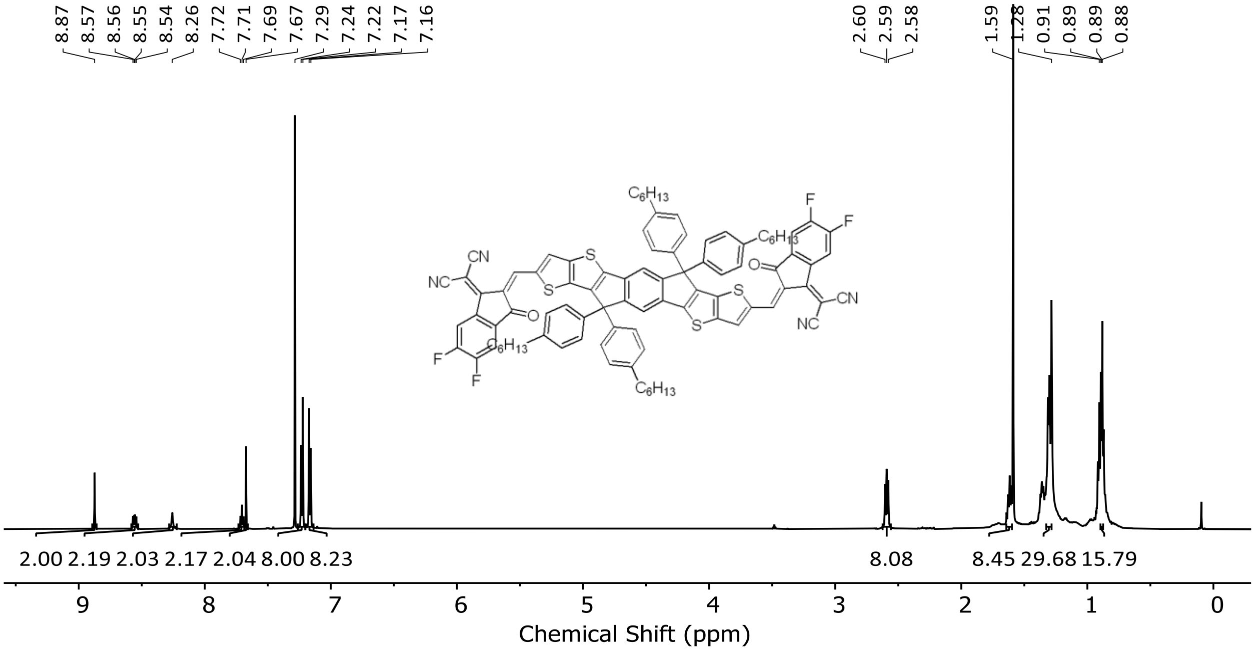
8.87 8.57 8.56 8.55 8.54 8.26 7.72 7.71 7.69 7.67 7.29 7.24 7.22 7.17 7.16
2.60 2.59 2.58
1.59 1.28 0.91 0.89 0.89 0.88
2.00 2.19 2.03 2.17 2.04 8.00 8.23
8.08
8.45 29.68 15.79
Chemical Shift (ppm)

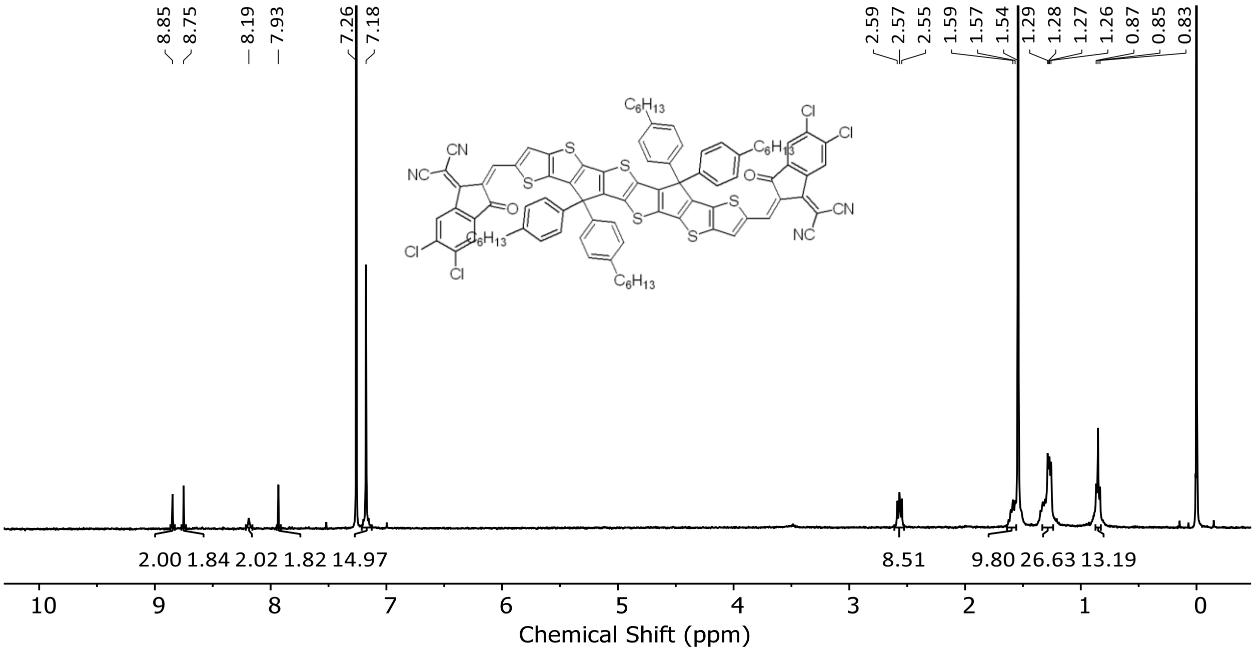
8.85 8.75 8.19 7.93 7.26 7.18
2.59 2.57 2.55 1.59 1.57 1.54 1.29 1.28 1.27 1.26 0.87 0.85 0.83
2.00 1.84 2.02 1.82 14.97
8.51
9.80 26.63 13.19
Chemical Shift (ppm)

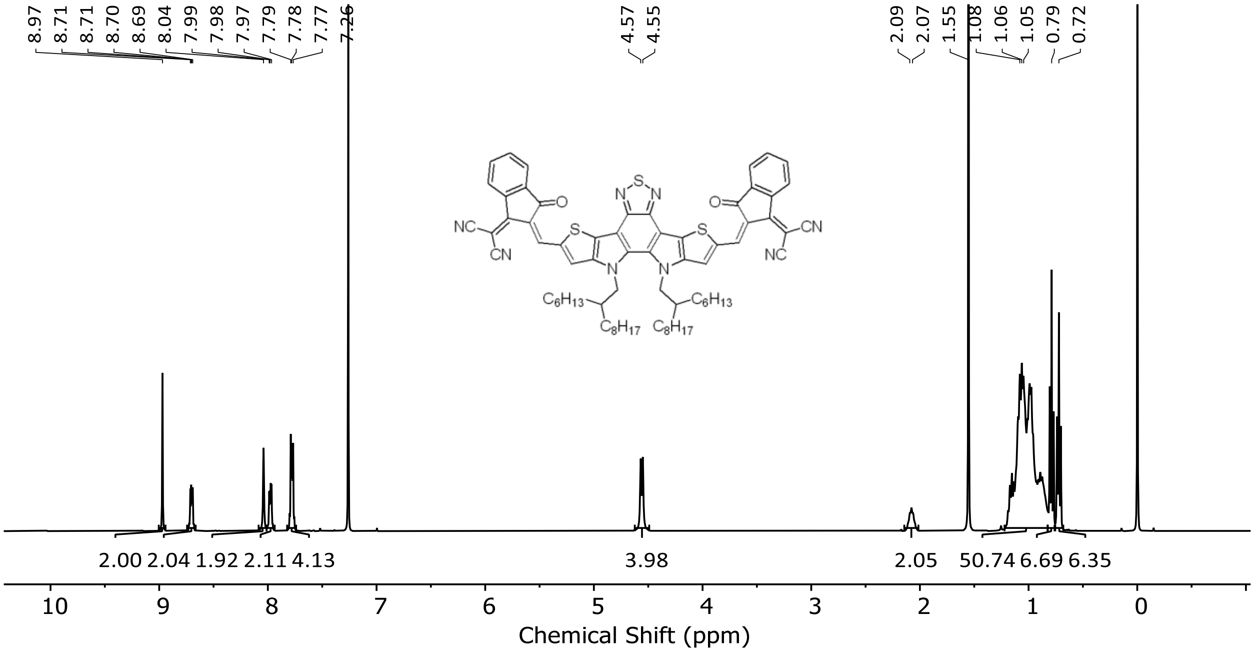
8.97 8.71 8.71 8.70 8.69 8.04 7.99 7.98 7.97 7.79 7.78 7.77 7.26
4.57 4.55
2.09 2.07 1.55 1.08 1.06 1.05 0.79 0.72
2.00 2.04 1.92 2.11 4.13
3.98
2.05 50.74 6.69 6.35
10 9 8 7 6 5 4 3 2 1 0
Chemical Shift (ppm)

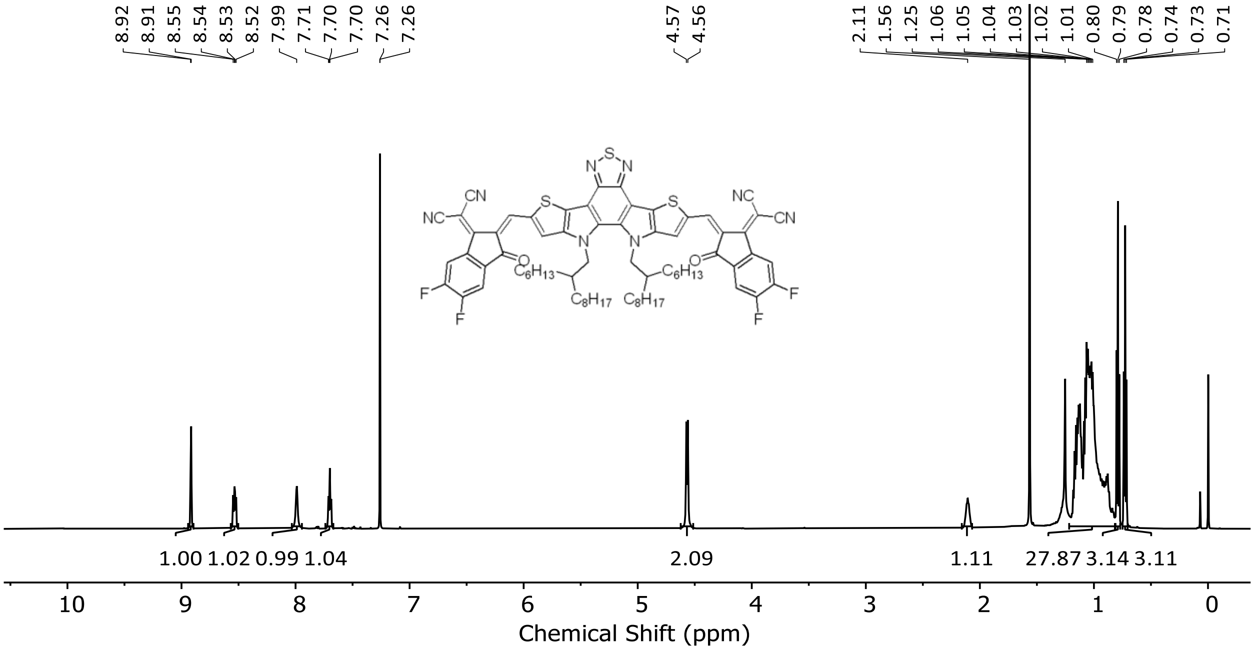
8.92 8.91 8.55 8.54 8.53 8.52 7.99 7.71 7.70 7.70 7.26 7.26
4.57 4.56
2.11 1.56 1.25 1.06 1.05 1.04 1.03 1.02 1.01 0.80 0.79 0.78 0.74 0.73 0.71
1.00 1.02 0.99 1.04
2.09
1.11 27.87 3.14 3.11
10 9 8 7 6 5 4 3 2 1 0
Chemical Shift (ppm)

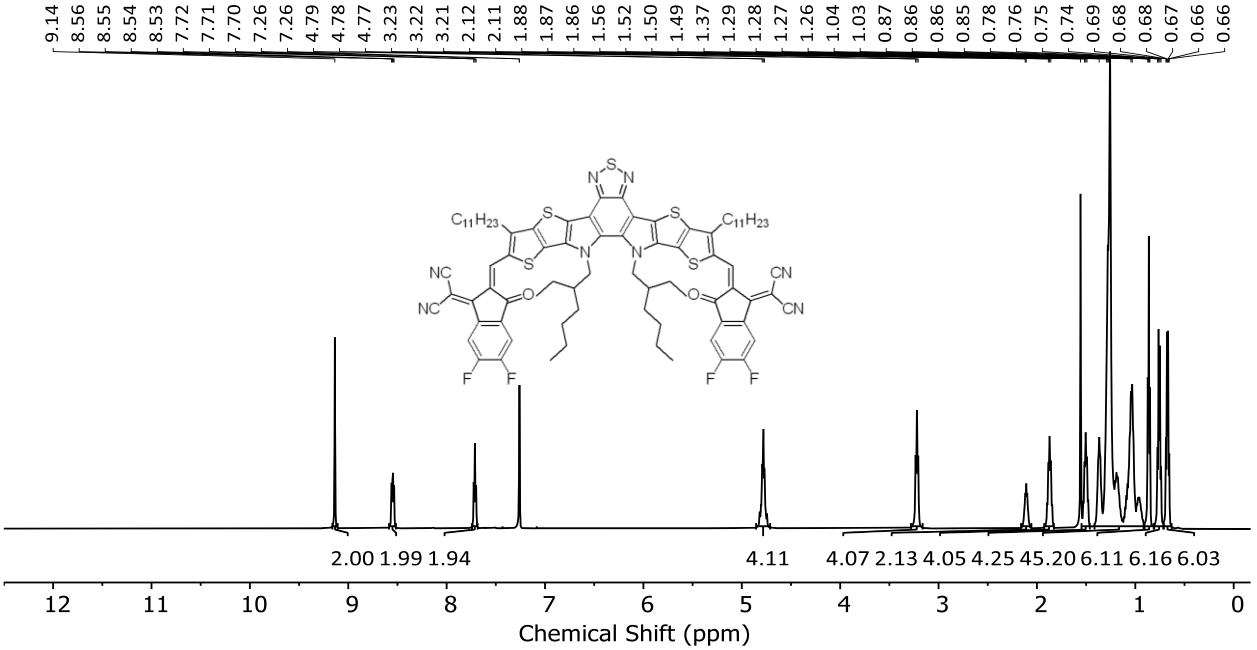

9.14 8.56 8.55 8.54 8.53 7.72 7.71 7.70 7.26 7.26 4.79 4.78 4.77 3.23 3.22 3.21 2.12 2.11 1.88 1.87 1.86 1.56 1.52 1.50 1.49 1.37 1.29 1.28 1.27 1.26 1.04 1.03 0.87 0.86 0.86 0.85 0.78 0.76 0.75 0.74 0.69 0.68 0.68 0.67 0.66 0.66
N S N
S
S
C11H23
C11H23
S
S
N
N
NC
CN
NC
CN
O
O
F F
F F
2.00 1.99 1.94 4.11 4.07 2.13 4.05 4.25 45.20 6.11 6.16 6.03
12 11 10 9 8 7 6 5 4 3 2 1 0
Chemical Shift (ppm)

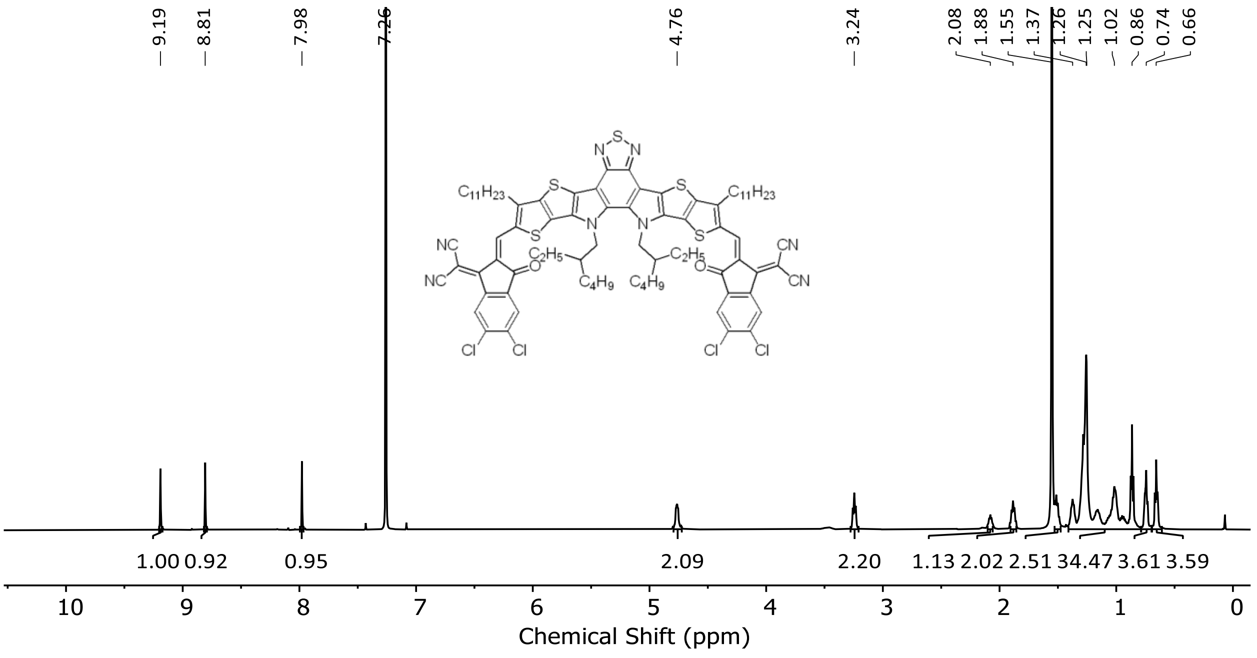

9.19 8.81 7.98 7.26 4.76 3.24 2.08 1.88 1.55 1.37 1.26 1.25 1.02 0.86 0.74 0.66
N S N
C11H23
C11H23
S
S
N
N
NC
CN
NC
CN
C2H5
C2H5
C4H9
C4H9
O
O
Cl Cl
Cl Cl
1.00 0.92 0.95 2.09 2.20 1.13 2.02 2.51 34.47 3.61 3.59
10 9 8 7 6 5 4 3 2 1 0
Chemical Shift (ppm)

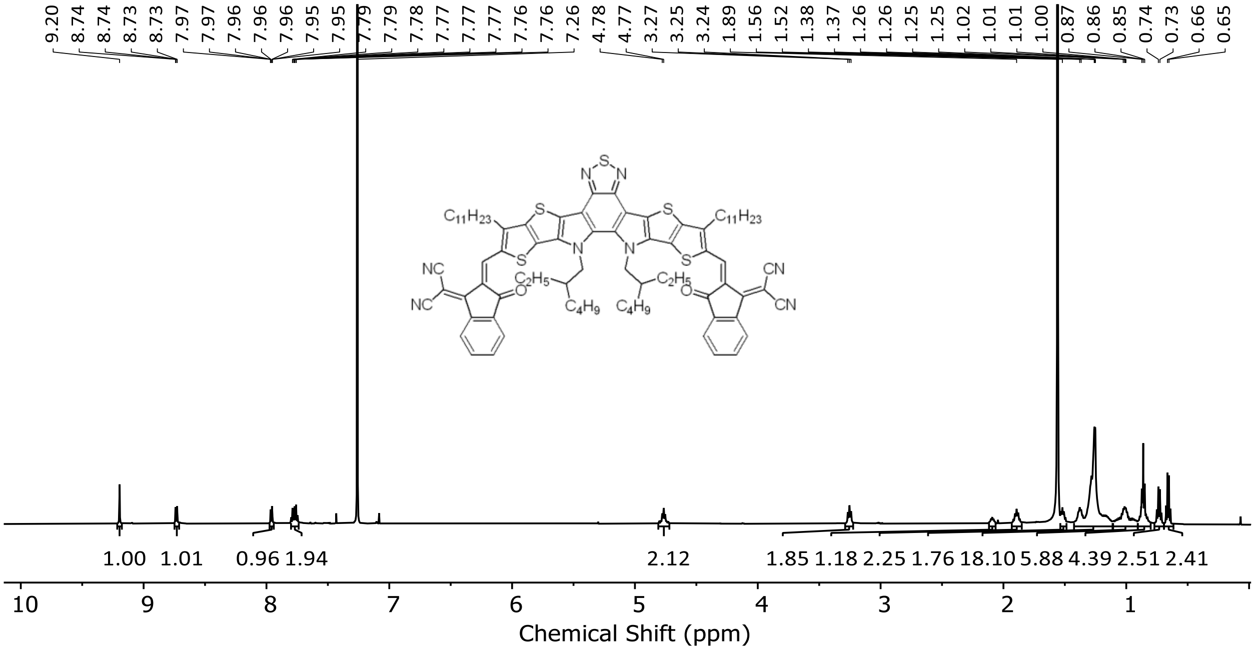
9.20 8.74 8.74 8.73 8.73 7.97 7.97 7.96 7.96 7.96 7.95 7.95 7.79 7.79 7.78 7.77 7.77 7.77 7.76 7.76 7.26 4.78 4.77 3.27 3.25 3.24 1.89 1.56 1.52 1.38 1.37 1.26 1.26 1.25 1.25 1.02 1.01 1.01 1.00 0.87 0.86 0.85 0.74 0.73 0.66 0.65
1.00 1.01 0.96 1.94 2.12 1.85 1.18 2.25 1.76 18.10 5.88 4.39 2.51 2.41
10 9 8 7 6 5 4 3 2 1
Chemical Shift (ppm)

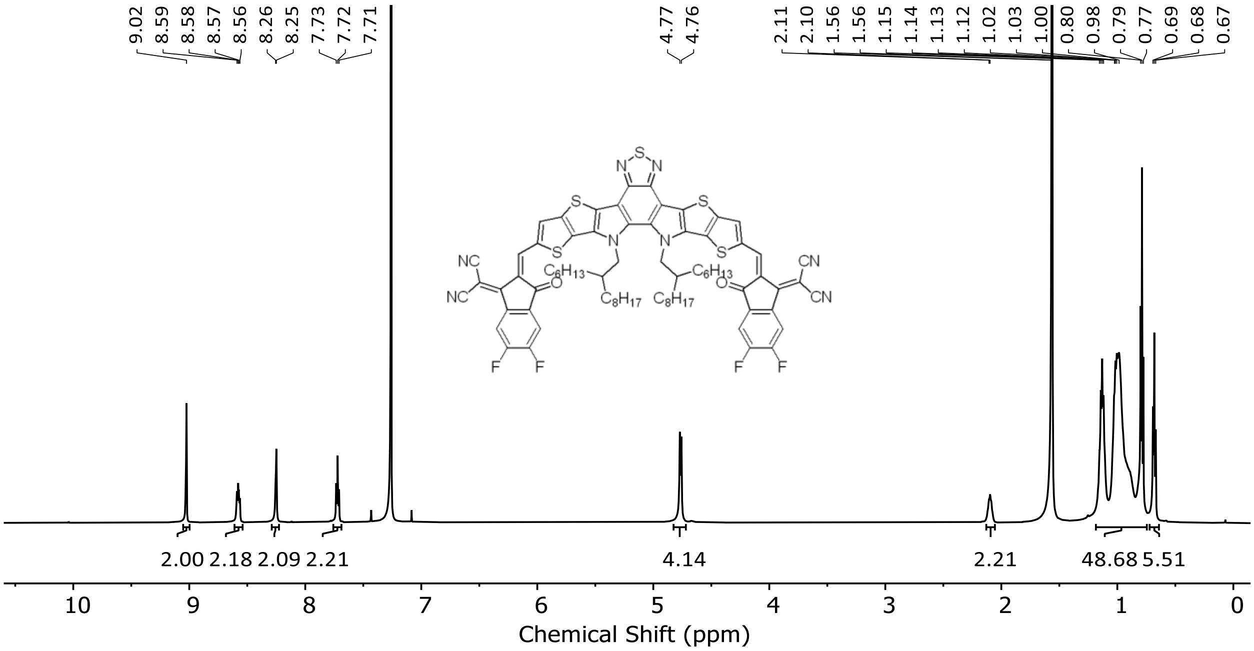
9.02 8.59 8.58 8.57 8.56 8.26 8.25 7.73 7.72 7.71 4.77 4.76 2.11 2.10 1.56 1.56 1.15 1.14 1.13 1.12 1.02 1.03 1.00 0.80 0.98 0.79 0.77 0.69 0.68 0.67
2.00 2.18 2.09 2.21 4.14 2.21 48.68 5.51
10 9 8 7 6 5 4 3 2 1 0
Chemical Shift (ppm)

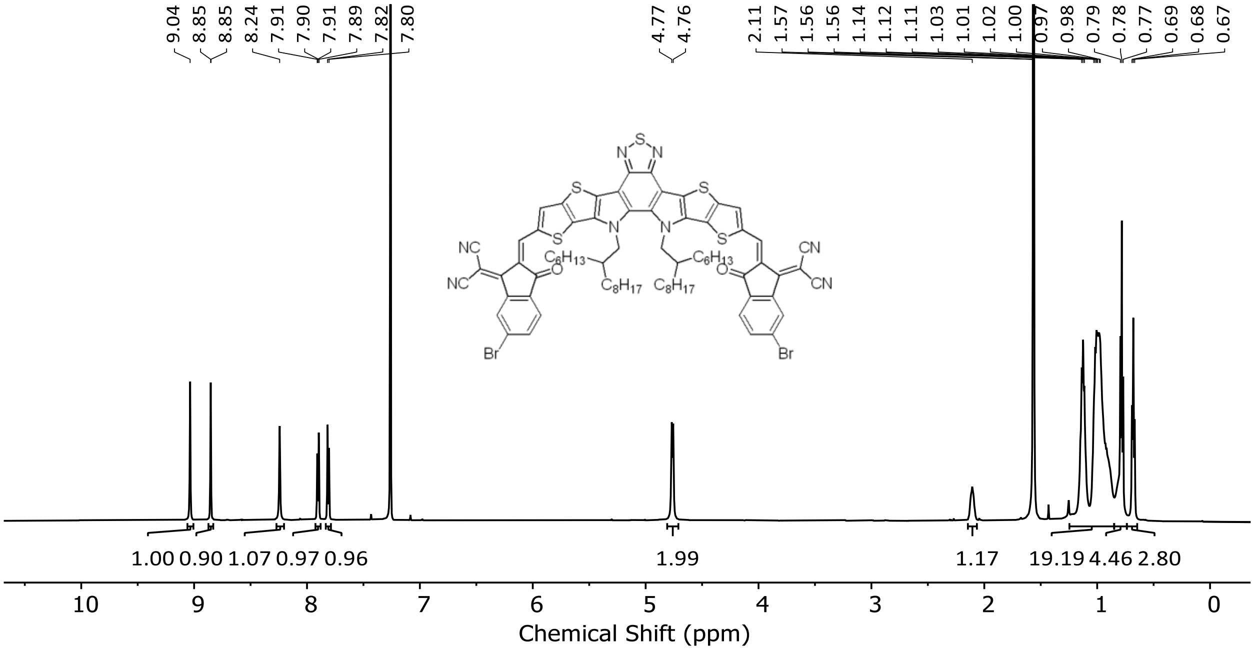
9.04 8.85 8.85 8.24 7.91 7.90 7.91 7.89 7.82 7.80
4.77 4.76
2.11 1.57 1.56 1.56 1.14 1.12 1.11 1.03 1.01 1.02 1.00 0.97 0.98 0.79 0.78 0.77 0.69 0.68 0.67
N S N
S S
S S
N N
NC CN
NC CN
O O
C_6H_{13} C_6H_{13}
C_8H_{17} C_8H_{17}
Br Br
1.00 0.90 1.07 0.97 0.96
1.99
1.17
19.19 4.46 2.80
10 9 8 7 6 5 4 3 2 1 0
Chemical Shift (ppm)

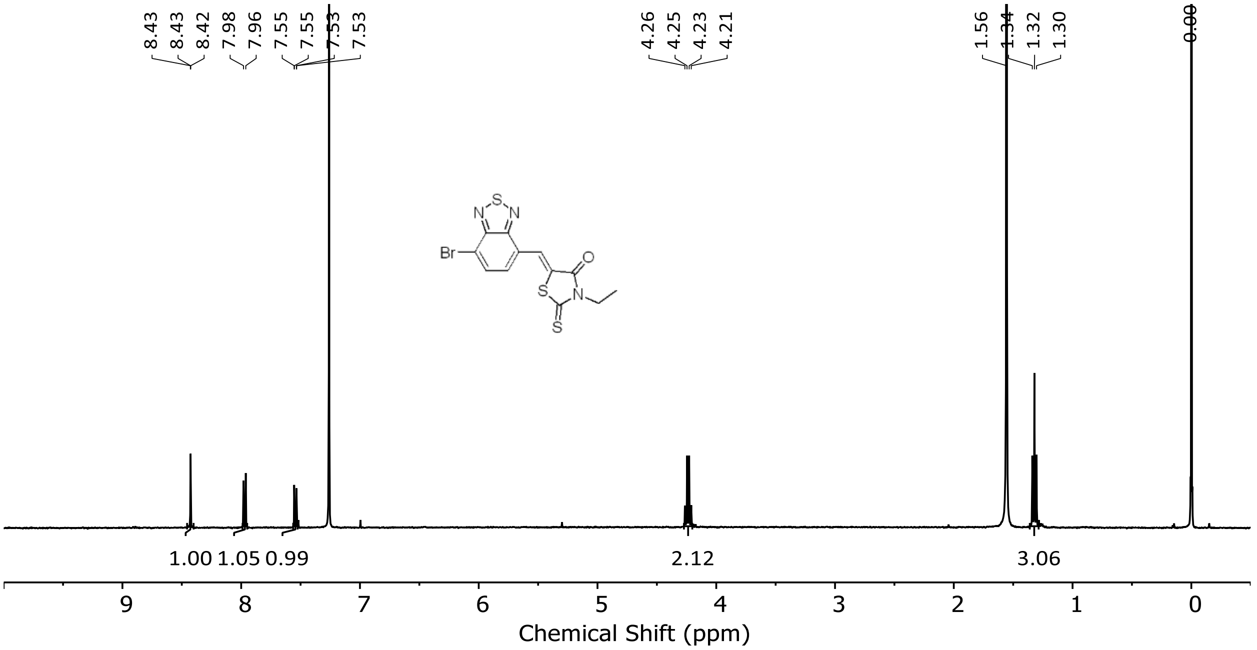
8.43 8.43 8.42 7.98 7.96 7.55 7.55 7.53 7.53
4.26 4.25 4.23 4.21
1.56 1.34 1.32 1.30
0.00
N S N
Br
O
S N
S
1.00 1.05 0.99
2.12
3.06
9 8 7 6 5 4 3 2 1 0
Chemical Shift (ppm)

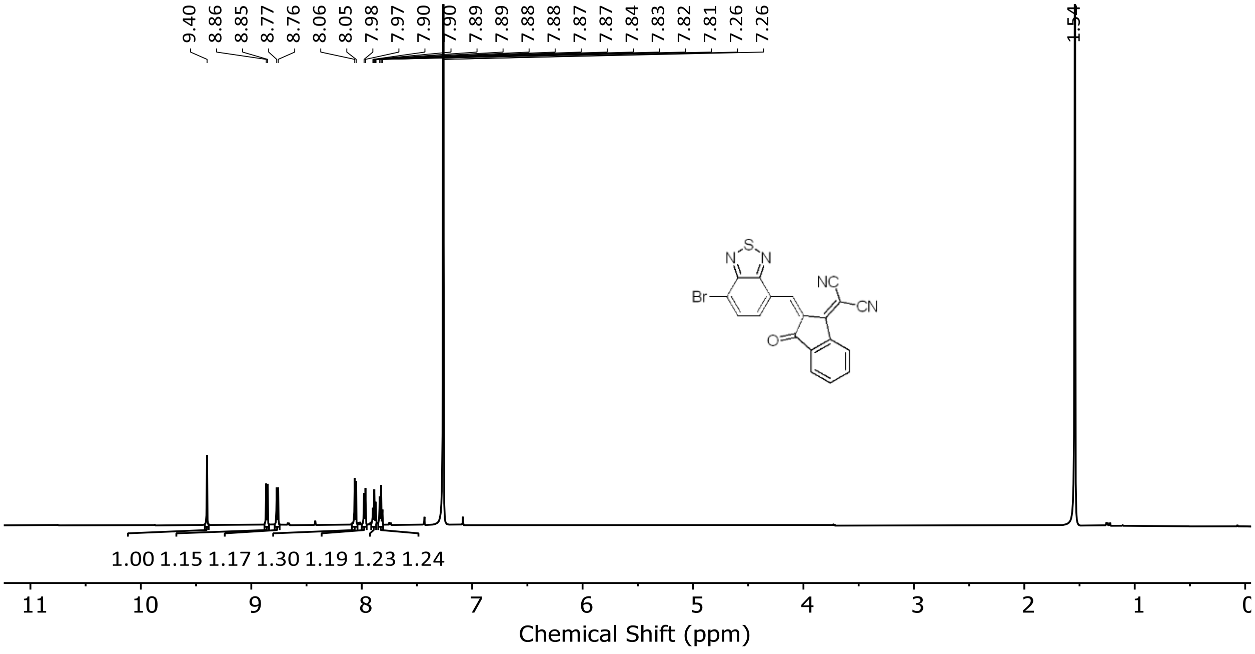
9.40 8.86 8.85 8.77 8.76 8.06 8.05 7.98 7.97 7.90 7.90 7.89 7.89 7.88 7.88 7.87 7.87 7.84 7.83 7.82 7.81 7.26 7.26
1.54
1.00 1.15 1.17 1.30 1.19 1.23 1.24
11 10 9 8 7 6 5 4 3 2 1 0
Chemical Shift (ppm)
Br
N
S
N
NC
CN
O

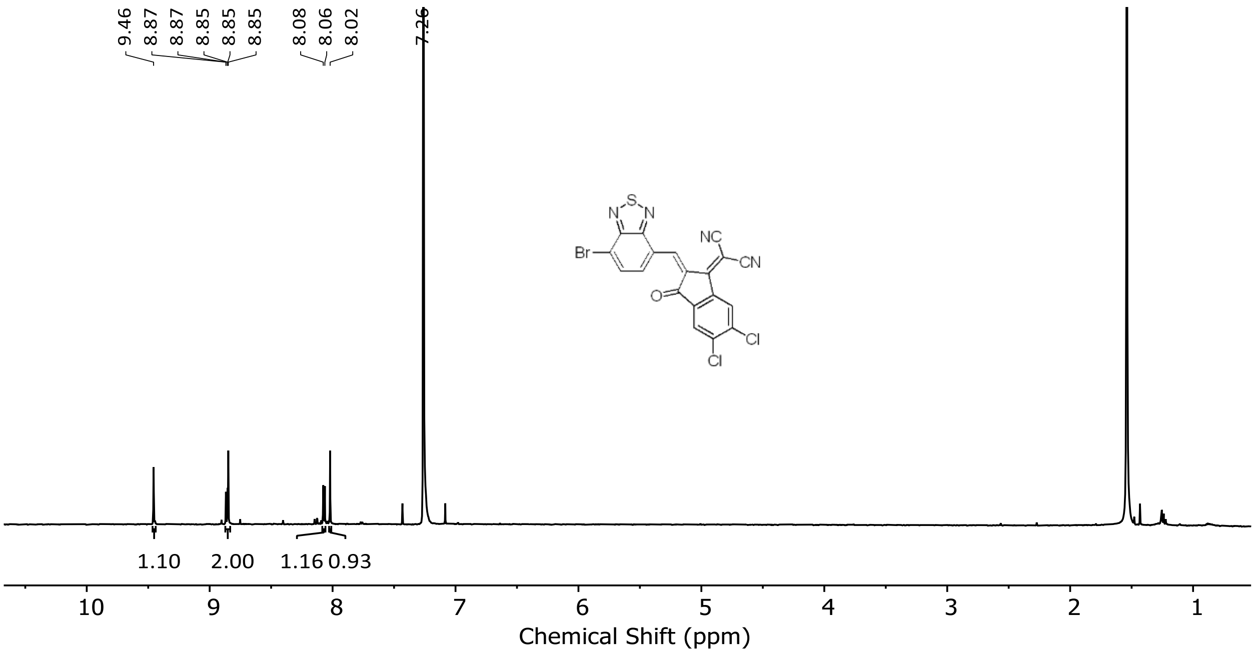
9.46 8.87 8.87 8.85 8.85 8.85 8.08 8.06 8.02
7.26
1.10 2.00 1.16 0.93
10 9 8 7 6 5 4 3 2 1
Chemical Shift (ppm)
Br
N
S
N
NC
CN
O
Cl
Cl

Cartesian coordinates for the optimized transition state structures of A-E pathways

1. Pathway A

C	6.49775	-1.16065	-0.89469
C	5.35625	-1.42581	-1.58041
C	4.22653	-0.70592	-1.05999
C	4.54649	0.05731	0.04154
S	6.21738	-0.09838	0.42638
H	7.49246	-1.52923	-1.09251
H	5.31994	-2.05928	-2.45554
C	3.64966	0.77440	0.97929
C	2.92804	0.03223	1.92103
C	3.54782	2.16834	0.97389
C	2.09865	0.67706	2.83468
H	3.01855	-1.04791	1.93311
C	2.70591	2.80812	1.87763
H	4.11780	2.74644	0.25654
C	1.97886	2.06529	2.80631
H	1.53482	0.09605	3.55420
H	2.61235	3.88641	1.85272
H	1.32126	2.56604	3.50644
C	2.91243	-0.80316	-1.69882
C	2.36551	-2.17702	-1.87879
H	1.47324	-2.14594	-2.50173
H	3.12078	-2.76084	-2.41694
C	2.17279	0.31747	-2.09197
H	1.34017	0.07248	-2.75576
H	1.50188	0.33143	-1.05632
O	0.53717	0.81320	0.01596
H	1.11030	1.04116	0.75940
C	2.09355	-2.84538	-0.51822
H	1.62786	-3.81686	-0.68589
H	3.02371	-2.99423	0.03235
H	1.41267	-2.23552	0.07839
C	2.76044	1.70774	-2.25040
H	2.68348	2.00448	-3.29727
H	2.20878	2.43364	-1.65086
H	3.80985	1.73574	-1.95881
S	-1.29248	-0.92163	2.21352
O	-0.17199	-0.77912	1.30551
O	-1.08087	-0.65940	3.60636
C	-1.78667	-2.68640	2.00758
F	-2.09402	-2.85307	0.72628
F	-0.78544	-3.48342	2.33085
F	-2.83964	-2.95976	2.75451
O	-2.34180	-0.21241	1.51399
S	-0.72245	2.11793	-0.25547
O	0.27141	3.16677	-0.37201
O	-1.15532	1.12620	-1.24169
C	-2.25094	3.13791	-0.47481
F	-2.34043	4.10309	0.43450
F	-2.30120	3.68881	-1.68380
F	-3.30640	2.32624	-0.33496
O	-1.23604	1.56825	1.03116
S	-1.20633	-1.61083	-2.13332
O	-1.42572	-3.03839	-2.18078
O	-0.69159	-0.95774	-3.31824

C	-2.86750	-0.83801	-1.76041
F	-2.57263	-0.25487	-0.52353
F	-3.25275	0.10608	-2.56611
F	-3.82349	-1.70043	-1.55235
O	-0.58904	-1.13138	-0.86377
Yb	-1.16238	0.19057	0.23199

2. Pathway B

C	6.06281	-0.98238	1.01908
C	5.06307	-1.75297	0.51177
C	3.95998	-1.00135	0.00384
C	4.17294	0.36360	0.14624
S	5.70603	0.68471	0.90580
H	6.99332	-1.30708	1.45897
H	5.10768	-2.83308	0.50510
C	3.39016	1.51208	-0.29351
C	3.53809	2.80815	0.23630
C	2.41202	1.36528	-1.27790
C	2.83805	3.85980	-0.32692
H	4.21828	2.98649	1.06133
C	1.79878	2.41111	-1.95695
H	1.78583	0.17973	-0.97559
C	1.97265	3.67446	-1.42116
H	2.97657	4.85769	0.07135
H	1.12693	2.23144	-2.78594
H	1.43422	4.51820	-1.83470
C	2.77293	-1.75146	-0.59043
C	2.41235	-2.99745	0.23270
H	1.45304	-3.35389	-0.15280
H	3.14330	-3.78005	0.02151
C	2.99017	-2.07772	-2.07702
H	2.11640	-2.64985	-2.40156
H	2.97433	-1.12924	-2.62563
O	1.56243	-0.93326	-0.60061
H	1.02036	-0.95062	0.22469
C	2.31790	-2.76601	1.74243
H	1.80764	-3.60725	2.21123
H	3.30250	-2.66224	2.19825
H	1.75420	-1.86205	1.99283
C	4.27463	-2.83071	-2.40725
H	5.15980	-2.23484	-2.18008
H	4.34652	-3.77542	-1.86426
H	4.29115	-3.06330	-3.47239
S	-1.19863	1.56339	0.72963
O	0.06061	0.96905	0.45811
O	-1.85636	1.99691	-0.44597
C	-0.81527	3.06506	1.72174
F	-0.15374	2.72275	2.81889
F	-0.07044	3.90027	1.00962
F	-1.94295	3.67330	2.06406
O	-2.05816	0.77118	1.53797
S	-1.11799	-0.57534	-2.18630
O	-0.57187	-1.18931	-3.35714
O	-1.40200	-1.35008	-1.00349
C	-2.80790	0.06520	-2.39391
F	-2.92934	0.33950	-1.05716

F	-2.92169	1.17480	-3.06770
F	-3.72316	-0.79798	-2.73921
O	-0.55034	0.60756	-1.60610
S	-1.04640	-2.18856	1.80286
O	-0.88020	-2.04722	3.23553
O	-0.72706	-3.47304	1.20901
C	-2.83957	-1.87312	1.43270
F	-2.74767	-0.87735	0.50967
F	-3.45741	-2.88864	0.87418
F	-3.53190	-1.42920	2.45412
O	-0.56724	-1.01669	1.01560
Yb	-1.49081	0.18716	-0.09480

3. Pathway C

C	-3.10910	-2.68185	-0.17347
C	-1.79306	-2.73615	0.14059
C	-1.19033	-1.43144	0.25245
C	-2.09204	-0.41718	-0.04485
S	-3.63758	-1.06181	-0.42648
H	-3.80578	-3.49937	-0.27556
H	-1.27296	-3.66044	0.34867
C	-1.84986	1.02226	-0.21888
C	-0.75594	1.45579	-0.97252
C	-2.70678	1.96441	0.35866
C	-0.49930	2.81440	-1.11169
H	-0.12590	0.72268	-1.46488
C	-2.44832	3.32114	0.21377
H	-3.55283	1.63008	0.94875
C	-1.33994	3.74840	-0.51267
H	0.34737	3.14784	-1.69972
H	-3.10816	4.04526	0.67541
H	-1.13732	4.80677	-0.62079
C	0.18330	-1.28521	0.69246
C	1.16712	-2.30514	0.20441
H	2.16168	-2.04509	0.56890
H	0.89682	-3.26472	0.66128
C	0.66878	-0.25485	1.51793
H	1.59210	-0.56588	2.01541
H	1.30663	0.29422	0.62425
O	2.40832	0.93649	-0.19798
H	2.24213	1.31687	-1.06510
C	1.17669	-2.43506	-1.32473
H	1.94932	-3.14540	-1.61839
H	0.21685	-2.78249	-1.70773
H	1.41982	-1.47488	-1.78238
C	-0.19179	0.70879	2.31238
H	-1.21040	0.33454	2.43011
H	0.24311	0.83847	3.30300
H	-0.24893	1.69171	1.84104
B	3.71007	0.25789	-0.12045
F	3.79976	-0.76322	-1.08552
F	3.79383	-0.30696	1.16510
F	4.75689	1.16880	-0.32130

4. Pathway D

C	3.93514	-1.78813	-0.17528

C	2.63369	-2.15587	-0.33915
C	1.76256	-1.03391	-0.20726
C	2.43295	0.12055	0.14011
S	4.13469	-0.11449	0.21507
H	4.81283	-2.40914	-0.27430
H	2.32276	-3.15762	-0.60014
C	1.58079	1.22780	0.45876
C	0.25165	0.82024	0.78552
C	1.87864	2.58489	0.28880
C	-0.79482	1.77354	0.75393
H	0.12093	-0.00540	1.48377
C	0.84488	3.50073	0.32193
H	2.88986	2.89619	0.05729
C	-0.49218	3.09443	0.51106
H	-1.80287	1.44725	0.97443
H	1.06022	4.54963	0.15681
H	-1.27862	3.83847	0.50795
C	0.30594	-0.95267	-0.36316
C	-0.47572	-2.09356	0.23023
H	-1.53679	-1.94359	0.03667
H	-0.15967	-2.95426	-0.37950
C	-0.30047	-0.40821	-1.63728
H	-0.32356	-1.30617	-2.27582
H	-1.34502	-0.15806	-1.43646
O	-2.98671	-0.37610	1.19174
H	-3.33524	-1.05288	1.77644
C	-0.22144	-2.39865	1.70363
H	-0.62863	-3.37786	1.95554
H	0.84450	-2.40586	1.94361
H	-0.72538	-1.66481	2.33732
C	0.41285	0.71374	-2.37018
H	1.47303	0.49857	-2.51836
H	-0.04697	0.84571	-3.35005
H	0.31867	1.65908	-1.83360
B	-3.76533	-0.29449	-0.01538
F	-3.54796	-1.41972	-0.86412
F	-3.36623	0.86406	-0.72367
F	-5.15486	-0.22386	0.24674

5. Pathway E (last step)

C	-2.49039	-2.58822	-0.59039
C	-2.60953	-1.69825	0.44525
C	-1.72909	-0.60459	0.27470
C	-0.96972	-0.70589	-0.87291
S	-1.31741	-2.13338	-1.77033
H	-3.03894	-3.50805	-0.73201
H	-3.29586	-1.83197	1.27002
C	-0.10249	0.41534	-0.99938
C	-0.19808	1.17390	0.24128
C	0.72456	0.85423	-2.03447
C	0.27400	2.54341	0.22272
H	0.82051	0.75080	0.71210
C	1.29445	2.10651	-1.91779
H	0.87627	0.25506	-2.92266
C	1.05217	2.96789	-0.81519
H	0.10573	3.17313	1.09040

H	1.93173	2.46502	-2.71830
H	1.48862	3.95764	-0.81020
C	-1.43241	0.66401	1.03100
C	-1.18213	0.43710	2.53185
H	-0.77636	1.36662	2.94582
H	-2.15380	0.28639	3.01253
C	-2.59691	1.67450	0.85144
H	-3.47829	1.24297	1.33457
H	-2.33790	2.57724	1.41473
O	2.38020	0.38731	1.04475
H	2.99342	1.12681	1.01515
C	-0.26083	-0.73473	2.86494
H	-0.08900	-0.78008	3.94174
H	-0.70548	-1.68352	2.55741
H	0.70910	-0.64139	2.37417
C	-2.92692	2.03572	-0.59332
H	-3.24809	1.15892	-1.16007
H	-3.73449	2.76869	-0.61898
H	-2.06912	2.47731	-1.10999
B	2.89876	-0.77964	0.33695
F	3.54871	-0.39675	-0.85585
F	1.79884	-1.60632	0.03792
F	3.81524	-1.49397	1.13435

REFERENCES AND NOTES

1. Y. Lin, J. Wang, Z. G. Zhang, H. Bai, Y. Li, D. Zhu, X. Zhan, An electron acceptor challenging fullerenes for efficient polymer solar cells. *Adv. Mater.* **27**, 1170–1174 (2015).

2. J. Yuan, Y. Zhang, L. Zhou, G. Zhang, H.-L. Yip, T.-K. Lau, X. Lu, C. Zhu, H. Peng, P. A. Johnson, M. Leclerc, Y. Cao, J. Ulanski, Y. Li, Y. Zou, Single-junction organic solar cell with over 15% efficiency using fused-ring acceptor with electron-deficient core. *Joule* **3**, 1140–1151 (2019).

3. A. Wadsworth, M. Moser, A. Marks, M. S. Little, N. Gasparini, C. J. Brabec, D. Baran, I. McCulloch, Critical review of the molecular design progress in non-fullerene electron acceptors towards commercially viable organic solar cells. *Chem. Rev. Soc.* **48**, 1596–1625 (2019).

4. M. Kim, S. U. Ryu, S. A. Park, Y.-J. Pu, T. Park, Designs and understanding of small molecule-based non-fullerene acceptors for realizing commercially viable organic photovoltaics. *Chem. Sci.* **12**, 14004–14023 (2021).

5. J. Wang, X. Zhan, From perylene diimide polymers to fused-ring electron acceptors: A 15-year exploration journey of nonfullerene acceptors. *Chin. J. Chem.* **40**, 1592–1607 (2022).

6. Y. Lin, Q. He, F. Zhao, L. Huo, J. Mai, X. Lu, C.-J. Su, T. Li, J. Wang, J. Zhu, Y. Sun, C. Wang, X. Zhan, A facile planar fused-ring electron acceptor for as-cast polymer solar cells with 8.71% efficiency. *J. Am. Chem. Soc.* **138**, 2973–2976 (2016).

7. J. J. Rech, N. Bauer, D. Dirkes, J. Kaplan, Z. Peng, H. Zhang, L. Ye, S. Liu, F. Gao, H. Ade, W. You, The crucial role of end group planarity for fused-ring electron acceptors in organic solar cells. *Mater. Chem. Frontiers* **3**, 1642–1652 (2019).

8. S.-H. Chan, C.-P. Chen, T.-C. Chao, C. Ting, C.-S. Lin, B.-T. Ko, Synthesis, characterization, and photovoltaic properties of novel semiconducting polymers with Thiophene−Phenylene−Thiophene (TPT) as coplanar units. *Macromolecules* **41**, 5519–5526 (2008).

9. S. Feng, C. Zhang, Y. Liu, Z. Bi, Z. Zhang, X. Xu, W. Ma, Z. Bo, Fused-ring acceptors with asymmetric side chains for high-performance thick-film organic solar cells. *Adv. Mater.* **29**, 1703527 (2017).

10. Z. Fei, F. D. Eisner, X. Jiao, M. Azzouzi, J. A. Röhr, Y. Han, M. Shahid, A. S. R. Chesman, C. D. Easton, C. R. McNeill, T. D. Anthopoulos, J. Nelson, M. Heeney, An alkylated indacenodithieno[3,2-b]thiophene-based nonfullerene acceptor with high crystallinity exhibiting single junction solar cell efficiencies greater than 13% with low voltage losses. *Adv. Mater.* **30**, 1705209 (2018).

11. W. Zhang, J. Smith, S. E. Watkins, R. Gysel, M. McGehee, A. Salleo, J. Kirkpatrick, S. Ashraf, T. Anthopoulos, M. Heeney, I. McCulloch, Indacenodithiophene semiconducting polymers for high-performance, air-stable transistors. *J. Am. Chem. Soc.* **132**, 11437–11439 (2010).

12. S. Y. Cho, A. C. Grimsdale, D. J. Jones, S. E. Watkins, A. B. Holmes, Polyfluorenes without monoalkylfluorene defects. *J. Am. Chem. Soc.* **129**, 11910–11911 (2007).

13. Y.-J. Cheng, C.-H. Chen, Y.-J. Ho, S.-W. Chang, H. A. Witek, C.-S. Hsu, Thieno[3,2-b]pyrrolo donor fused with benzothiadiazolo, benzoselenadiazolo and quinoxalino acceptors: Synthesis, characterization, and molecular properties. *Org. Lett.* **13**, 5484–5487 (2011).

14. L. Feng, J. Yuan, Z. Zhang, H. Peng, Z. G. Zhang, S. Xu, Y. Liu, Y. Li, Y. Zou, Thieno[3,2-b]pyrrolo-fused pentacyclic benzotriazole-based acceptor for efficient organic photovoltaics. *ACS Appl. Mater. Interfaces* **9**, 31985–31992 (2017).

15. A. W. Freeman, M. Urvoy, M. E. Criswell, Triphenylphosphine-mediated reductive cyclization of 2-nitrobiphenyls: A practical and convenient synthesis of carbazoles. *J. Org. Chem* **70**, 5014–5019 (2005).

16. R. Sanz, J. Escribano, M. R. Pedrosa, R. Aguado, F. J. Arnáiz, Dioxomolybdenum(VI)-catalyzed reductive cyclization of nitroaromatics. synthesis of carbazoles and indoles. *Adv. Synth. Catal.* **349**, 713–718(2007).

17. F. Ferretti, D. R. Ramadan, F. Ragaini, Transition metal catalyzed reductive cyclization reactions of nitroarenes and nitroalkenes. *ChemCatChem* **11**, 4450–4488 (2019).

18. R. Po, G. Bianchi, C. Carbonera, A. Pellegrino, "All that glisters is not gold": An analysis of the synthetic complexity of efficient polymer donors for polymer solar cells. *Macromolecules* **48**, 453–461 (2015).

19. F. Arnáiz, R. Sanz, J. Escribano, R. Aguado, M. Pedrosa, Selective deoxygenation of sulfoxides to sulfides with phosphites catalyzed by dichlorodioxomolybdenum(VI). *Synthesis* **2004**, 1629–1632 (**2004**).

20. F. Baert, C. Cabanetos, M. Allain, V. Silvestre, P. Leriche, P. Blanchard, Thieno[2,3-b]indole-based small push–pull chromophores: Synthesis, structure, and electronic properties. *Org. Lett.* **18**, 1582–1585 (2016).

21. M. Castineira Reis, M. Marin-Luna, C. Silva Lopez, O. N. Faza, Mechanism of the molybdenum-mediated cadogan reaction. *ACS Omega* **3**, 7019–7026 (2018).

22. T. Li, L. Yang, Y. Xiao, K. Liu, J. Wang, X. Lu, X. Zhan, Facile synthesis of high-performance nonfullerene acceptor isomers via a one stone two birds strategy. *J. Mater. Chem. A* **7**, 20667–20674 (2019).

23. B. List, R. A. Lerner, C. F. Barbas, Proline-catalyzed direct asymmetric aldol reactions. *J. Am. Chem. Soc.* **122**, 2395–2396 (2000).

24. T. C. Nugent, F. Goswami, S. Debnath, I. Hussain, H. Ali El Damrany Hussein, A. Karn, S. Nakka, Harnessing Additional capability from in water reaction conditions: Aldol versus knoevenagel chemoselectivity. *Adv. Synth. Catal.* **363**, 3539–3545 (2021).

25. H. Fu, J. Yao, M. Zhang, L. Xue, Q. Zhou, S. Li, M. Lei, L. Meng, Z. G. Zhang, Y. Li, Low-cost synthesis of small molecule acceptors makes polymer solar cells commercially viable. *Nat. Commun.* **13**, 3687 (2022).

26. M. J. Frisch, G. W. Trucks, H. B. Schlegel, G. E. Scuseria, M. A. Robb, J. R. Cheeseman, G. Scalmani, V. Barone, G. A. Petersson, H. Nakatsuji, X. Li, M. Caricato, A. V. Marenich, J. Bloino, B. G. Janesko, R. Gomperts, B. Mennucci, H. P. Hratchian, J. V. Ortiz, A. F. Izmaylov, J. L. Sonnenberg, D. Williams-Young, F. Ding, F. Lipparini, F. Egidi, J. Goings, B. Peng, A. Petrone, T. Henderson, D. Ranasinghe, V. G. Zakrzewski, J. Gao, N. Rega, G. Zheng, W. Liang, M. Hada, M. Ehara, K. Toyota, R. Fukuda, J. Hasegawa, M. Ishida, T. Nakajima, Y. Honda, O. Kitao, H. Nakai, T. Vreven, K. Throssell, J. A. Montgomery, Jr., J. E. Peralta, F. Ogliaro, M. J. Bearpark, J. J. Heyd, E. N. Brothers, K. N. Kudin, V. N.

Staroverov, T. A. Keith, R. Kobayashi, J. Normand, K. Raghavachari, A. P. Rendell, J. C. Burant, S. S. Iyengar, J. Tomasi, M. Cossi, J. M. Millam, M. Klene, C. Adamo, R. Cammi, J. W. Ochterski, R. L. Martin, K. Morokuma, O. Farkas, J. B. Foresman, and D. J. Fox, Gaussian 16, Revision C.01, Gaussian Inc., Wallingford CT, 2019.

27. Y. Zhao, D. G. Truhlar, The M06 suite of density functionals for main group thermochemistry, thermochemical kinetics, noncovalent interactions, excited states, and transition elements: Two new functionals and systematic testing of four M06-class functionals and 12 other functionals. *Theor. Chem. Acc.* **120**, 215–41 (2008).

28. A. D. McLean, G. S. Chandler, Contracted Gaussian basis sets for molecular calculations. I. Second row atoms, Z=11–18. *J. Chem. Phys.*, **72**, 5639–5648 (1980).

29. Benjamin P. Pritchard, Doaa Altarawy, Brett Didier, Tara D. Gibson, Theresa L. Windus. New basis set exchange: An open, up-to-date resource for the molecular sciences community, *J. Chem. Inf. Model.* **59**, 4814–4820 (2019).

30. V. Barone, M. Cossi, Quantum calculation of molecular energies and energy gradients in solution by a conductor solvent model. *J. Phys. Chem. A* **102**, 1995–2001 (1998).

31. X. Li, F. Pan, C. Sun, M. Zhang, Z. Wang, J. Du, J. Wang, M. Xiao, L. Xue, Z.-G. Zhang, C. Zhang, F. Liu, Y. Li, Simplified synthetic routes for low cost and high photovoltaic performance n-type organic semiconductor acceptors. *Nat. Comm.* **10**, 519 (2019).

32. G. Chai, J. Zhang, M. Pan, Z. Wang, J. Yu, J. Liang, H. Yu, Y. Chen, A. Shang, X. Liu, F. Bai, R. Ma, Y. Chang, S. Luo, A. Zeng, H. Zhou, K. Chen, F. Gao, H. Ade, H. Yan, Deciphering the role of chalcogen-containing heterocycles in nonfullerene acceptors for organic solar cells. *ACS Energy Lett.* **5**, 3415–3425 (2020).

33. B. Fan, D. Zhang, M. Li, W. Zhong, Z. Zeng, L. Ying, F. Huang, Y. Cao, Achieving over 16% efficiency for single-junction organic solar cells. *Sci. China Chem.* **62**, 746–752 (2019).

34. W. Zhao, S. Li, H. Yao, S. Zhang, Y. Zhang, B. Yang, J. Hou, Molecular optimization enables over 13% efficiency in organic solar cells. *J. Am. Chem.l Soc.* **139**, 7148–7151 (2017).

35. T. Liu, Z. Luo, Y. Chen, T. Yang, Y. Xiao, G. Zhang, R. Ma, X. Lu, C. Zhan, M. Zhang, C. Yang, Y. Li, J. Yao, H. Yan, A nonfullerene acceptor with a 1000 nm absorption edge enables ternary organic solar cells with improved optical and morphological properties and efficiencies over 15%. *Energ. Environ. Sci.* **12**, 2529–2536 (2019).

36. Y. Cui, H. Yao, J. Zhang, T. Zhang, Y. Wang, L. Hong, K. Xian, B. Xu, S. Zhang, J. Peng, Z. Wei, F. Gao, J. Hou, Over 16% efficiency organic photovoltaic cells enabled by a chlorinated acceptor with increased open-circuit voltages. *Nat. Comm.* **10**, 2515 (2019).

37. J. Yuan, Y. Zhang, L. Zhou, C. Zhang, T.-K. Lau, G. Zhang, X. Lu, H.-L. Yip, S. K. So, S. Beaupré, M. Mainville, P. A. Johnson, M. Leclerc, H. Chen, H. Peng, Y. Li, Y. Zou, Fused benzothiadiazole: A building block for N-type organic acceptor to achieve high-performance organic solar cells. *Adv. Mater.* **31**, 1807577 (2019).

38. J. Jia, J. Jing, T. Jia, K. Zhang, J. Zhang, J. Zhang, F. Huang, C. Yang, The regioisomeric bromination effects of fused-ring electron acceptors: Modulation of the optoelectronic property and miscibility endowing the polymer solar cells with 15% efficiency. *J. Mater. C. A*, **8**, 25101–25108 (2020).
